# Mechanosensitive channel Piezo1 in calcium dynamics: structure, function, and emerging therapeutic strategies

**DOI:** 10.3389/fmolb.2025.1693456

**Published:** 2025-10-21

**Authors:** Yu Liu, Yu-Qiu Xu, Yu-Yin Long, Hui Xiao, Yu-Ying Ma, Yong-Wang Li

**Affiliations:** ^1^ The First Affiliated Hospital of Guangdong Pharmaceutical University, Guangzhou, Guangdong, China; ^2^ Department of Anesthesiology, The Third People’s Hospital of Longgang, Clinical Institute of Shantou University Medical College (The Third People’s Hospital of Longgang District Shenzhen), Shenzhen, Guangdong, China

**Keywords:** Piezo1, calcium ions, mechanotransduction, pathophysiology, therapeutic targets

## Abstract

Piezo1, a trimeric mechanosensitive cation channel discovered in 2010 and recognized with the 2021 Nobel Prize for its seminal role in mechanotransduction, has emerged as a key transducer of mechanical forces into calcium ions (Ca^2+^) signaling. Its distinctive propeller-like structure confers high mechanosensitivity, enabling rapid and graded Ca^2+^ influx under diverse mechanical stimuli such as shear stress, stretch, or compression. This Ca^2+^ entry establishes localized nanodomains and amplifies signals via Ca^2+^-induced Ca^2+^ release, thereby activating a spectrum of downstream effectors including CaMKII, NFAT, and YAP/TAZ. Through these pathways, Piezo1 orchestrates critical physiological processes including vascular tone, skeletal remodeling, immune responses, neural plasticity, and organ development. Conversely, its dysregulation drives numerous pathologies, ranging from hypertension and atherosclerosis to neurodegeneration, fibrosis, osteoarthritis, and cancer. Advances in pharmacological modulators (e.g., Yoda1, GsMTx4), gene-editing, and nanomedicine underscore promising therapeutic opportunities, though challenges persist in tissue specificity, off-target effects, and nonlinear Ca^2+^ dynamics. This review synthesizes current knowledge on Piezo1-mediated Ca^2+^ signaling, delineates its dual roles in physiology and disease, and evaluates emerging therapeutic strategies. Future integration of structural biology, systems mechanobiology, and artificial intelligence is poised to enable precision targeting of Piezo1 in clinical practice.

## 1 Introduction

Mechanosensitive ion channels are key molecular sensors that enable organisms to perceive and respond to mechanical stimuli, playing crucial roles in processes ranging from cell differentiation and tissue development to the maintenance of systemic homeostasis. Among these, Piezo1, one of the most important mechanosensitive cation channels, was discovered in 2010 by Ardem Patapoutian’s team through systematic functional genomic screening (Coste et al., 2010). This groundbreaking discovery not only revealed the molecular basis of mechanosensation but also earned the 2021 Nobel Prize in Physiology or Medicine due to its significant implications for physiology and medicine.

The core function of the Piezo1 channel lies in its ability to directly convert mechanical stimuli into cation influx, thereby initiating downstream signaling cascades. When cells are subjected to mechanical stimuli such as shear stress, membrane stretching, or compression, the Piezo1 channel opens within milliseconds, mediating rapid calcium ions (Ca^2+^) influx (Saotome et al., 2018; Hill et al., 2022). This process does not rely on second messenger systems, making Piezo1 a true “mechano-chemo transducer.” As a universal second messenger, Ca^2+^ activates downstream effectors such as calmodulin (CaM) and Ca^2+^/CaM -dependent kinases, thereby regulating gene expression, cell morphological remodeling, and functional differentiation (Li et al., 2014; Lew, 2025).

Under physiological conditions, Piezo1 plays a critical role in various tissues and cell types. In vascular endothelial cells, it senses blood flow-induced shear stress and regulates vascular tone through the Ca^2+^-NO pathway (Atcha et al., 2021). In red blood cells, Piezo1 maintains erythrocyte function by regulating cell volume homeostasis (Lew, 2025). In the skeletal system, it mediates osteocyte responses to mechanical load, promoting bone formation (Amado et al., 2024). Additionally, Piezo1 is involved in immune cell activation, neural synaptic plasticity, and the development of multiple organs (Jiang et al., 2022; Chu et al., 2023).

However, dysfunction of Piezo1 is closely associated with various diseases. Gain-of-function mutations lead to hereditary xerocytosis (Cordeil and Jallades, 2024; Liang et al., 2024), while overexpression or overactivation contributes to pathological processes such as hypertension, atherosclerosis, and tumor metastasis (Li M. et al., 2022; Chu et al., 2025; Knoepp et al., 2025). These findings have established Piezo1 as a potential therapeutic target for various diseases, with developed agonists and inhibitors (e.g., GsMTx4) showing promise in preclinical studies (Jiang Z. et al., 2024).

This review systematically integrates the latest advances in Piezo1 research. It begins by detailing its structure and activation mechanisms, then delves into the Ca^2+^ signaling pathways it mediates, followed by a systematic analysis of its pathophysiological mechanisms in various diseases. It comprehensively evaluates current therapeutic strategies targeting Piezo1, discusses major challenges in clinical translation, and prospectively explores innovative applications of artificial intelligence in Piezo1 research and targeted therapies. Finally, it concludes with future research directions. Through this multidimensional and systematic discussion, this review aims to provide comprehensive theoretical insights and practical guidance for both basic research and clinical translation related to Piezo1.

## 2 Structure and regulatory mechanisms of Piezo1

### 2.1 Three-dimensional architecture and gating mechanism

Piezo1 is a mechanosensitive, non-selective cation channel whose trimeric architecture has been resolved via high-resolution cryo-electron microscopy (cryo-EM) (Xu, 2016; Tang et al., 2022; Mazal et al., 2025). The channel adopts a distinctive “bowl-shaped” conformation (Lin et al., 2019; Yang et al., 2022a), featuring elongated blade-like domains that radiate symmetrically from a central pore region, resembling a three-bladed propeller. This design not only forms the ion-conducting core but also enhances mechanosensitivity through interactions between the blade regions and lipid membrane curvature (Saotome et al., 2018; Zhang et al., 2017; Zhao et al., 2018; Wang et al., 2019).

Structural analyses reveal that Piezo1 undergoes dynamic conformational transitions, including curved and flattened states (Yang et al., 2022a; Yang S. et al., 2022; Liu et al., 2025a). Membrane tension—induced by stretching—propagates through the outward flexing of the blade arms, driving the pore domain from a closed to an open state (Lin et al., 2019; Yang et al., 2022a; Lüchtefeld et al., 2024). Molecular dynamics (MD) simulations indicate that this process involves coordinated movements of critical amino acid residues, particularly conserved sites near the pore region that govern gating kinetics (Zarychanski et al., 2012; Glogowska et al., 2015; Zhang T. et al., 2024). These rapid, reversible conformational changes enable Piezo1 to activate and inactivate within milliseconds (Atcha et al., 2021; Lin et al., 2019; Harraz et al., 2022; Young et al., 2023).

The propeller-like architecture is central to Piezo1’s mechanosensitivity. Under tension, blade expansion alters the pore diameter, modulating ion selectivity and conductance (Zhao et al., 2018; Wang et al., 2019; Ge et al., 2015). This “force-structure-function” coupling establishes Piezo1 as a unique cellular mechanotransducer (Lin et al., 2019; Ma et al., 2021). Notably, Piezo1’s activity is tightly regulated by its lipid microenvironment (Bavi et al., 2019; Zhang C. et al., 2020). The channel harbors multiple lipid-binding sites, and alterations in membrane cholesterol or phospholipid composition directly modulate its mechanical threshold (Yang et al., 2022a; Cox and Gottlieb, 2019; Buyan et al., 2020; Vanderroost et al., 2023). Additionally, coupling with the cytoskeleton provides an auxiliary gating mechanism (Qian et al., 2022; Lei M. et al., 2024). The “force-from-filaments” model may explain tissue-specific responses to mechanical stimuli (Wang et al., 2023; Baratchi et al., 2024; Wang et al., 2025a). Despite being non-selective, Piezo1 exhibits a marked preference for Ca^2+^ permeation, as demonstrated by cryo-EM and electrophysiological studies (Tang et al., 2022; Mazal et al., 2025; Liu et al., 2025a; Harraz et al., 2022). This Ca^2+^ influx not only depolarizes the membrane but also initiates downstream signaling cascades. The selectivity filter’s interplay with membrane physical properties (e.g., tension, curvature) ensures robust and precise mechanotransduction across diverse physiological contexts (Table 1) (Lin et al., 2019; Yang et al., 2022a; Douguet and Honoré, 2019).

**TABLE 1:** Structural features and gating mechanism of Piezo1.

Feature	Description	Role in gating
Overall Shape	Trimeric, propeller-like structure	Curved shape senses force, flattening opens pore
Pore Domain	Central ion-conducting channel	Directly controls cation flow
Blade Region	Large arms contacting lipid membrane	Main force sensor; converts tension to pore opening
Gating Mechanism	Curved to Flattened transition	Membrane tension is the primary activator

### 2.2 Mechanical sensitivity regulation of Piezo1

Studies have shown that membrane tension and curvature are key factors in regulating Piezo1 activity. In high-tension environments, Piezo1 exhibits enhanced activity, while under low-tension conditions, the channel’s activity significantly decreases. This phenomenon suggests that the physical environment of the cell membrane directly affects Piezo1’s function, thereby influencing the cell’s response to external mechanical stimuli.

Changes in membrane curvature also significantly impact Piezo1 activity. Experimental results show that when membrane curvature increases, the activation threshold of Piezo1 is lowered, making it more responsive to mechanical stimuli (Yang S. et al., 2022; Smith et al., 2025). This mechanism plays an important role in processes like cell migration and growth, particularly in physiological processes such as tumor cell metastasis and angiogenesis (Jiang et al., 2022; Li M. et al., 2022; Guan et al., 2024; Zhang T. et al., 2025). The cytoskeleton plays a crucial role in Piezo1’s mechanical sensitivity. Research has found that the integrity of the cytoskeleton directly affects Piezo1’s activity (Qian et al., 2022; Baratchi et al., 2024; Geng et al., 2021). When the cell membrane is mechanically stretched, the contraction and extension of the cytoskeleton can regulate Piezo1’s open state, thus influencing Ca^2+^ influx (Lüchtefeld et al., 2024; Qian et al., 2022; Risinger and Kalfa, 2020). Additionally, changes in the cytoskeleton may further affect Piezo1’s function by altering membrane tension and curvature (Qian et al., 2022; Swain and Liddle, 2023; Yang et al., 2024). The interaction between the cytoskeleton and Piezo1 is not limited to direct physical contact but also includes indirect regulation through cellular signaling pathways (Chen G. et al., 2023; Chen D. et al., 2024; Liu and Dernburg, 2024). For example, under mechanical stimulation, dynamic changes in the cytoskeleton can regulate intracellular Ca^2+^ signaling, affecting the cell’s physiological behavior and pathological responses (Sugisawa et al., 2020; Ye Y. et al., 2022).

Piezo1’s function is also significantly influenced by its lipid microenvironment (Bavi et al., 2019; Zhang C. et al., 2020; Karkempetzaki and Ravid, 2024). Studies have shown that the composition and distribution of lipids in the membrane can regulate Piezo1’s activity, especially the content of phospholipids and cholesterol (Gonçalves et al., 2023; Xue et al., 2024; Contreras et al., 2025). Changes in lipids can impact Piezo1’s conformation and function, thereby influencing its response to mechanical stimuli (Coste et al., 2010; Saotome et al., 2018; Lin et al., 2019). For example, the presence of cholesterol has been found to enhance Piezo1’s mechanical sensitivity (Qi et al., 2015; Glogowska et al., 2021), while in environments deficient in cholesterol, Piezo1’s activity is significantly reduced. This finding provides a new perspective on understanding Piezo1’s function under different physiological and pathological conditions and may offer new strategies for treating diseases related to Piezo1 (Table 2) (Zhou et al., 2023; Lei L. et al., 2024; Wang et al., 2025b).

**TABLE 2:** Glossary of technical terms.

Term	Definition
Calcium Oscillations	Periodic, rhythmic changes in intracellular Ca2+ concentration that encode cellular signals
Calcium Waves	Propagating waves of Ca2+ ions that travel within or between cells for long-distance communication
Curvature	The degree of bending of a cell membrane, a physical parameter sensed by mechanosensitive proteins
Cytoskeleton	Intracellular network of protein filaments that provides structural support and transmits mechanical forces
Durotaxis	Cell migration guided by a gradient of substrate stiffness in the extracellular matrix
Lipid Microenvironment	The local composition and physical properties of lipids surrounding a membrane protein, regulating its activity
Mechanotransduction	The process by which cells convert mechanical stimuli into biochemical or electrical signals
Membrane Tension	The lateral stretching force applied to a cell membrane, a key regulator of mechanosensitive channels
Piezo1	A major mechanosensitive cation channel in vertebrates that is directly activated by membrane tension to mediate Ca2+ influx
Selectivity Filter	A critical structural region within an ion channel’s pore that determines which ions can pass through
Shear Stress	The tangential frictional force exerted by fluid flow (e.g., blood) on a cell surface (e.g., endothelium)

## 3 Piezo1-mediated Ca^2+^ signaling initiation and amplification

### 3.1 Initial triggering mechanisms of Ca^2+^ influx

Piezo1 represents a prototypical mechanosensitive cation channel that rapidly opens in response to mechanical stimuli (e.g., stretching, shear stress, or osmotic pressure changes) (Zhao et al., 2017), mediating instantaneous Ca^2+^ influx (Hu et al., 2023; Luo S. et al., 2023; Yan Z. et al., 2024). While permeable to Na^+^ and K^+^ (Shahidullah et al., 2022; Hirata et al., 2023), Piezo1 exhibits preferential Ca^2+^ conductivity - a selectivity profile consistently observed across cardiomyocytes (Jiang F. et al., 2021; Xu H. et al., 2025), vascular endothelial cells (Wang et al., 2016; Lim et al., 2024; Lim and Harraz, 2024), and immune cells (Solis et al., 2019; Ran et al., 2023; Tang et al., 2023). This ion selectivity stems from the channel’s unique three-dimensional architecture, where tension-induced conformational changes in the pore diameter and critical residues dynamically modulate Ca^2+^ affinity and permeability (Wang et al., 2019).

Upon Piezo1 activation, spatially restricted Ca^2+^ microdomains emerge as the primary signaling platforms. These transient, high-concentration Ca^2+^ nanodomains serve dual roles: (1) as the epicenters for signal initiation; (2) as amplifiers for downstream cascades (Harraz et al., 2022; Lei M. et al., 2024; Jiang F. et al., 2021; Vasileva et al., 2025). Experimental evidence demonstrates their capacity to rapidly engage Ca^2+^-dependent effectors, including calmodulin (CaM) (Geng et al., 2021; Choi et al., 2022; Chen S. et al., 2023) and Ca^2+^/CaM-dependent protein kinases (CaMKs) (Geng et al., 2021; Chen S. et al., 2023; Fei et al., 2023), thereby regulating diverse cellular processes ranging from migration and secretion to metabolic reprogramming. In cardiomyocytes, these microdomains potentiate contractility through localized Ca^2+^-induced Ca^2+^ release (CICR), while in immune cells they modulate inflammatory responses and phagocytic activity (Chi et al., 2022; Fang et al., 2023; Sun L. et al., 2025).

A critical feature of Piezo1-mediated mechanotransduction is its stimulus-strength coding capability (Choi et al., 2022; Chen S. et al., 2023; Wang et al., 2025c). The amplitude of Ca^2+^ signals exhibits positive correlation with mechanical input intensity - stronger stimuli increase both channel open probability and duration, resulting in greater Ca^2+^ influx (Zhang G. et al., 2021; Jiang T. et al., 2024). This graded response enables cells to quantitatively decode mechanical information, translating force magnitude into differential physiological outputs through variations in Ca^2+^ signal amplitude and frequency (Jiang Z. et al., 2024; Wang et al., 2025a; Zhang T. et al., 2025).

### 3.2 Secondary amplification pathways of Ca^2+^ signaling

Following the initial Ca^2+^ influx triggered by Piezo1, cells employ CICR to amplify the signal (Endo, 2009; Eisner et al., 2017; Sun et al., 2024). This process involves activation of endoplasmic reticulum (ER) Ca^2+^ channels (ryanodine receptors and IP_3_ receptors) by entering Ca^2+^, leading to massive Ca^2+^ store release that dramatically enhances both amplitude and duration of cytoplasmic Ca^2+^ signals (Vervliet et al., 2015; Woll and Van Petegem, 2022; Knutson et al., 2023). In cardiomyocytes, this mechanism is particularly crucial, as CICR directly drives sarcoplasmic reticulum Ca^2+^ release - the fundamental process underlying cardiac muscle contraction (Shan et al., 2012; Yu et al., 2022; Su et al., 2023).

The ER serves as the primary Ca^2+^ reservoir and plays a central role in Piezo1-mediated signal amplification (Jakob et al., 2021). Its Ca^2+^ release is precisely regulated by Sarco/Endoplasmic Reticulum Ca^2+^-ATPase (SERCA) pumps and associated channels, ensuring effective signal amplification while preventing Ca^2+^ toxicity from overactivation (Zhang et al., 2017; Abbonante et al., 2024; Zhao JX. et al., 2025). This delicate balance allows cells to flexibly adjust Ca^2+^ signal intensity and duration according to environmental demands.

Mitochondria also participate in Piezo1-mediated Ca^2+^ signaling through the mitochondrial Ca^2+^ uniporter (Xu H. et al., 2025; Zhang Q. et al., 2024; Zhu Y. et al., 2025). By rapidly sequestering excess Ca^2+^, mitochondria not only prevent cellular Ca^2+^ overload but also utilize Ca^2+^ to regulate TCA cycle activity and Adenosine Triphosphate (ATP) synthesis, thereby influencing energy metabolism and oxidative stress defense (Chakrabarty and Chandel, 2021; Arnold et al., 2022; Liu Y. et al., 2025). Emerging evidence reveals dynamic coupling between mitochondrial Ca^2+^ uptake and ER Ca^2+^ release, forming an intracellular ER-mitochondria functional network that ensures both signal amplification and homeostatic control (Hirabayashi et al., 2017; Thoudam et al., 2023; Li Q. et al., 2025).

**FIGURE 1:**
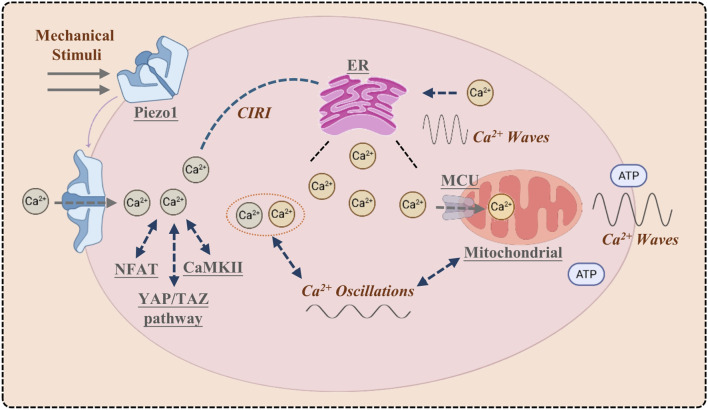
Piezo1 orchestrates the initiation and cascade of calcium signaling.

Mechanical activation of the Piezo1 channel on the plasma membrane initiates Ca^2+^ influx, generating a localized Ca^2+^ microdomain. This microdomain activates downstream signaling pathways (e.g., CaMKII, NFAT, YAP/TAZ) and triggers further Ca^2+^ release from the endoplasmic reticulum (ER) via calcium-induced calcium release (CICR). Concurrently, mitochondria uptake Ca^2+^ through the MCU to modulate metabolism and prevent Ca^2+^ overload. The resulting global elevation of cytosolic Ca^2+^ concentration propagates as Ca^2+^ oscillations and intercellular Ca^2+^ waves, facilitating signal transmission within and between cells (Figure 1).

### 3.3 Downstream signaling pathways

Piezo1-mediated Ca^2+^ influx and secondary amplification not only alter intracellular ion homeostasis but also activate multiple downstream pathways regulating cell proliferation, differentiation, migration, and pathological responses. Three key nodes have been extensively studied:

#### 3.3.1 CaMKII pathway

Ca^2+^/calmodulin-dependent protein kinase II (CaMKII), a serine/threonine kinase highly sensitive to Ca^2+^ fluctuations, becomes activated through autophosphorylation following Piezo1-mediated Ca^2+^ influx (Leng et al., 2022; Fu et al., 2025; Lin Z. et al., 2025). In cardiomyocytes, CaMKII regulates sarcoplasmic reticulum Ca^2+^ release and contraction rhythm (Yu et al., 2022; Su et al., 2023), while in neurons it participates in synaptic plasticity and memory formation (Lei M. et al., 2024; Liu et al., 2024a; Zhong et al., 2025). Notably, excessive CaMKII activation is associated with arrhythmias and cardiac remodeling, suggesting the Piezo1-CaMKII axis as a potential therapeutic target for cardiovascular diseases.

#### 3.3.2 Nuclear factor of activated T-cells signaling

The nuclear factor of activated T-cells (NFAT) represents a classical c Ca^2+^-dependent transcription factor activated through calcineurin-mediated dephosphorylation (Caulier et al., 2020; Strittmatter et al., 2021; Zhang et al., 2022). Piezo1-induced Ca^2+^ influx activates calcineurin, promoting NFAT nuclear translocation and subsequent regulation of cell fate-related genes. In immune cells, the Piezo1-NFAT pathway modulates cytokine production and immune response intensity (Caulier et al., 2020), while in vascular endothelial cells it participates in vascular tone regulation and inflammatory responses (Zhang et al., 2022). Aberrant NFAT activation is linked to tumor progression (Hope et al., 2022; Yang et al., 2022c) and fibrosis (Cai et al., 2021; Xie et al., 2022), indicating Piezo1’s potential role in pathological gene reprogramming.

#### 3.3.3 YAP/TAZ pathway

Yes-associated protein (YAP) and transcriptional co-activator with PDZ-binding motif (TAZ) are highly mechanosensitive transcriptional regulators. Piezo1-mediated Ca^2+^ signals indirectly influence YAP/TAZ nuclear localization and activity through cytoskeletal dynamics, RhoA/Rho-Associated Coiled-Coil Containing Protein Kinase pathway, and Ca^2+^-dependent kinases (Young and Reinhart-King, 2023; Rashidi et al., 2025; Xu L. et al., 2025). Under physiological conditions, this pathway regulates cell proliferation, tissue regeneration, and angiogenesis (Zanconato et al., 2016; Koo and Guan, 2018; Driskill and Pan, 2023). Pathologically, its dysregulation is associated with tumorigenesis, organ fibrosis, and vascular disorders (Zanconato et al., 2015; Li H. et al., 2022; Kiang et al., 2024). Recent studies highlight Piezo1’s role as a primary mechanosensor that converts mechanical stimuli into gene expression changes via YAP/TAZ, profoundly impacting cellular phenotypes.

### 3.4 Spatiotemporal dynamics of Ca^2+^ signaling cascades

Ca^2+^ signaling extends beyond simple ion influx or localized concentration changes, exhibiting distinct spatiotemporal dynamics that enable sophisticated encoding and decoding of external stimuli. Unlike monotonic ionic fluctuations, Ca^2+^ signals convey biological information through their frequency, amplitude, duration, and propagation patterns, which collectively determine the selective activation of downstream pathways and differential cellular responses. Intracellular Ca^2+^ signaling primarily manifests through two dynamic modalities-Ca^2+^ oscillations (Velasco-Estevez et al., 2020; Zheng et al., 2024) and Ca^2+^ waves (Sun et al., 2024; Zhang et al., 2024c) - that operate both independently and synergistically to ensure efficient cellular and tissue responses to environmental cues.

#### 3.4.1 Generation and regulation of Ca^2+^ oscillations

The spatiotemporal characteristics of Ca^2+^ signaling are best exemplified by Ca^2+^ oscillations, whose frequency and amplitude serve as fundamental coding parameters for cellular signal perception and integration. These oscillations emerge from the coordinated interplay of plasma membrane Ca^2+^ channels, intracellular Ca^2+^ stores (ER, mitochondria), Ca^2+^-binding proteins, and regulatory enzymes.

Notably, oscillations of varying frequency and amplitude can selectively activate distinct signaling pathways, enabling frequency-encoded cellular responses. For instance, certain neurons exhibit superior frequency resolution in Ca^2+^ signals compared to membrane potentials during sensory processing, highlighting Ca^2+^'s unique role in information transmission (Harraz et al., 2022; Sun et al., 2024; Harraz and Hashad, 2025). The dynamic regulation of Ca^2+^ oscillations relies on precise balance between negative and positive feedback mechanisms:

1. Negative feedback: Elevated Ca^2+^ levels activate Ca^2+^-binding proteins (e.g., calmodulin) (Clapham, 2007; Zhang Y. et al., 2023) and downstream enzymes (e.g., PP2B), which inhibit Ca^2+^ channels or enhance extrusion pumps, preventing cytotoxic overload (Korobkin et al., 2021; Lisek et al., 2024). This mechanism is particularly crucial for rhythmic excitation-contraction coupling in cardiomyocytes and pulsatile hormone secretion in endocrine cells.
2. Positive feedback: Amplification mechanisms like CICR in cardiomyocytes transform initial Ca^2+^ influx into massive ER Ca^2+^ release, enhancing contractile force (Endo, 2009; Eisner et al., 2017; Gao et al., 2024). In neuronal synapses, Ca^2+^-mediated positive feedback prolongs signal duration, facilitating synaptic plasticity underlying learning and memory.

Thus, Ca^2+^ oscillations represent the integrated output of multi-tiered regulatory circuits rather than simple ion fluxes, with their dynamic equilibrium determining signal strength, persistence, and specificity in both physiological and pathological contexts.

#### 3.4.2 Propagation and integration of Ca^2+^ waves

Beyond localized oscillations, Ca^2+^ waves constitute a fundamental mode of intra- and intercellular communication. These waves can propagate through sequential Ca^2+^ store release within single cells or synchronize cell populations via gap junctions or extracellular messengers (Velasco-Estevez et al., 2020; Zhang Y. et al., 2021; Li W. et al., 2025). Ca^2+^ waves typically initiate from focal Ca^2+^ release events through ER channels (IP_3_Rs, RyRs), subsequently amplified by transmembrane Ca^2+^ influx (McHugh et al., 2010; Carrisoza-Gaytan et al., 2023; Cheung et al., 2023). Their propagation occurs through two primary mechanisms:

1. Intracellular propagation: Within individual cells, Ca^2+^ waves distribute spatially through ER Ca^2+^ store redistribution, enabling functional compartmentalization. For example, in astrocytes, Ca^2+^ waves modulate neuron-glia interactions critical for neural information integration (Kim et al., 2022; Wang H. et al., 2025).
2. Intercellular propagation: Ca^2+^ signals traverse cell boundaries via gap junctions or diffusible messengers (e.g., ATP), enabling tissue-level coordination. In the heart, synchronized Ca^2+^ waves among cardiomyocytes are essential for maintaining rhythmic contraction and pump function (Liu et al., 2020; Querio et al., 2025).

At tissue scales, Ca^2+^ waves exhibit spatiotemporal specificity, with regional variations in propagation velocity, amplitude, and duration enabling complex zonal regulation. While this ensures precise adaptive responses physiologically, pathological wave abnormalities can lead to severe consequences including arrhythmias, epileptic seizures, or neurodegenerative progression (Su et al., 2023; Swain et al., 2020; Krivoshein et al., 2022; Garcia et al., 2023).

### 3.5 Functional manifestations in physiological systems

#### 3.5.1 Mechanical sensing in vascular endothelial cells

Piezo1 is a mechanosensitive ion channel that is widely distributed in vascular endothelial cells, where it senses shear stress from blood flow and transduces this into biological signals (Li et al., 2014; Li M. et al., 2022; Qian et al., 2022). Studies have shown that Piezo1’s response mechanism to shear stress in endothelial cells is crucial; it regulates Ca^2+^ signaling, which in turn affects endothelial cell function and health. For example, activation of Piezo1 leads to an increase in intracellular Ca^2+^ concentration, promoting a series of biological responses, such as the synthesis of nitric oxide (NO), which is essential for maintaining vascular relaxation and normal blood flow regulation (Wang et al., 2016; Albarrán-Juárez et al., 2018; Bartoli et al., 2022; Zhang et al., 2024d). Additionally, Piezo1 is involved in endothelial cells’ adaptive response to changes in blood flow, promoting endothelial cell proliferation and migration, thus playing an important role in vascular remodeling and repair (Jiang et al., 2022; Zhang et al., 2024d; Shinge et al., 2022).

In the vascular system, Piezo1 regulates vascular contraction and relaxation by sensing and responding to mechanical stretch and tension in the blood vessel wall (Harraz et al., 2022; Zhang et al., 2024d; Johnson et al., 2024). Research has shown that Piezo1 activation can affect smooth muscle cell contraction ability by regulating endothelial cell Ca^2+^ signaling, thereby influencing blood pressure and hemodynamics (Wang et al., 2016; Zeng et al., 2018; Friedrich et al., 2019; Iring et al., 2019). When the vessel is subjected to excessive tension or shear stress, activation of the Piezo1 channel not only enhances endothelial cell function but also promotes smooth muscle cell adaptive responses, further regulating overall vascular tension and function. This mechanism is particularly important in pathological conditions such as hypertension and atherosclerosis, where Piezo1 overexpression is closely related to vascular dysfunction (Douguet et al., 2019; Huang et al., 2023; Swiatlowska et al., 2024; Sun YY. et al., 2025).

Angiogenesis, the process of new blood vessel formation, has also garnered attention for Piezo1’s role. Research indicates that Piezo1 is involved in sensing mechanical signals in endothelial cells and promotes angiogenesis by regulating cell migration and proliferation. For instance, activation of the Piezo1 channel can enhance endothelial cell proliferation and lumen formation by influencing intracellular Ca^2+^ signaling, which promotes new blood vessel growth and remodeling (Swiatlowska et al., 2024; Shen et al., 2021; Yang et al., 2023). Furthermore, Piezo1 has been found to be closely related to the secretion of pro-angiogenic factors, providing a theoretical basis for its potential as a therapeutic target, particularly in treating vascular damage caused by hemodynamic changes (Li M. et al., 2022; Knoepp et al., 2025; Lim and Harraz, 2024; Zhong et al., 2023).

#### 3.5.2 Function in vascular smooth muscle cells

Piezo1’s function in vascular smooth muscle cells (VSMCs) primarily involves its role in regulating blood pressure. Studies show that activation of Piezo1 leads to Ca^2+^ influx, causing smooth muscle cell contraction, which in turn affects the diameter of blood vessels and blood pressure levels (Qian et al., 2022; Swiatlowska et al., 2024; Luu et al., 2024; Yan W. et al., 2024). In pathological conditions like hypertension, Piezo1 expression is upregulated, leading to excessive smooth muscle cell contraction and vascular remodeling, which exacerbates the increase in blood pressure. Therefore, Piezo1 plays an important role not only in regulating blood pressure under normal physiological conditions but also in pathological states (Knoepp et al., 2025; Douguet et al., 2019; Swiatlowska et al., 2024; Liao et al., 2021).

Piezo1 also plays an important role in maintaining the balance between vascular contraction and relaxation. By sensing mechanical stimuli, Piezo1 can regulate smooth muscle cell contraction, and through Ca^2+^ signaling, it can influence endothelial cell relaxation (Querio et al., 2025; Rode et al., 2017; Luo et al., 2025). Studies have shown that activation of Piezo1 enhances Ca^2+^ signaling, promoting vasoconstriction, whereas its inhibition may cause vasodilation, affecting overall hemodynamic status (Knoepp et al., 2025; Chen G. et al., 2023; Sun et al., 2024; Pathak et al., 2014).

In the process of arteriosclerosis, Piezo1 dysfunction is closely associated with pathological changes in VSMCs (Swiatlowska et al., 2024; Liu Z. et al., 2023). Research has found that Piezo1 expression is significantly upregulated in arteriosclerosis patients, which is closely related to smooth muscle cell proliferation and migration, thus promoting the progression of arteriosclerosis (Zhang et al., 2024d; Swiatlowska et al., 2024; Eisenhoffer et al., 2012). By modulating Piezo1 function, new approaches for treating arteriosclerosis may be developed.

#### 3.5.3 Function in the skeletal system

##### 3.5.3.1 Mechanotransduction in osteocytes

Bone tissue is a highly mechanically adaptive biological material, and its ability to sense and respond to external mechanical loads is a crucial component of bone physiology (Xie et al., 2023; Fish and Kulkarni, 2024; Lin CY. et al., 2025). Piezo1, as the primary mechanosensitive ion channel in osteocytes, effectively detects mechanical stimuli from bone loading and transduces them into biological signals. The expression of Piezo1 is closely related to the adaptation of bone tissue to external mechanical loads. Studies have shown that Piezo1 knockout mice exhibit skeletal deformities during development and develop osteoporosis in adulthood, highlighting the importance of Piezo1 in osteocyte function and bone health (Xu et al., 2021; Zeng et al., 2022; Ochiai et al., 2024).

Under mechanical load, the activation of Piezo1 leads to an increase in intracellular Ca^2+^ concentration, which further triggers a series of signaling processes. These signaling pathways not only promote the proliferation and differentiation of osteoblasts but also regulate osteoclast function, thereby maintaining the dynamic balance of bone metabolism. Specifically, Piezo1 regulates the gene expression of osteoblasts through CaMK and calmodulin signaling pathways, promoting the synthesis and mineralization of the bone matrix (Chen S. et al., 2023; Xie et al., 2023; Dai et al., 2024; Wang B. et al., 2024).

Osteoblast differentiation is a critical step in bone formation. Research has shown that Piezo1 activation under mechanical load promotes the expression of specific genes related to osteoblast differentiation, including Runx2 and Osterix, which are key regulatory transcription factors in the osteogenic process (Jiang et al., 2021b; Zhu et al., 2024). Additionally, the activation of Piezo1 significantly enhances the metabolic activity of osteoblasts, as evidenced by increased glycolysis, fatty acid oxidation, and other metabolic pathways, providing ample energy support for osteogenesis (Chen L. et al., 2022; Wenqiang et al., 2024; Zhang et al., 2024e; Zhou et al., 2025a).

The absence or dysfunction of Piezo1 impairs osteoblast responses to mechanical stimuli, thereby affecting bone matrix synthesis and the maintenance of bone health (Wang et al., 2020; Qin et al., 2021; Brylka et al., 2024). This suggests that Piezo1 is a key regulatory factor in bone remodeling, playing an important role not only in bone formation but also in the pathogenesis of clinical diseases such as osteoporosis and bone fracture healing.

The regulation of osteoclasts is also critical in the process of bone remodeling. Studies show that Piezo1, by sensing mechanical load, activates osteoclast precursors and enhances osteoclast differentiation and activity through the Receptor Activator of Nuclear Factor Kappa-B Ligand (RANKL) signaling pathway (Wu et al., 2021; Liu Z. et al., 2022; Uchinuma et al., 2024). In pathological conditions such as osteoporosis, Piezo1 dysfunction may lead to excessive osteoclast activity, accelerating bone loss and the progression of osteoporosis. Targeting Piezo1 function and modulating osteoclast activity could provide a new strategy for treating osteoporosis.

##### 3.5.3.2 Function in cartilage tissue

Cartilage tissue primarily functions in load buffering and friction reduction in joints. Its health and functionality are directly dependent on the chondrocytes’ ability to sense and respond to mechanical stimuli (Brylka et al., 2024; Wang et al., 2022a; Jia S. et al., 2024). As a key mechanosensitive channel in chondrocytes, Piezo1 can sense changes in joint load and regulate associated signaling pathways to maintain cartilage structure and function. Research indicates that activation of Piezo1 in chondrocytes promotes their metabolic activity, particularly under joint load, where Piezo1 helps balance the synthesis and degradation of the cartilage matrix, maintaining cartilage homeostasis (Liu Y. et al., 2022; Ren et al., 2023; Peng et al., 2025; Yu et al., 2025).

In the metabolic processes of chondrocytes, Piezo1 directly influences glucose metabolism (Brylka et al., 2024; Jia S. et al., 2024), fatty acid oxidation (Chen F. et al., 2024), and matrix synthesis (Peng et al., 2025; Nieuwstraten et al., 2025) by regulating Ca^2+^ signaling. Studies have found that the absence of Piezo1 significantly reduces the chondrocyte’s ability to tolerate mechanical load, affecting cartilage growth and repair (Brylka et al., 2024; Jia S. et al., 2024; Lee et al., 2014). This finding emphasizes the critical role of Piezo1 in regulating chondrocyte metabolism and function, particularly in degenerative cartilage diseases.

OA is a common joint disease characterized by cartilage degradation, and its occurrence and progression are closely linked to chondrocyte function (He et al., 2024; Qin et al., 2024). Research has shown that Piezo1 plays an essential role in the pathogenesis of OA (Brylka et al., 2024; Nieuwstraten et al., 2025; Wang X. et al., 2025). Mechanical stimuli activate the metabolic processes of chondrocytes via Piezo1, promoting matrix synthesis and cartilage repair. However, under pathological conditions like OA, dysfunction of Piezo1 may lead to metabolic disorders in chondrocytes (Lee W. et al., 2021), accelerating cartilage degradation (Gan et al., 2024) and joint dysfunction (Liu Y. et al., 2023). Therefore, Piezo1 could be a potential therapeutic target for OA, and targeting Piezo1 or its downstream signaling pathways may effectively intervene in cartilage degeneration and joint damage.

#### 3.5.4 Function of Piezo1 in the immune system

##### 3.5.4.1 Mechanical sensing in immune cells

Piezo1, as a mechanosensitive ion channel, plays a crucial role in the mechanical sensing of immune cells, particularly in the polarization of macrophages (Solis et al., 2019; Sun et al., 2024; Mukhopadhyay et al., 2024). Studies have shown that when macrophages are subjected to mechanical stimuli, the activation of Piezo1 promotes their polarization towards the M2 phenotype (Jiang J. et al., 2023; Zhang X. et al., 2025; Zhao Y. et al., 2025). This type of macrophage is typically anti-inflammatory and contributes to tissue repair and regeneration. In acute inflammatory environments, Piezo1 activation enhances macrophage phagocytosis and cytokine secretion, playing a key role in pathogen clearance and the regulation of inflammation. Additionally, Piezo1 affects the metabolic state and function of macrophages by regulating Ca^2+^ influx, influencing their polarization direction (Li et al., 2014; Zhao Y. et al., 2025; Cai et al., 2023). Therefore, Piezo1 is not only a critical molecule for mechanosensation in macrophages but also plays a significant role in regulating their function.

The activation and migration of T cells are also regulated by Piezo1. Research indicates that Piezo1 can sense the mechanical properties of the ECM and, through Ca^2+^ signaling pathways, influence T cell migration ability (Wang et al., 2023; Li J. et al., 2022; Zhang et al., 2024f). In the tumor microenvironment (TME), upregulation of Piezo1 expression correlates with increased T cell infiltration. Piezo1 promotes T cell adhesion and migration, facilitating their accumulation at tumor sites (Zhuang et al., 2023; Pang et al., 2024; Bonner et al., 2025). This mechanism not only affects the intensity of immune responses but may also have significant implications for the efficacy of immune checkpoint inhibitors. Enhancing Piezo1 signaling could improve T cell activity in the TME, potentially improving therapeutic outcomes (Abiff et al., 2023; Yu et al., 2024a; Yang Q. et al., 2025).

Neutrophils, as the first line of defense in the immune system, also have their inflammatory responses regulated by Piezo1. Studies have shown that activation of Piezo1 in neutrophils can enhance their response to pathogens, including increased phagocytic activity and cytokine release (Orsini et al., 2021; Wang Y. et al., 2025; Zhou et al., 2025b). In chronic inflammation models, dysfunction of Piezo1 may lead to excessive accumulation and activation of neutrophils, exacerbating inflammation. Therefore, Piezo1 plays a crucial role in mechanical sensing and inflammation regulation in neutrophils, making it a potential therapeutic target.

##### 3.5.4.2 Function in immune organs

Piezo1’s function in the thymus is primarily related to the regulation of T cell development and selection. The thymus is a crucial site for T cell maturation, and its microenvironment contains numerous mechanical signals that are sensed and transduced into the cells via Piezo1, influencing the T cell selection process (Yang et al., 2023; Zhou et al., 2025a; Yu et al., 2024a). Studies have shown that Piezo1 expression is closely associated with T cell development in the thymus, and the absence of Piezo1 leads to reduced T cell selection efficiency, impacting the diversity and function of peripheral T cells (Luo S. et al., 2023; Pang et al., 2024). This regulatory mechanism is essential for maintaining immune tolerance and preventing autoimmune diseases.

In lymph nodes, Piezo1 also plays an important role (Chang et al., 2019). Lymph nodes are crucial sites for T and B cell activation and proliferation, and their mechanical microenvironment significantly affects immune cell function (Chang et al., 2019; Shi et al., 2023; Zeng et al., 2025). Piezo1 senses mechanical signals in the lymph nodes and regulates the migration and activation state of cells, thereby enhancing the efficiency of the immune response. Additionally, Piezo1 activation is closely linked to intercellular interactions in the lymph nodes, which can have important implications for the metastasis and growth of tumor cells (Wang X. et al., 2021; Greenlee et al., 2022; Liao et al., 2024).

In the spleen, Piezo1 influences immune function by regulating the activation and proliferation of immune cells. Studies have shown that Piezo1 activation enhances the interaction between macrophages and T cells in the spleen, strengthening immune responses against infections. Furthermore, Piezo1’s function in the spleen is closely related to its role in sensing and transducing mechanical signals, offering new perspectives for improving spleen function and modulating immune responses (Evans et al., 2020; Deng et al., 2022; Yassouf et al., 2022; Zhou SL. et al., 2025).

#### 3.5.5 The nervous system

Piezo1, as a mechanosensitive ion channel, is widely distributed in the central nervous system (CNS) and plays a key role in neuronal development, synaptic plasticity, and nerve repair (Harraz et al., 2022; Segel et al., 2019; Xie Z. et al., 2025). Its main function is to sense mechanical stimuli and trigger Ca^2+^ influx, a process that regulates neuronal excitability and the dynamic stability of neural networks. Studies have shown that, in mouse models, activation of Piezo1 not only enhances neuronal sensitivity to external mechanical stimuli but also promotes axonal extension and synapse formation, thus accelerating neural regeneration (Li et al., 2021; Cudmore and Santana, 2022; Larriva-Sahd et al., 2023). Some researchers further suggested that Piezo1 is involved in neuronal migration and layer formation, which is important for cortical development. Moreover, abnormal regulation of Piezo1 may lead to defects in neural network reconstruction, associated with diseases such as epilepsy and developmental cognitive disorders.

In glial cells, the role of Piezo1 is also significant (Hu et al., 2023; Ivkovic et al., 2022; Jäntti et al., 2022). Astrocytes and microglia rely on Piezo1 to sense changes in the mechanical environment and regulate neuroinflammatory responses and metabolic homeostasis through Ca^2+^ signaling. For example, activation of Piezo1 promotes the secretion of pro-inflammatory cytokines (such as IL-6 and TNF-α), which assists in tissue clearance and repair after injury (Velasco-Estevez et al., 2020; Malko et al., 2023; Xu S. et al., 2025). However, excessive activation may exacerbate chronic inflammation and neural damage. Notably, recent studies have found that overactivation of Piezo1 in microglia is closely associated with the pathological progression of Alzheimer’s disease (AD) (Chu et al., 2023; Hu et al., 2023). It may accelerate neurodegeneration by enhancing inflammation and interfering with the clearance of amyloid plaques. Additionally, in demyelinating disease models, such as multiple sclerosis (MS), Piezo1 regulates oligodendrocyte precursor cell differentiation, suggesting its potential value in myelin regeneration (Velasco-Estevez et al., 2022; Yang K. et al., 2022).

In pain perception, Piezo1, as a transducer of mechanical and thermal signals, is critical for nociception (Kim et al., 2012). Research has shown that, in peripheral nerve injury and inflammatory pain models, Piezo1 expression is significantly upregulated, leading to increased Ca^2+^ influx and enhanced excitability in nociceptive neurons (Lei M. et al., 2024; Song et al., 2019). This results in mechanical hyperalgesia and chronic pain (Hill et al., 2022; Solis et al., 2019; Li QY. et al., 2022). Some researchers propose that Piezo1 and Piezo2 have complementary roles in pain regulation: Piezo2 primarily mediates light touch sensation, while Piezo1 plays a more prominent role in high-threshold mechanical stimuli and pathological pain (Obeidat et al., 2023; Xie et al., 2025b). This finding not only deepens our understanding of chronic pain mechanisms but also suggests that Piezo1 could be a new target for analgesic drug development.

In summary, Piezo1 maintains homeostasis and plasticity in the nervous system through multiple mechanisms, including regulating neuronal excitability, synaptic plasticity, glial cell function, and nociception. Its dysfunction may be closely related to neurodevelopmental disorders, neurodegenerative diseases, and chronic pain, providing new insights for neuroprotection and disease intervention.

#### 3.5.6 The respiratory system

Piezo1 also plays a critical role in the respiratory system, particularly in lung epithelial barrier function and airway mechanical regulation. Lung epithelial cells, as the first line of defense for gas exchange, must continuously respond to mechanical stretching and pressure changes during breathing (Fang et al., 2023; Friedrich et al., 2019; Grannemann et al., 2023). Research shows that Piezo1 can sense mechanical stretch and pressure differences, leading to Ca^2+^ influx, which promotes the expression and rearrangement of tight junction proteins (such as claudin and occludin), thereby enhancing the barrier function between epithelial cells (Jiang et al., 2021c; Zhou et al., 2021; Liu et al., 2025c). This mechanism not only helps maintain normal gas exchange but also prevents pathogens and harmful particles from invading the lower respiratory tract. Notably, in models of acute lung injury (Zhang M. et al., 2024; Xu X. et al., 2025) and acute respiratory distress syndrome, Piezo1 expression is significantly upregulated. It may exert a protective effect by enhancing barrier repair, but excessive Ca^2+^ influx and the release of inflammatory factors may worsen lung tissue damage, suggesting that Piezo1 has a dual regulatory effect in respiratory diseases (Fang et al., 2023; Grannemann et al., 2023).

In airway smooth muscle cells, Piezo1 has been confirmed as a key molecule regulating airway tension. Mechanical stress activates Piezo1 to trigger Ca^2+^ signaling, leading to smooth muscle contraction (Luo et al., 2025; Luo M. et al., 2024; Ni et al., 2024). This process is particularly prominent in pathological conditions such as asthma and chronic obstructive pulmonary disease: excessive activation of Piezo1 may lead to smooth muscle spasms and airway narrowing, causing increased respiratory resistance and airflow limitation (Zheng et al., 2024; Aranda et al., 2023). Recent studies have indicated that inhibiting Piezo1 signaling pathways can partially alleviate airway hyperresponsiveness in animal models, suggesting that Piezo1 may be a new target for treating diseases with airway hyperreactivity (Ni et al., 2024; Luo M. et al., 2023).

Additionally, the mechanical stretching and periodic pressure changes that alveoli endure during respiration also depend on Piezo1 for sensing and transduction. Piezo1 converts these mechanical signals into intracellular Ca^2+^ oscillations and related downstream signaling cascades, thus regulating respiratory rhythm, lung compliance, and gas exchange efficiency. Studies have also shown that Piezo1 is actively expressed in the pulmonary vascular endothelium, and its activation can regulate the release of NO and endothelial factors, affecting pulmonary blood flow distribution and oxygenation efficiency. This discovery provides new theoretical support for the role of Piezo1 in diseases such as hypoxic adaptation, pulmonary hypertension, and high-altitude sickness.

#### 3.5.7 The digestive system

In the digestive system, Piezo1 plays an important role in several processes, including the intestines, gastrointestinal motility, and liver function. As a mechanosensitive ion channel, it can sense changes in food, liquid, and pressure within the lumen and convert these mechanical signals into intracellular Ca^2+^ dynamics, thereby regulating various digestive physiological processes (Chen B. et al., 2023; Baghdadi et al., 2024; Luo S. et al., 2024).

In intestinal epithelial cells, Piezo1 is a core molecule in mechanotransduction. Studies have shown that Piezo1 can sense pressure and peristaltic stimuli in the intestinal lumen and regulate cell secretion through Ca^2+^ signaling (Harraz et al., 2022; Gonçalves et al., 2023; Eisenhoffer et al., 2012; Lee et al., 2022). For example, activation of Piezo1 by mechanical stimuli can promote the secretion of the gut hormone glucagon-like peptide-1 (GLP-1), which plays a key role in regulating blood glucose homeostasis, controlling appetite, and promoting insulin secretion (Gao et al., 2025). This suggests that Piezo1 not only participates in basic intestinal physiological functions but may also play a regulatory role in the development of metabolic diseases, such as obesity and type 2 diabetes (Zhu W. et al., 2022; Jacobs et al., 2024; Li Y. et al., 2024).

In the regulation of gastrointestinal motility, Piezo1’s role is also significant. It can sense the mechanical stress generated as food moves through the digestive tract and regulate Ca^2+^ flow in smooth muscle cells, triggering contraction and relaxation. Through this mechanism, Piezo1 is involved in regulating the peristaltic rhythm, movement patterns, and food propulsion speed of the gastrointestinal tract. This function is crucial for coordinating nutrient absorption and digestion. Studies have found that dysfunction of Piezo1 may be associated with pathological conditions such as irritable bowel syndrome, constipation, and gastrointestinal motility disorders (Lee JU. et al., 2021; Shin et al., 2022; Choi et al., 2024).

In the liver, Piezo1’s role is primarily related to the mechanosensation and activation of hepatic stellate cells (HSCs). HSCs are key effector cells in liver fibrosis, and their activation process is closely linked to mechanical signals (Shen et al., 2021; Zhu W. et al., 2022; Liu Y. et al., 2024). Research shows that activation of Piezo1 promotes the proliferation of HSCs and the synthesis of ECM components, such as collagen, thereby driving the development and progression of liver fibrosis (Jiang et al., 2022; Zhang et al., 2024c; Jiang D. et al., 2024). Conversely, inhibiting Piezo1 activity can reduce the extent of fibrosis, suggesting that it could be a potential new therapeutic target for antifibrotic treatment.

#### 3.5.8 The urinary system

In the urinary system, Piezo1 plays a crucial role, particularly in glomerular filtration function. The filtration function of the glomerulus depends on the mechanosensation of renal tubular epithelial cells, and Piezo1, as the main mechanoreceptor, regulates the Ca^2+^ concentration in these cells, influencing glomerular filtration rate (Chen G. et al., 2023; Li X. et al., 2022). Studies have shown that activation of Piezo1 enhances the filtration capacity of the glomerulus, which is vital for maintaining normal kidney function, especially in the kidney’s plasma clearance capacity (Amado et al., 2024; Gudipaty et al., 2017; Zhao et al., 2022). Additionally, Piezo1 plays an important role in the mechanical response of renal tubules, highlighting its central role in regulating overall kidney function.

In the bladder, Piezo1, as the main mechanoreceptor, is responsible for sensing the expansion and filling of the bladder. Mechanical stretching of the bladder is sensed by Piezo1, which generates Ca^2+^ signals and transmits them to the CNS, thus regulating the micturition reflex. This process is crucial for normal urinary function, ensuring the timely expulsion of urine (Chen G. et al., 2023; Michishita et al., 2016; Liu et al., 2024c). Studies have shown that dysfunction of Piezo1 may lead to bladder disorders, such as overactive bladder (OAB), a common condition characterized by frequent urgency and incontinence. The regulatory role of Piezo1 provides a new potential target for the treatment of bladder-related diseases, especially OAB.

Additionally, Piezo1 plays a key role in the mechanical sensitivity of the urinary tract. Research has found that activation of Piezo1 triggers Ca^2+^ signal transmission in the urethra, regulating the contraction and relaxation of the urinary tract (Amado et al., 2024; Dalghi et al., 2019). This mechanism not only ensures normal urination but also helps maintain urethral tension and smooth muscle function. Dysfunction of Piezo1 may lead to urinary tract disorders, affecting normal urination and potentially being associated with conditions such as incontinence and urethral obstruction.

#### 3.5.9 The endocrine system

In the endocrine system, Piezo1 plays a key role in regulating the function of pancreatic β-cells (Matute et al., 2020). Studies have shown that Piezo1 can sense mechanical signals and metabolic changes in the extracellular environment and influence insulin secretion by regulating Ca^2+^ influx. When blood glucose levels rise, activation of Piezo1 enhances Ca^2+^ signaling within β-cells, promoting insulin release to maintain blood glucose homeostasis (Ye Y. et al., 2022; Zhang M. et al., 2021). This process is crucial for preventing diabetes and related metabolic disorders. Furthermore, abnormal Piezo1 function may lead to secretion defects in β-cells, further exacerbating hyperglycemia (Zhang et al., 2024d; Ganugula et al., 2023).

In adipose tissue, Piezo1 regulates the metabolic state of adipocytes by sensing the mechanical microenvironment, such as cell volume changes and matrix stiffness (Miron et al., 2022; Leng et al., 2025). Research has found that activation of Piezo1 can affect adipocyte differentiation and lipid metabolism, thereby achieving a dynamic balance between energy storage and energy expenditure. When Piezo1 function is impaired, metabolic dysregulation of adipocytes may lead to obesity and metabolic syndrome, suggesting that Piezo1 plays a potential pathological role in the development of metabolic diseases (Rendon et al., 2022; Byun et al., 2024; Catalán et al., 2024).

Additionally, Piezo1 is involved in the regulation of the secretion of various endocrine hormones. For example, studies have shown that its activation can promote the release of hormones such as GLP-1 and insulin (Li M. et al., 2022; Ye Y. et al., 2022; Gao et al., 2025). These hormones not only play a role in blood glucose control but also participate in appetite regulation, energy metabolism, and gastrointestinal function. Therefore, Piezo1 not only functions at the level of individual organs or cells but also influences overall metabolic homeostasis through inter-organ signaling networks.

## 4 The role of Piezo1-mediated Ca^2+^ signaling in different disease states

### 4.1 Pathological role in cardiovascular diseases

#### 4.1.1 Atherosclerosis

Atherosclerosis is a complex chronic inflammatory disease closely associated with changes in the hemodynamic environment, particularly the shear stress of blood flow. As a key mechanosensitive ion channel, Piezo1 can sense blood flow shear stress and regulate endothelial cell function (Chu et al., 2025; Shinge et al., 2022; Wang YM. et al., 2024). Under blood flow stimulation, the opening of the Piezo1 channel leads to an increase in intracellular Ca^2+^ concentration, which activates inflammatory responses and cell proliferation, accelerating the formation of atherosclerosis (Zhang L. et al., 2020). In animal experiments, it has been found that Piezo1 expression is significantly upregulated in atherosclerotic mouse models, and this upregulation is closely related to endothelial cell inflammation and plaque formation (Zhang C. et al., 2020; Zhang C. et al., 2025).

Mechanistic studies show that Piezo1 not only regulates endothelial cell function by directly mediating Ca^2+^ influx but also activates downstream signaling pathways such as Ca^2+^/CaM/CaMKII, promoting the release of inflammatory factors, leading to endothelial damage and driving the progression of atherosclerosis (Hao et al., 2024; Lan et al., 2024). Inhibition of Piezo1 activity can significantly reduce endothelial inflammation induced by shear stress, suggesting that it may become an important therapeutic target for atherosclerosis treatment (Albarrán-Juárez et al., 2018; Shinge et al., 2022; Wang et al., 2022b).

It is worth noting that Piezo1-mediated Ca^2+^ signaling plays a “double-edged sword” role in plaque formation and stability. On one hand, a high Ca^2+^ environment promotes macrophages to take up cholesterol and transform into foam cells, accelerating plaque growth. On the other hand, excessive activation of Piezo1 increases the release of inflammatory mediators, weakening the stability of the fibrous cap, thus increasing the risk of plaque rupture, which could trigger acute cardiovascular events. Accordingly, inhibiting Piezo1 activity may reduce inflammation and enhance plaque stability (Choi et al., 2022; Chen S. et al., 2023; Sun et al., 2024). For example, experiments using the Piezo1 inhibitor Grammostola spatulata Mechanotoxin 4 (GsMTx4) have shown that it effectively reduces plaque inflammation levels and instability, thus exerting a protective effect (Jiang Z. et al., 2024; Wang et al., 2022a).

In terms of pharmacological intervention, recent studies have made significant progress. Yoda1, an agonist of Piezo1, can exacerbate endothelial inflammation, while the inhibitor GsMTx4 shows potential in inhibiting plaque formation and inflammation (Choi et al., 2022; Gan et al., 2024; Jiang M. et al., 2023). Additionally, some natural products, such as quercetin, have been found to alleviate the progression of atherosclerosis by downregulating Piezo1-mediated Ca^2+^ signaling, showing promising potential for application (Wang YM. et al., 2024; Guo et al., 2024). Piezo1 serves as both a pathogenic promoting factor and a potential therapeutic target in the development of atherosclerosis. More preclinical and clinical trials are needed in the future to validate the safety and efficacy of Piezo1 inhibitors or modulators, with the aim of providing new approaches and strategies for precision treatment of atherosclerosis.

Overall, Piezo1 plays a core role in atherosclerosis, bridging the gap between mechanosensation and pathological signaling cascades. Its role is not limited to endothelial cells but extends to various vascular-related cell types such as smooth muscle cells, macrophages, and fibroblasts (Qian et al., 2022; Xie Z. et al., 2025). By precisely regulating Ca^2+^signaling, Piezo1 bridges the gap between hemodynamics and vascular inflammatory responses. This characteristic provides a new biological mechanism to explain the localized distribution of atherosclerosis (Garcia et al., 2023; Rong et al., 2024) (e.g., lesions occurring more frequently at arterial bifurcations or areas of disturbed blood flow).

More importantly, Piezo1-mediated mechanotransduction signals interact with transcription factors and epigenetic regulatory networks, suggesting that Piezo1 not only serves as a “signal input” but may also deeply participate in cellular phenotype reprogramming and immune-metabolic coupling. This offers a broader perspective for understanding the systemic pathology of atherosclerosis (Chu et al., 2025; Wu et al., 2022; Yang Y. et al., 2022; Zhang FR. et al., 2025).

On the clinical translation level, future research on Piezo1 should focus on individual differences in mechanobiology. For example, Piezo1 expression or function may vary with age, gender, blood pressure status, and genetic polymorphisms, meaning that its use as a therapeutic target requires consideration of precision stratification. Furthermore, the synergistic or complementary actions between Piezo1 and other mechanosensitive channels may also affect the intervention outcomes, providing ideas for the development of multi-target combination therapy strategies (Douguet et al., 2019; Beech and Kalli, 2019; Shah et al., 2022; Duan et al., 2025).

#### 4.1.2 Hypertension

The onset of hypertension is closely associated with the abnormal activation of Piezo1 in VSMCs (Knoepp et al., 2025; Douguet et al., 2019; Retailleau et al., 2015). Piezo1 plays a central role in the vascular wall’s response to changes in blood pressure. When blood pressure increases, the mechanical stress on the vascular wall intensifies, activating the Piezo1 channel and triggering a rapid influx of Ca^2+^. This process not only directly causes the contraction of vascular smooth muscle and an increase in vascular tension but also induces functional disturbances in smooth muscle cells, thereby promoting vascular remodeling and the progression of hypertension (Knoepp et al., 2025; Chen et al., 2022b). Upregulation of Piezo1 has been observed in various hypertensive animal models, closely correlating with pathological proliferation of VSMCs and vascular stiffening.

At the molecular level, Piezo1 mediates Ca^2+^ signaling that activates downstream pathways such as AKT, Extracellular Signal-Regulated Kin (ERK), and CaMKII, promoting VSMC proliferation, migration, and phenotypic transition (Zhang et al., 2024d; Wang Z. et al., 2021; Porto Ribeiro et al., 2022). This mechanostress-driven signaling network accelerates the structural remodeling of the vascular wall, gradually transforming the vessel from a compliant state to a rigid state (Qian et al., 2022). Additionally, Piezo1 activation can affect endothelial cells, triggering the release of inflammatory factors and oxidative stress responses, further damaging endothelial function and creating a vicious cycle of endothelial dysfunction, smooth muscle remodeling, and increased blood pressure.

More notably, Piezo1’s positive feedback mechanism has an amplifying effect during the course of hypertension. Persistent high mechanical stress induces the upregulation of Piezo1 expression, making it more sensitive and thereby exacerbating VSMC responses to pressure stimuli. In the long term, this imbalance not only drives local vascular remodeling but also increases the risk of systemic cardiovascular complications, such as left ventricular hypertrophy, atherosclerosis, and kidney damage (Chu et al., 2025; Li W. et al., 2025; Shinge et al., 2022; Chen et al., 2022c).

In terms of intervention, Piezo1 has emerged as a potential therapeutic target for hypertension. Studies have shown that Piezo1 inhibitors can significantly reduce the influx of Ca^2+^ into VSMCs, lower vascular tension, and improve vascular function (Fei et al., 2023; Wang Z. et al., 2021; Zhao et al., 2021). In animal experiments, inhibiting Piezo1 not only reduces blood pressure but also alleviates hypertension-related vascular remodeling and heart damage. Furthermore, Piezo1 inhibitors may have a synergistic effect when combined with traditional antihypertensive drugs (such as Ca^2+^ channel blockers or ACE inhibitors/ARBs), improving vascular function through dual pathways (Contreras et al., 2025; Sun et al., 2024; Konishi et al., 2024).

Looking ahead, research on Piezo1 offers new insights into the mechanobiological explanation of hypertension. Its role is not only limited to regulating vascular tension but also extends to various dimensions, including metabolic state, immune inflammation, and renal blood flow regulation. For example, some studies suggest that Piezo1 is involved in mechanosensing in podocytes and renal tubular epithelial cells, potentially promoting hypertension development by affecting sodium excretion and renal blood flow perfusion. Moreover, genetic polymorphisms in Piezo1 function may help explain the differences in hypertension susceptibility and drug response across different populations.

#### 4.1.3 Myocardial hypertrophy and heart failure

Myocardial hypertrophy is an important pathological basis for the development of heart failure, characterized by an increase in myocardial cell volume and structural remodeling of the heart. Recent studies have shown that Piezo1, as a mechanosensitive ion channel, plays a key role in the heart’s response to pressure overload. When cardiomyocytes are subjected to prolonged mechanical stretch or elevated pressure, the activation of Piezo1 leads to an enhanced Ca^2+^ influx, which triggers a series of downstream signaling pathways, driving pathological myocardial hypertrophy and dysfunction (Yu et al., 2022; Sun YY. et al., 2025; Lim, 2022).

At the molecular level, Piezo1-mediated Ca^2+^ signaling can activate pathways such as AKT, ERK, and CaMKII, promoting hypertrophic growth and metabolic reprogramming of cardiomyocytes. Meanwhile, the Ca^2+^ signal also enhances the transcriptional activity of NFAT, further driving the expression of genes associated with myocardial hypertrophy (Hope et al., 2022; Ezzo et al., 2024). In addition to these classical pathways, studies have found that Piezo1 interacts with the Hippo-YAP/TAZ pathway, enhancing fibrosis and ECM deposition, ultimately accelerating myocardial remodeling and the onset of heart failure (Jiang F. et al., 2021; Sun et al., 2024; Beech and Kalli, 2019).

Pathological myocardial remodeling is not only reflected in increased cell volume but also involves cardiomyocyte apoptosis, inflammatory responses, and fibrosis. Overactivation of Piezo1 can promote the release of inflammatory factors, induce cardiomyocyte apoptosis, and enhance fibroblast activity, thereby worsening myocardial fibrosis and decreasing compliance. This also explains the common vicious cycle of inflammation-apoptosis-fibrosis in patients with heart failure.

In recent years, targeted interventions against Piezo1 have shown promising results in animal models of myocardial hypertrophy and heart failure. Studies have shown that the use of Piezo1 inhibitors can significantly alleviate pressure overload-induced myocardial hypertrophy, reduce heart weight, and improve both ventricular systolic function and diastolic compliance (Yuan et al., 2023; Merten et al., 2024). In hypertension-related myocardial hypertrophy models, the inhibition of Piezo1 not only improved structural remodeling but also reduced the degree of myocardial fibrosis, suggesting its multi-faceted protective effects on heart function (Yu et al., 2022; Lim, 2022).

Importantly, targeting Piezo1 could complement traditional drugs (such as β-blockers, ACE inhibitors/ARBs, and Ca^2+^ channel blockers). While traditional drugs mainly act on the neuroendocrine pathways, Piezo1 inhibitors target mechanotransduction pathways. Combining these approaches could provide multi-pathway intervention, enhancing the therapeutic effect for heart failure (Peng et al., 2024; Park et al., 2025).

Looking ahead, research on Piezo1 in cardiac diseases extends beyond pathological myocardial hypertrophy and heart failure and may also relate to cardiac regeneration, metabolic remodeling, and arrhythmias. For example, some studies suggest that Piezo1-mediated Ca^2+^ signaling may affect myocardial energy metabolism and mitochondrial function, thereby regulating cardiomyocyte tolerance. Furthermore, Piezo1 gene polymorphisms may influence an individual’s susceptibility to pressure overload, providing new directions for precision medicine and personalized interventions.

### 4.2 Pathological role in neurological diseases

Piezo1, as a mechanosensitive ion channel, not only plays an crucials role in the cardiovascular system but has also gained increasing attention in the occurrence and progression of neurological diseases (Bryniarska-Kubiak et al., 2023; Qu et al., 2023). Given that both neurons and glial cells are highly sensitive to mechanical stress, abnormal Piezo1-mediated Ca^2+^ signaling plays a critical role in various neurological diseases, including neurodegenerative diseases, neuropathic pain, and stroke. These findings provide important insights for elucidating the pathological mechanisms of neurological diseases and identifying novel intervention targets (Chi et al., 2022; Jäntti et al., 2022; Brandt and Smith, 2023).

#### 4.2.1 Neurodegenerative diseases

In neurodegenerative diseases such as AD and Parkinson’s disease (PD), the dysfunction of Piezo1 is considered a major driving factor for neuronal injury and death. Research has shown that Piezo1 activation leads to excessive neuronal sensitivity to mechanical stimuli, causing abnormal Ca^2+^ influx and activating multiple downstream pathways, ultimately triggering apoptosis and synaptic dysfunction. In AD, Aβ deposition and the associated inflammatory microenvironment may further enhance Piezo1 activity, accelerating the neuronal degeneration process (Hu et al., 2023; Sitnikova et al., 2025).

Piezo1 also plays an important role in glial cells. The mechanosensitization of microglia through Piezo1 can activate inflammation and the NLR Family Pyrin Domain Containing 3 inflammasome, promoting the release of pro-inflammatory cytokines and exacerbating neuroinflammation and neuronal damage (Ran et al., 2023; Pan et al., 2024). This not only disrupts the homeostasis of the neuroenvironment but may also be associated with the rate of disease progression. Animal studies have shown that Piezo1 antagonists can effectively inhibit inflammation, protect neurons, and suggest its potential clinical application value in neuroprotection and delaying the progression of neurodegenerative diseases (Sun et al., 2024; Xu T. et al., 2025).

#### 4.2.2 Neuropathic pain

Piezo1 plays a central role in the mechanosensitization of nociceptors. Its activation can trigger Ca^2+^ signaling under mechanical stimulation, enhancing neuronal excitability and amplifying pain signal transmission. For example, in trigeminal neurons, high expression of Piezo1 is closely related to mechanical stimulation-induced hyperalgesia and abnormal pain responses (Mikhailov et al., 2022; Della Pietra et al., 2023).

More importantly, pharmacological intervention targeting Piezo1 has shown significant analgesic effects. Studies have indicated that Piezo1 antagonists can effectively relieve pain responses caused by mechanical stress, especially in neuropathic pain models where traditional analgesics have limited efficacy (Li QY. et al., 2022; Cho et al., 2023). This provides a potential new approach for developing non-opioid analgesic strategies, avoiding the risks of tolerance and addiction associated with long-term opioid use.

#### 4.2.3 Stroke

In stroke, especially in the context of ischemia-reperfusion injury (IRI), abnormal activation of Piezo1 is highly associated with neuronal damage. The ischemic state leads to mechanical stress and local environmental changes, inducing the overexpression and excessive activation of Piezo1, resulting in Ca^2+^ overload, which activates cell death-related pathways (such as caspase and mitochondrial apoptosis pathways), exacerbating neuronal injury (Fu et al., 2025; Shen et al., 2021; Tang L. et al., 2024).

More critically, Piezo1 plays an important role in the disruption of the blood-brain barrier (BBB). Ischemia, hypoxia, and mechanical stress can activate Piezo1, increasing endothelial cell permeability, leading to BBB dysfunction and amplifying neuroinflammation. Animal studies have shown that the application of Piezo1 inhibitors significantly reduces BBB permeability, alleviates edema and inflammation, and protects brain tissue from further damage (Fu et al., 2025; Lai et al., 2022; Xu et al., 2024).

Furthermore, studies emphasize the importance of the timing of intervention. Early inhibition of Piezo1 activity may effectively block the “Ca^2+^ signaling storm” of IRI, thereby reducing damage before the complete breakdown of the blood-brain barrier (Fu et al., 2025; Yue et al., 2024; Scorza et al., 2025). This finding suggests that Piezo1 could become a new therapeutic target for acute stroke treatment.

Piezo1 plays the role of a “mechanical signal transducer” in different pathological processes of neurological diseases: promoting neuronal apoptosis and neuroinflammation in neurodegenerative diseases, enhancing pain signal transmission in neuropathic pain, and exacerbating Ca^2+^ overload and BBB disruption in stroke. These pieces of evidence indicate that targeting Piezo1 not only aids in understanding disease mechanisms but also provides novel therapeutic approaches for neuroprotection, analgesia, and stroke intervention (Xiao, 2024).

### 4.3 Pathological role in skeletal system diseases

Piezo1, as a key mechanosensitive ion channel in bone and joint tissues, plays a central role in transducing mechanical signals into cellular biological responses. Its abnormal expression or functional defects are closely associated with various skeletal system diseases, including osteoporosis and OA. By regulating Ca^2+^ signaling pathways, differentiation potential, and ECM homeostasis in osteoblasts and chondrocytes, Piezo1 plays an irreplaceable role in bone metabolism and the maintenance of joint homeostasis.

#### 4.3.1 Osteoporosis

Osteoblasts are the key executors of bone formation, and their response to mechanical load is crucial in maintaining bone mass and mechanical strength (Ochiai et al., 2024; Huang et al., 2024; Webster, 2025). Studies have shown that Piezo1, as the primary mechanosensor in osteoblasts, mediates rapid Ca^2+^ influx under mechanical stimulation, thereby activating signaling pathways such as PI3K/Akt, ERK, and Wnt/β-catenin, promoting osteoblast proliferation, differentiation, and mineralization. In mouse models, Piezo1 deficiency significantly weakened mechanical load-induced bone formation, leading to decreased bone mass and increased fracture risk (Tang H. et al., 2024; Zhan et al., 2024). Clinical studies have also found that Piezo1 expression is generally downregulated in osteoporosis patients, suggesting its key role in bone metabolic homeostasis. In addition to directly promoting osteogenesis, Piezo1 also enhances osteoblast adaptability to different stress environments by regulating the mechanical adaptability of the cytoskeleton. This defect in mechanosensitivity is considered one of the important causes of insufficient bone formation in the elderly and osteoporosis patients.

In recent years, studies on Piezo1 agonists have shown promising translational prospects. The specific Piezo1 agonist Yoda1 enhances osteoblast mineralization ability *in vitro* and significantly increases bone density in mouse models, partially reversing osteoporosis (Guan et al., 2024; Ochiai et al., 2024; Hao et al., 2024). Furthermore, Piezo1 agonists may provide a novel therapeutic strategy that surpasses traditional anti-resorptive drugs (such as bisphosphonates) by improving bone microstructure and enhancing bone mechanical adaptability. Therefore, Piezo1 is gradually being considered a new target for osteoporosis intervention and may promote the development of personalized osteoporosis treatments in the future.

#### 4.3.2 Osteoarthritis

Chondrocytes sense mechanical stimuli to maintain the integrity and function of articular cartilage, and Piezo1 plays a central role in this process. Under normal conditions, moderate activation of Piezo1 helps chondrocytes proliferate and secrete ECM components (such as type II collagen and proteoglycans), thereby maintaining the biomechanical properties of the joint (Savadipour et al., 2023; Sun et al., 2023; Feng et al., 2024; Jia Z. et al., 2024). However, in OA, abnormal mechanical stress leads to excessive activation of Piezo1 channels, resulting in sustained Ca^2+^ influx and activation of matrix degradation pathways such as matrix metalloproteinases (Nieuwstraten et al., 2025) and a disintegrin and metalloproteinase with thrombospondin motifs, which in turn destroy the cartilage matrix structure, induce chondrocyte apoptosis, and trigger joint degeneration (Lohberger et al., 2019; Li MJ. et al., 2024). Conversely, some studies have also observed downregulation of Piezo1 expression in OA chondrocytes, suggesting that its function may exhibit stage-specific or biphasic regulation: early excessive activation accelerates cartilage damage, while late downregulation weakens cartilage repair capacity.

Pharmacological regulation of Piezo1 has shown potential in OA intervention. Studies have shown that Piezo1 agonists can partially enhance chondrocyte function and alleviate apoptosis caused by abnormal stress, while Piezo1 inhibitors have shown effects in animal models such as reducing joint damage, lowering inflammation levels, and improving joint function (Wang et al., 2022a; Yu et al., 2025; Wang X. et al., 2025). This suggests that employing a dual regulation strategy (early inhibition, late activation) based on the progression stage of OA may become an innovative precision treatment approach.

### 4.4 Pathological role in hematopoietic system diseases

Piezo1 plays diverse and critical roles in the hematopoietic system, and its dysfunction is closely linked to various blood disorders (Scapin et al., 2025). One of the most well-established pathological associations is with hereditary xerocytosis. This condition is driven by gain-of-function mutations in Piezo1 (e.g., R2456H), which lower the mechanical activation threshold of the channel, leading to sustained Ca^2+^ influx in red blood cells upon exposure to shear stress in the circulation (Andolfo et al., 2013; Rosato et al., 2025). This activates the Gardos channel, causing K^+^ efflux and cellular dehydration, while simultaneously disrupting the cytoskeleton via calpain activation, ultimately resulting in reduced red blood cell lifespan and hemolytic anemia.

Beyond its role in red blood cells, Piezo1 significantly influences platelet function. Ca^2+^ signaling triggered by Piezo1 activation in response to hemodynamic shear stress can enhance platelet activation and aggregation, potentially promoting arterial thrombosis (Lew, 2025; Zhu W. et al., 2022). Furthermore, Piezo1 serves as a crucial mechanosensor in innate immune cells such as macrophages and neutrophils. For instance, in macrophages, Piezo1 senses the physical properties of pathogens or cellular debris, regulating phagocytosis and inflammasome activation; in neutrophils, it mediates mechanosensation during transendothelial migration, influencing their recruitment to inflammatory sites (Mukhopadhyay et al., 2024; Xie Z. et al., 2025). Thus, by modulating the mechanobiological behavior of various blood cells, Piezo1 plays a central role in maintaining hematopoietic homeostasis and immune defense.

### 4.5 Tumor formation and metastasis

Piezo1, as a key mechanosensitive ion channel, plays a crucial role in the initiation, progression, and metastasis of tumors. By converting mechanical signals from the TME (such as matrix stiffness, fluid shear stress, and intercellular tension) into Ca^2+^ influx, Piezo1 activates multiple downstream signaling pathways, regulating tumor cell proliferation, migration, invasion, and immune evasion. Therefore, Piezo1 is not only an important regulator in tumor biology but also a potential novel target for anti-tumor therapy (Chen B. et al., 2023; Dombroski et al., 2021; Xiong et al., 2022; Cui et al., 2025; Zhu B. et al., 2025).

#### 4.5.1 TME regulation

The mechanical properties of the TME play a decisive role in tumor progression. Increases in matrix stiffness, stromal fibrosis, and abnormal hemodynamics can all be sensed and transduced by tumor cells via Piezo1 channels (Jiang et al., 2022; Zhang T. et al., 2025; Zhu B. et al., 2025; Chen et al., 2018).

Studies have shown that enhanced matrix stiffness can continuously activate Piezo1, leading to Ca^2+^ influx, which in turn activates signaling pathways such as Src family kinases and YAP/TAZ, ultimately driving tumor cell proliferation, migration, and invasion (Kim et al., 2022; Yu et al., 2024b). For instance, in prostate cancer cells, fluid shear stress activates the YAP pathway through Piezo1, enhancing cell motility and clonogenic ability. In pancreatic cancer, overexpression of Piezo1 is closely associated with tumor malignancy, drug resistance, and poor patient prognosis (Swain et al., 2022; Xie et al., 2025c).

In addition to promoting the tumor cells’ own adaptability, Piezo1 also influences cancer progression by regulating the tumor immune microenvironment. Research has found that Piezo1 regulates the polarization of tumor-associated macrophages, promoting the formation of the M2 immune-suppressive phenotype, thereby inhibiting anti-tumor immune responses (Yang et al., 2024; Zhang M. et al., 2024; Qu and Zhang, 2025). Moreover, Piezo1’s role in regulating the mechanical response of tumor stromal cells and vascular endothelial cells is significant, as its activation can promote angiogenesis and nutrient supply, further supporting continuous tumor growth.

In terms of therapeutic strategies, Piezo1 inhibitors (such as siRNA interference or small molecule antagonists) have shown potential *in vitro* and in animal models to suppress tumor growth and enhance chemotherapy sensitivity (Coste et al., 2010; Wu et al., 2015). This suggests that combining Piezo1 inhibitors with immunotherapy may improve patient treatment responses and extend survival.

#### 4.5.2 Metastatic process

Tumor metastasis is the leading cause of cancer-related death, and circulating tumor cells (CTCs) face intense mechanical stress during this process, including blood flow shear stress, capillary compression, and ECM resistance. Piezo1, as the core sensor of mechanical stress, plays a key role in CTC survival, migration, and colonization.

In the early stages of metastasis, the hemodynamic environment activates Piezo1 to promote Ca^2+^ influx, thereby enhancing CTCs’ anti-apoptotic ability and adhesion, increasing their survival rate in the bloodstream (Friedrich et al., 2019; Greenlee et al., 2022; Qi et al., 2024). For example, experiments have shown that Piezo1 activation in the blood flow environment can enhance CTCs migration and the likelihood of colonization in distant organs.

In the later stages of metastasis, Piezo1 regulates cytoskeletal remodeling, integrin signaling, and ECM-cell interactions, enhancing CTCs adhesion and invasion abilities in distant organ microenvironments (Bonner et al., 2025; Lopez-Cavestany et al., 2023). In pancreatic cancer models, Piezo1 has been shown to promote CTCs colonization in the liver and lungs and increase the survival ability of metastatic sites by interacting with immune cells.

Blocking Piezo1 signaling is considered an important strategy to inhibit metastasis. Studies have used small molecule inhibitors or gene silencing techniques to suppress Piezo1, resulting in a significant reduction in CTCs migration and colonization abilities. For example, in breast cancer models, Piezo1 inhibition effectively reduced the number of lung metastases, suggesting its significant clinical value as a metastatic intervention target. In the future, Piezo1 inhibitors are expected to be used in combination with anti-angiogenic drugs or immune checkpoint inhibitors to further improve the treatment of metastatic tumors (Table 3) (O'Callaghan et al., 2022; Li B. et al., 2024; Poole et al., 2024; So et al., 2024).

**TABLE 3:** Piezo1 in disease.

System	Key diseases	Core mechanism
Cardiovascular	Atherosclerosis, Hypertension	Mediates force-induced inflammation and contraction
Nervous	Neurodegeneration, Stroke	Drives inflammation, cell death, and pain signaling
Skeletal	Osteoporosis, Osteoarthritis	Dysregulated mechanosensing leads to tissue breakdown
Cancer	Various solid tumors	Matrix stiffness activates pro-tumor pathways
Respiratory	Lung Injury (ARDS), Asthma	Mediates ventilator-induced injury and airway contraction
Urinary	Fibrosis, Overactive Bladder	Promotes scarring and excessive stretch sensing

## 5 Current therapeutic strategies targeting Piezo1

Currently, therapeutic strategies targeting Piezo1 have shown significant potential in multiple disease models, particularly in mechanosensitive and inflammation-related diseases. Piezo1 agonists and antagonists, such as Yoda1 and GsMTx4, have been extensively studied and demonstrated promising therapeutic effects. As an agonist of Piezo1, Yoda1 promotes Ca^2+^ influx, activating downstream signaling pathways to regulate cellular physiological functions. For example, Yoda1 has shown protective effects in a FK model by activating Ca^2+^ signaling, further activating the Pyrin inflammasome, and enhancing macrophage phagocytosis (Jiang Z. et al., 2024; Ran et al., 2023; Fish and Kulkarni, 2024; Zhao et al., 2022). This mechanism underscores the importance of Piezo1 in immune responses, and modulating its activity can effectively improve immune function. However, Yoda1 may cause excessive Ca^2+^ influx in some cell types, potentially leading to cytotoxic reactions, and its potential side effects and safety need to be carefully evaluated.

In contrast, GsMTx4, as a Piezo1 antagonist, inhibits Piezo1 activity, thereby alleviating cell damage caused by mechanical stimuli. Studies show that GsMTx4 effectively improves cell death and inflammation in a pancreatitis model by reducing Ca^2+^ influx and protecting cell function (Lin Z. et al., 2025; Wang et al., 2022a; He et al., 2021). This suggests that GsMTx4, as an inhibitor, holds considerable clinical potential for reducing cell damage induced by mechanical stress. However, the pharmacokinetics and administration methods of GsMTx4 in clinical applications need further exploration to ensure its efficacy and safety.

### 5.1 Diversity and adaptability of intervention strategies

Therapeutic strategies targeting Piezo1 are not limited to small molecule drugs but also include innovative methods such as gene therapy and mechanical stimulation. Gene therapy research is gradually expanding its clinical application prospects. Using CRISPR-Cas9 technology or RNA interference, researchers can specifically knock out or overexpress Piezo1 to investigate its biological roles in different pathological states (Dubin et al., 2017; Hong et al., 2017; Karamatic Crew et al., 2023). This approach shows great potential in basic research and provides a more refined observation of Piezo1’s role in diseases. However, gene therapy still faces challenges in clinical application, including safety issues, ethical concerns, and regulatory requirements for clinical implementation (Lee W. et al., 2021; Zhu Z. et al., 2022; Zhao et al., 2024).

Mechanical stimulation is another unique intervention strategy. Studies have shown that mechanical stress can influence cellular physiological states through the Piezo1 channel. For example, in chondrocytes, Ca^2+^ signaling triggered by mechanical load can lead to cell apoptosis and matrix degradation, which is significant in pathological conditions like arthritis (Lee et al., 2014; Wang X. et al., 2024; Yuan et al., 2025)s. Therefore, combining mechanical stimulation with therapy provides a novel approach for clinical Piezo1-targeted treatment. Although mechanical stimulation has shown good effects in some biomechanics-related diseases, issues like individual patient differences and the personalized adjustment of mechanical stimulation need to be addressed. Effectively implementing mechanical stimulation in clinical settings to achieve optimal therapeutic effects remains an ongoing challenge.

### 5.2 Challenges and prospects in clinical translation

Despite the promising prospects of Piezo1-targeted therapies in basic research, there are several challenges in clinical translation, particularly concerning therapeutic specificity and safety. Small molecule drugs, such as Yoda1 and GsMTx4, although showing good efficacy in preclinical studies, still present important issues regarding specificity and potential side effects. For instance, Yoda1 might cause excessive Ca^2+^ influx, leading to cell damage, so more precise dosage control and clinical monitoring are required during its application (Xu et al., 2023; Gong et al., 2025; Wang C. et al., 2025; Wang W. et al., 2025). While GsMTx4 has protective effects as an antagonist, its pharmacokinetics and administration methods still need to be further researched and optimized to ensure its clinical effectiveness.

Gene therapy and mechanical stimulation strategies also face technical hurdles in clinical translation. Safety and ethical issues surrounding gene therapy must be properly addressed, and numerous technical details need to be refined regarding how to efficiently and safely deliver these therapies to patients. Mechanical stimulation, as an innovative approach, has shown potential in certain areas, but how to standardize it and adapt it to the individualized needs of different patients remains a key focus for future research.

## 6 Major challenges in clinical translation of Piezo1

### 6.1 Targeting specificity issues

As a mechanosensitive cation channel, Piezo1 is widely expressed in various tissues and cell types, and its targeting specificity remains a key challenge in clinical translation. Due to its distribution across different cell types and tissues, targeting Piezo1 may lead to off-target effects, potentially interfering with the function of normal tissues. For example, during the treatment of inflammation-related diseases, the activation of Piezo1 may not only regulate immune cell functions but also affect non-immune cells, leading to undesirable side effects (Fotiou et al., 2015; Andolfo et al., 2019; Brewer et al., 2023; Cheng et al., 2025). Additionally, in tumor cells, Piezo1 expression is often significantly upregulated and associated with tumor malignancy progression. This suggests that interventions targeting Piezo1 in cancer therapy may simultaneously impact both the TME and the surrounding normal tissues (Jiang et al., 2022; Qu and Zhang, 2025; Zhu Z. et al., 2022). Therefore, achieving high specificity targeting of Piezo1 remains a critical challenge that needs to be addressed.

To tackle this issue, researchers are exploring various tissue-specific delivery strategies. Among these, drug delivery systems based on nanocarriers are considered promising, as they can selectively accumulate Piezo1 agonists or antagonists in specific cell types. However, this technology still faces several limitations, including insufficient drug stability, limited *in vivo* delivery efficiency, and biocompatibility issues (Guan et al., 2024; Wang et al., 2025). For example, in chronic inflammation models, Piezo1 activation can enhance macrophage phagocytosis by regulating Ca^2+^ signaling. Still, if the drug is not effectively delivered to the target cells, achieving the desired therapeutic effect may be difficult (Luo S. et al., 2023; Wenqiang et al., 2024; Liu H. et al., 2022).

Current solutions have not yet fully met clinical needs. Some small molecule agonists (such as Yoda1) have shown strong bioactivity in experimental studies, but they are often limited by poor pharmacokinetics *in vivo* applications, affecting their efficacy and stability (Jäntti et al., 2022; Wang W. et al., 2025; Zhang ZH. et al., 2025). Furthermore, most of the current candidate drugs lack high specificity for particular cell types, potentially causing systemic side effects, which further limits their clinical application potential. Therefore, future research in Piezo1-targeted therapies needs to continuously explore new delivery systems and more precise molecular targeting strategies to enhance both efficacy and safety. The targeting specificity of Piezo1 remains one of the core challenges in current clinical translation research. Future studies should focus on overcoming existing technical bottlenecks and developing more accurate and safe intervention methods to enable effective targeting of Piezo1 and advance its practical application in clinical treatments.

### 6.2 Complexity of signal regulation

In the body, the Ca^2+^ signaling induced by mechanical stimulation through the Piezo1 channel is a highly complex and nonlinear process. As a typical mechanosensitive cation channel, Piezo1 plays a central role in the perception and response of cells to mechanical forces (Saotome et al., 2018; Geng et al., 2021; Luo S. et al., 2023). Studies have shown that activation of Piezo1 can quickly lead to a transient increase in intracellular Ca^2+^ concentration; however, the signal is not a simple linear response but may exhibit time-dependent and biphasic patterns. For example, brief mechanical stimulation can induce a transient influx of Ca^2+^, while under repeated or sustained stimulation, the persistence and intensity of the Ca^2+^ signal may undergo significant changes. This dynamic regulation is of considerable importance under both physiological and pathological conditions (Li W. et al., 2025; Pan et al., 2022; Szabó et al., 2022; Peussa et al., 2023). Therefore, Piezo1-mediated signaling reflects not only the channel’s own dynamic properties but is also influenced by the cellular microenvironment and systemic regulation.

Environmental factors play a significant role in the regulation of Piezo1 signaling. The stiffness of the ECM, mechanical coupling between cells, and structural characteristics of surrounding tissues all significantly impact the activity of Piezo1 (Guan et al., 2024; Baghdadi et al., 2024; Lai et al., 2022). For example, increased matrix stiffness can regulate cellular Ca^2+^ signaling through Piezo1, thereby affecting cell proliferation, migration, and differentiation (Li W. et al., 2025; Lopez-Cavestany et al., 2023; Ma et al., 2025). In the TME, mechanical properties and structural changes in the tumor tissue further enhance Piezo1 activity, promoting tumor cell invasion and distant metastasis (Zhang T. et al., 2025; Li YM. et al., 2022; Ye X. et al., 2022). Moreover, Piezo1 does not act alone; its activity often interacts with various biochemical signaling pathways, such as growth factors, cytokines, and metabolic signals, forming a highly complex signaling network (Chi et al., 2022; Velasco-Estevez et al., 2020; Cai et al., 2023). This interaction makes Piezo1’s role in cell fate determination more multi-dimensional.

From a technical perspective, dynamic regulation and precise detection of Piezo1-mediated signals still face challenges (Lei M. et al., 2024; Li Y. et al., 2024; Lee et al., 2024). How to precisely control the intensity and duration of Piezo1 activation in both time and space, to elucidate its specific contribution to cell behavior, remains a significant challenge. Current studies rely on chemical agonists (Evans et al., 2018; Sun et al., 2022) (e.g., Yoda1) or physical stimuli (Jiang Z. et al., 2024; Liu Y. et al., 2025; Zhang F. et al., 2023) (e.g., ultrasound or stretching loads), but these methods have limitations in selectivity, controllability, and reproducibility. On the other hand, intracellular Ca^2+^ signaling is highly transient and dynamic, and traditional Ca^2+^ imaging methods face limitations in capturing weak or rapid Ca^2+^ signals. Recently, advancements in high-sensitivity Ca^2+^ fluorescence probes, novel genetically encoded Ca^2+^ sensors, and super-resolution imaging technologies have provided new possibilities for real-time monitoring of Piezo1 activity and its downstream signals (Lüchtefeld et al., 2024; Wang et al., 2025c; Tao et al., 2019; Bertaccini et al., 2025; Yang J. et al., 2025). Piezo1-mediated signaling regulation exhibits complex nonlinear characteristics, influenced by various biomechanical and biochemical environmental factors, and significant challenges also exist at the experimental and technical levels. Future research needs to build on an in-depth understanding of its signaling mechanisms, integrating multi-scale imaging technologies, tissue-specific intervention strategies, and computational modeling to develop more precise and controllable regulatory methods. This aims to efficiently and safely exploit Piezo1-related signaling pathways in clinical translation.

### 6.3 Need for personalized treatment

In modern medicine, personalized treatment has become a critical trend for enhancing efficacy and reducing side effects, particularly in the field of tumor immunotherapy (Blass and Ott, 2021; Wang M. et al., 2021; He et al., 2022). As a key mechanosensitive ion channel, Piezo1’s genetic polymorphisms and functional differences are gradually receiving attention. Studies have shown that variations in the Piezo1 gene may alter the cell’s mechanosensation and Ca^2+^ signaling regulation, thereby affecting immune cell functions and the response capacity of the TME. For instance, activation of Piezo1 can induce Ca^2+^ influx, enhancing dendritic cell (DC) activation and antigen presentation, which boosts the body’s anti-tumor immune response (Bonner et al., 2025; Yu et al., 2024a; Liu C. et al., 2023). In recent studies, the Cell@CaP vaccine, through specific activation of the Piezo1 channel, has effectively enhanced DC function and demonstrated promising potential in the design of personalized cancer vaccines (Gu et al., 2025).

However, significant genetic polymorphism differences exist among patients, particularly in functional variants of the Piezo1 gene, leading to considerable variability in individual responses to the same treatment regimen (Page et al., 2021; Henkel et al., 2023; Zhang DD. et al., 2024). This not only potentially reduces treatment efficacy for some patients but also introduces new challenges in the precise design of personalized treatment. Therefore, in-depth analysis of Piezo1’s functional differences in different populations and individuals is of great value for optimizing precision medicine strategies. Future clinical research should incorporate the consideration of genetic polymorphisms in trial designs to develop more personalized treatment plans.

In addition to genetic backgrounds, differences in patients’ mechanical microenvironments are also a key factor contributing to the heterogeneity of therapeutic efficacy. Tumors in different patients exhibit significant variations in tissue stiffness (Chakraborty et al., 2021; Budde et al., 2025), ECM composition (Zhang ZM. et al., 2023; Zhao et al., 2020), and stress distribution (Lai et al., 2022; Mylvaganam et al., 2022), all of which can directly affect Piezo1 activation and Ca^2+^ signaling, thereby altering the behavior of tumor cells and immune cells. For example, in tumors with higher stiffness, excessive activation of Piezo1 may promote immune evasion, while in softer matrix environments, immune responses may be enhanced (Jiang et al., 2022; Xie et al., 2025c; Qu and Zhang, 2025). The research on the Cell@CaP vaccine suggests that combining mechanical, chemical, and immune signaling in a multimodal strategy can significantly improve DC function, effectively preventing postoperative tumor recurrence (Gu et al., 2025). This finding further underscores the importance of integrating genetic backgrounds with microenvironmental characteristics.

However, the design of current clinical trials remains limited, often focusing too much on drug efficacy while lacking a systematic consideration of patient genetic differences, mechanical environment factors, and pathway diversity. This, to some extent, restricts the generalizability and clinical application value of the results. To address this issue, future clinical trials urgently need to incorporate a multi-factorial comprehensive analysis framework, with particular attention to the differential mechanisms of Piezo1-mediated Ca^2+^ signaling across patients. By integrating big data and artificial intelligence (AI) for auxiliary analysis, it is possible to identify individual differences among patients on a larger scale, thus achieving truly personalized treatment and precision medicine.

## 7 Artificial intelligence in Piezo1 research and therapeutic development

### 7.1 AI-enhanced mechanistic and structural studies

The complex architecture and nonlinear signaling dynamics of Piezo1 present significant challenges for conventional research methods. AI particularly deep learning, offers powerful new approaches within structural and systems biology frameworks to address these issues. AI-based structure prediction tools, such as AlphaFold2, have enabled high-resolution modeling of Piezo1’s trimeric, propeller-like structure, providing crucial insights into its mechanogating mechanism. When combined with MD simulations, AI can effectively model tension-induced conformational transitions between curved and flattened states, clarifying how mechanical forces are transduced into gating events. These approaches also facilitate the identification of key residues in the pore region and lipid-interaction sites, supporting the design of state-specific modulators. Furthermore, machine learning algorithms integrate multi-omics data—genomic, transcriptomic, proteomic, and metabolomic—to reconstruct Piezo1-centered regulatory networks across physiological and pathological conditions. AI assists in detecting functional genetic variants, predicting novel signaling nodes, and identifying robust biomarkers associated with disease severity or treatment response. Such integrated analyses not only deepen the mechanistic understanding of Piezo1 but also contribute to improved patient stratification.

### 7.2 AI-guided drug discovery and optimization

AI is markedly accelerating the development of Piezo1-targeting therapeutics by facilitating rapid and intelligent screening and design of novel compounds. Through structure-based molecular docking, AI platforms are able to efficiently screen ultra-large virtual chemical libraries against Piezo1 structural models (Blass and Ott, 2021). Machine learning approaches trained on known active compounds, such as Yoda1 and GsMTx4, demonstrate strong capability in predicting binding affinity and selectivity. This enables prioritization of high-potency agonists or antagonists, substantially reducing the experimental workload. Moreover, generative AI models—including generative adversarial networks and variational autoencoders—are being employed to design novel chemical entities with optimized target engagement and improved drug-like properties, such as solubility and metabolic stability. Complementing these efforts, natural language processing techniques systematically mine scientific literature and specialized databases to construct detailed knowledge graphs. These graphs reveal previously unexplored functional links between Piezo1 and other pathways, thereby supporting the rational design of multi-target therapeutic strategies.

### 7.3 AI-enabled therapeutic personalization and delivery

AI plays a pivotal role in advancing precision medicine for Piezo1-related disorders by enabling sophisticated patient stratification and intelligent drug delivery platforms. AI models integrate multimodal data—including clinical, imaging, and molecular profiles—to predict disease trajectories and therapeutic responses. For instance, AI-driven analysis of histopathological images can infer Piezo1 activation status and tissue-level biomechanics, thereby informing intervention strategies and enhancing clinical trial design. Furthermore, AI contributes significantly to the development of precision nanomedicine by optimizing the physicochemical properties of nanocarriers—such as size, surface charge, and ligand chemistry—to improve biodistribution and achieve tissue-specific drug accumulation. Beyond targeting, reinforcement learning algorithms offer a foundation for adaptive dosing systems capable of dynamically adjusting drug administration in response to real-time physiological feedback. Such closed-loop therapeutic approaches hold particular promise for managing chronic Piezo1-related conditions, ultimately improving treatment efficacy and safety.

### 7.4 Challenges and future perspectives

Several interconnected challenges must be addressed to fully realize the potential of AI in Piezo1 research and clinical translation. A primary obstacle is the inconsistent data quality and lack of standardization across experimental studies, which significantly hinders the development of robust and generalizable AI models (Cordeil and Jallades, 2024). Furthermore, the “black box” nature of many complex AI algorithms creates barriers to clinical trust and regulatory approval, necessitating the advancement of explainable AI frameworks to enhance transparency and interpretability. Additionally, critical ethical and regulatory considerations—such as algorithmic bias, data privacy, and accountability in AI-assisted healthcare—demand careful oversight to ensure equitable and secure implementation. Overcoming these challenges will be essential for leveraging AI to advance Piezo1-targeted therapies and personalized treatment strategies.

## 8 Conclusion

Piezo1 has redefined the landscape of mechanotransduction by linking mechanical forces to Ca^2+^-dependent signaling. Its structural distinctiveness and spatiotemporal control of Ca^2+^ flux place it at the crossroads of physiology and disease. While remarkable progress has illuminated its gating mechanisms and downstream pathways, clinical translation is still nascent. Key hurdles include achieving tissue-specific modulation, minimizing off-target actions, and deciphering nonlinear Ca^2+^ dynamics in complex microenvironments. Nonetheless, selective modulators, genome editing, and nanotechnology provide unprecedented avenues for therapeutic intervention. Looking forward, multidisciplinary integration of structural biology, mechanobiology, multi-omics, and AI will be pivotal for precision regulation of Piezo1. Ultimately, Piezo1 represents both a mechanistic keystone and a promising therapeutic target, with the potential to transform clinical strategies across diverse diseases.

## Glossary

AD: Alzheimer’s Disease
AI: Artificial Intelligence
ALI: Acute Lung Injury
ARBs: Angiotensin II Receptor Blockers
ATP: Adenosine Triphosphate
BBB: Blood-Brain Barrier
**Ca** ^**2+**^: Calcium Ion
CaM: Calmodulin
CaMKII: Ca^2+^/Calmodulin-Dependent Kinase II
CICR: Calcium-Induced Calcium Release
CNS: Central Nervous System
Cryo-EM: Cryo-Electron Microscopy
CTCs: Circulating Tumor Cells
DC: Dendritic Cell
ECM: Extracellular Matrix
ER: Endoplasmic Reticulum
ERK: Extracellular Signal-Regulated Kin
FK: Fungal Keratitis
FRET: Fluorescence Resonance Energy Transfe
GANs: Generative Adversarial Networks
GLP-1: Glucagon-Like Peptide-1
GsMTx4: Grammostola spatulata Mechanotoxin 4
HSCs: Hepatic Stellate Cells
IL-6: Interleukin-6
IRI: Ischemia-Reperfusion Injury
MD: Molecular Dynamics
NFAT: Nuclear Factor of Activated T-cells
NO: Nitric Oxide
OA: Osteoarthritis
OAB: Overactive Bladder
PD: Parkinson’s Disease
PI3K/Akt: Phosphoinositide 3-Kinase/Protein Kinase B
RANKL: Receptor Activator of Nuclear Factor Kappa-B Ligand
RL: Reinforcement Learning
SERCA: Sarco/Endoplasmic Reticulum Ca^2+^-ATPase
STOML3: Stomatin-Like Protein 3
TME: Tumor Microenvironment
VSMCs: Vascular Smooth Muscle Cells
YAP: Yes-Associated Protein

## References

[B1] Abbonante V. Karkempetzaki A. I. Leon C. Krishnan A. Huang N. Di Buduo C. A. (2024). Newly identified roles for PIEZO1 mechanosensor in controlling normal megakaryocyte development and in primary myelofibrosis. Am. J. Hematol. 99 (3), 336–349. 10.1002/ajh.27184 38165047 PMC10922533

[B2] Abiff M. Alshebremi M. Bonner M. Myers J. T. Kim B. G. Tomchuck S. L. (2023). Piezo1 facilitates optimal T cell activation during tumor challenge. Oncoimmunology 12 (1), 2281179. 10.1080/2162402X.2023.2281179 38126029 PMC10732680

[B3] Albarrán-Juárez J. Iring A. Wang S. Joseph S. Grimm M. Strilic B. (2018). Piezo1 and G(q)/G(11) promote endothelial inflammation depending on flow pattern and integrin activation. J. Exp. Med. 215 (10), 2655–2672. 10.1084/jem.20180483 30194266 PMC6170174

[B4] Amado N. G. Nosyreva E. D. Thompson D. Egeland T. J. Ogujiofor O. W. Yang M. (2024). PIEZO1 loss-of-function compound heterozygous mutations in the rare congenital human disorder prune belly syndrome. Nat. Commun. 15 (1), 339. 10.1038/s41467-023-44594-0 38184690 PMC10771463

[B5] Andolfo I. Alper S. L. De Franceschi L. Auriemma C. Russo R. De Falco L. (2013). Multiple clinical forms of dehydrated hereditary stomatocytosis arise from mutations in PIEZO1. Blood 121 (19), 3925–S12. 10.1182/blood-2013-02-482489 23479567

[B6] Andolfo I. De Rosa G. Errichiello E. Manna F. Rosato B. E. Gambale A. (2019). PIEZO1 hypomorphic variants in congenital lymphatic dysplasia cause shape and hydration alterations of red blood cells. Front. Physiol. 10, 258. 10.3389/fphys.2019.00258 30930797 PMC6428731

[B7] Aranda L. C. Ribeiro I. C. Freitas T. O. Degani-Costa L. H. Dias D. S. DE Angelis K. (2023). Enhanced respiratory frequency response to lower limb mechanoreceptors activation in patients with chronic obstructive pulmonary disease. Med. Sci. Sports Exerc 55 (3), 418–429. 10.1249/MSS.0000000000003065 36730960

[B8] Arnold P. K. Jackson B. T. Paras K. I. Brunner J. S. Hart M. L. Newsom O. J. (2022). A non-canonical tricarboxylic acid cycle underlies cellular identity. Nature 603 (7901), 477–481. 10.1038/s41586-022-04475-w 35264789 PMC8934290

[B9] Atcha H. Jairaman A. Holt J. R. Meli V. S. Nagalla R. R. Veerasubramanian P. K. (2021). Mechanically activated ion channel Piezo1 modulates macrophage polarization and stiffness sensing. Nat. Commun. 12 (1), 3256. 10.1038/s41467-021-23482-5 34059671 PMC8167181

[B10] Baghdadi M. B. Houtekamer R. M. Perrin L. Rao-Bhatia A. Whelen M. Decker L. (2024). PIEZO-dependent mechanosensing is essential for intestinal stem cell fate decision and maintenance. Science 386 (6725), eadj7615. 10.1126/science.adj7615 39607940

[B11] Baratchi S. Danish H. Chheang C. Zhou Y. Huang A. Lai A. (2024). Piezo1 expression in neutrophils regulates shear-induced NETosis. Nat. Commun. 15 (1), 7023. 10.1038/s41467-024-51211-1 39174529 PMC11341855

[B12] Bartoli F. Debant M. Chuntharpursat-Bon E. Evans E. L. Musialowski K. E. Parsonage G. (2022). Endothelial Piezo1 sustains muscle capillary density and contributes to physical activity. J. Clin. Invest 132 (5), e141775. 10.1172/JCI141775 35025768 PMC8884896

[B13] Bavi N. Richardson J. Heu C. Martinac B. Poole K. (2019). PIEZO1-Mediated currents are modulated by substrate mechanics. ACS Nano 13 (11), 13545–13559. 10.1021/acsnano.9b07499 31689081

[B14] Beech D. J. Kalli A. C. (2019). Force sensing by piezo channels in cardiovascular health and disease. Arterioscler. Thromb. Vasc. Biol. 39 (11), 2228–2239. 10.1161/ATVBAHA.119.313348 31533470 PMC6818984

[B15] Bertaccini G. A. Casanellas I. Evans E. L. Nourse J. L. Dickinson G. D. Liu G. (2025). Visualizing PIEZO1 localization and activity in hiPSC-derived single cells and organoids with HaloTag technology. Nat. Commun. 16 (1), 5556. 10.1038/s41467-025-59150-1 40593468 PMC12217361

[B16] Blass E. Ott P. A. (2021). Advances in the development of personalized neoantigen-based therapeutic cancer vaccines. Nat. Rev. Clin. Oncol. 18 (4), 215–229. 10.1038/s41571-020-00460-2 33473220 PMC7816749

[B17] Bonner M. Askew D. Sathish K. V. Tomchuck S. L. Eid S. Abiff M. (2025). Piezo1 deletion enhances cross-priming of CD8+ T cells by tumor-infiltrating CD11b+ dendritic cells. J. Immunother. Cancer 13 (6), e011815. 10.1136/jitc-2025-011815 40550569 PMC12186042

[B18] Brandt J. P. Smith C. J. (2023). Piezo1-mediated spontaneous calcium transients in satellite glia impact dorsal root ganglia development. PLoS Biol. 21 (9), e3002319. 10.1371/journal.pbio.3002319 37747915 PMC10564127

[B19] Brewer C. J. Makhamreh M. M. Shivashankar K. McLaren R. Toro M. Berger S. I. (2023). PIEZO1 is the most common monogenic etiology of non-immune hydrops fetalis detected by prenatal exome sequencing. Prenat. Diagn 43 (12), 1556–1566. 10.1002/pd.6451 37902181

[B20] Brylka L. J. Alimy A. R. Tschaffon-Müller M. Jiang S. Ballhause T. M. Baranowsky A. (2024). Piezo1 expression in chondrocytes controls endochondral ossification and osteoarthritis development. Bone Res. 12 (1), 12. 10.1038/s41413-024-00315-x 38395992 PMC10891122

[B21] Bryniarska-Kubiak N. Kubiak A. Basta-Kaim A. (2023). Mechanotransductive receptor Piezo1 as a promising target in the treatment of neurological diseases. Curr. Neuropharmacol. 21 (10), 2030–2035. 10.2174/1570159X20666220927103454 36173070 PMC10556366

[B22] Budde I. Schlichting A. Ing D. Schimmelpfennig S. Kuntze A. Fels B. (2025). Piezo1-induced durotaxis of pancreatic stellate cells depends on TRPC1 and TRPV4 channels. J. Cell Sci. 138 (8), jcs263846. 10.1242/jcs.263846 40019468 PMC12136172

[B23] Buyan A. Cox C. D. Barnoud J. Li J. Chan H. S. M. Martinac B. (2020). Piezo1 forms specific, functionally important interactions with phosphoinositides and cholesterol. Biophys. J. 119 (8), 1683–1697. 10.1016/j.bpj.2020.07.043 32949489 PMC7642233

[B24] Byun K. A. Seo S. B. Oh S. Jang J. W. Son K. H. (2024). Poly-D,L-Lactic acid fillers increase subcutaneous adipose tissue volume by promoting adipogenesis in aged animal skin. Int. J. Mol. Sci. 25 (23), 12739. 10.3390/ijms252312739 39684448 PMC11641794

[B25] Cai S. Y. Yu D. Soroka C. J. Wang J. Boyer J. L. (2021). Hepatic NFAT signaling regulates the expression of inflammatory cytokines in cholestasis. J. Hepatol. 74 (3), 550–559. 10.1016/j.jhep.2020.09.035 33039404 PMC7897288

[B26] Cai G. Lu Y. Zhong W. Wang T. Li Y. Ruan X. (2023). Piezo1-mediated M2 macrophage mechanotransduction enhances bone formation through secretion and activation of transforming growth factor-β1. Cell Prolif. 56 (9), e13440. 10.1111/cpr.13440 36880296 PMC10472522

[B27] Carrisoza-Gaytan R. Kroll K. T. Hiratsuka K. Gupta N. R. Morizane R. Lewis J. A. (2023). Functional maturation of kidney organoid tubules: PIEZO1-mediated Ca(2+) signaling. Am. J. Physiol. Cell Physiol. 324 (3), C757–C768. 10.1152/ajpcell.00288.2022 36745528 PMC10027089

[B28] Catalán V. Gómez-Ambrosi J. Ramírez B. Unamuno X. Becerril S. Rodríguez A. (2024). Increased expression levels of PIEZO1 in visceral adipose tissue in obesity and type 2 diabetes are triggered by mechanical forces and are associated with inflammation. Mol. Med. 30 (1), 255. 10.1186/s10020-024-01008-1 39707172 PMC11660983

[B29] Caulier A. Jankovsky N. Demont Y. Ouled-Haddou H. Demagny J. Guitton C. (2020). PIEZO1 activation delays erythroid differentiation of normal and hereditary xerocytosis-derived human progenitor cells. Haematologica 105 (3), 610–622. 10.3324/haematol.2019.218503 31413092 PMC7049340

[B30] Chakrabarty R. P. Chandel N. S. (2021). Mitochondria as signaling organelles control mammalian stem cell fate. Cell Stem Cell 28 (3), 394–408. 10.1016/j.stem.2021.02.011 33667360 PMC7944920

[B31] Chakraborty M. Chu K. Shrestha A. Revelo X. S. Zhang X. Gold M. J. (2021). Mechanical stiffness controls dendritic cell metabolism and function. Cell Rep. 34 (2), 108609. 10.1016/j.celrep.2020.108609 33440149

[B32] Chang J. E. Buechler M. B. Gressier E. Turley S. J. Carroll M. C. (2019). Mechanosensing by Peyer's patch stroma regulates lymphocyte migration and mucosal antibody responses. Nat. Immunol. 20 (11), 1506–1516. 10.1038/s41590-019-0505-z 31611698 PMC7015178

[B33] Chen X. Wanggou S. Bodalia A. Zhu M. Dong W. Fan J. J. (2018). A feedforward mechanism mediated by mechanosensitive ion channel PIEZO1 and tissue mechanics promotes glioma aggression. Neuron 100 (4), 799–815.e7. 10.1016/j.neuron.2018.09.046 30344046

[B34] Chen L. Yan Y. Kong F. Wang J. Zeng J. Fang Z. (2022a). Contribution of oxidative stress induced by sonodynamic therapy to the calcium homeostasis imbalance enhances macrophage infiltration in glioma cells. Cancers (Basel) 14 (8), 2036. 10.3390/cancers14082036 35454942 PMC9027216

[B35] Chen J. Rodriguez M. Miao J. Liao J. Jain P. P. Zhao M. (2022b). Mechanosensitive channel Piezo1 is required for pulmonary artery smooth muscle cell proliferation. Am. J. Physiol. Lung Cell Mol. Physiol. 322 (5), L737–L760. 10.1152/ajplung.00447.2021 35318857 PMC9076422

[B36] Chen J. Miao J. Zhou D. Liao J. Wang Z. Lin Z. (2022c). Upregulation of mechanosensitive channel Piezo1 involved in high shear stress-induced pulmonary hypertension. Thromb. Res. 218, 52–63. 10.1016/j.thromres.2022.08.006 35988445

[B37] Chen G. Gao X. Chen J. Peng L. Chen S. Tang C. (2023a). Actomyosin activity and Piezo1 activity synergistically drive urinary system fibroblast activation. Adv. Sci. (Weinh) 10 (33), e2303369. 10.1002/advs.202303369 37867255 PMC10667826

[B38] Chen S. Li Z. Chen D. Cui H. Wang J. (2023b). Piezo1-mediated mechanotransduction promotes entheseal pathological new bone formation in ankylosing spondylitis. Ann. Rheum. Dis. 82 (4), 533–545. 10.1136/ard-2022-223428 36543525

[B39] Chen B. Liu X. Yu P. Xie F. Kwan J. S. H. Chan W. N. (2023c). H. Pylori-induced NF-κB-PIEZO1-YAP1-CTGF axis drives gastric cancer progression and cancer-associated fibroblast-mediated tumour microenvironment remodelling. Clin. Transl. Med. 13 (11), e1481. 10.1002/ctm2.1481 37983931 PMC10659770

[B40] Chen D. Tang Y. Lapinski P. E. Wiggins D. Sevick E. M. Davis M. J. (2024a). EPHB4-RASA1 inhibition of PIEZO1 ras activation drives lymphatic valvulogenesis. Circ. Res. 135 (11), 1048–1066. 10.1161/CIRCRESAHA.124.325383 39421925 PMC11560524

[B41] Chen F. Zhang Z. Wang W. Liu C. Huang Z. Yu C. (2024b). Omega-3 fatty acids protect cartilage from acute injurie by reducing the mechanical sensitivity of chondrocytes. J. Orthop. Surg. Res. 19 (1), 591. 10.1186/s13018-024-05081-4 39342268 PMC11437636

[B42] Cheng C. W. Earle S. L. Povstyan O. V. Randall C. Smith K. A. Debant M. (2025). PIEZO1 variant implications for biological understanding and human health. Open Biol. 15 (7), 240345. 10.1098/rsob.240345 40628291 PMC12308237

[B43] Cheung H. Zou J. Tantiwong C. Fernandez D. I. Huang J. Ahrends R. (2023). High-throughput assessment identifying major platelet Ca(2+) entry pathways via tyrosine kinase-linked and G protein-coupled receptors. Cell Calcium 112, 102738. 10.1016/j.ceca.2023.102738 37060673

[B44] Chi S. Cui Y. Wang H. Jiang J. Zhang T. Sun S. (2022). Astrocytic Piezo1-mediated mechanotransduction determines adult neurogenesis and cognitive functions. Neuron 110 (18), 2984–2999.e8. 10.1016/j.neuron.2022.07.010 35963237

[B45] Cho Y. S. Mah W. Youn D. H. Kim Y. S. Ko H. G. Bae J. Y. (2023). Increase of glutamate in satellite glial cells of the trigeminal ganglion in a rat model of craniofacial neuropathic pain. Front. Neuroanat. 17, 1302373. 10.3389/fnana.2023.1302373 38164516 PMC10758013

[B46] Choi D. Park E. Yu R. P. Cooper M. N. Choi J. (2022). Piezo1-Regulated mechanotransduction controls flow-activated lymphatic expansion. Circ. Res. 131 (2), e2–e21. 10.1161/CIRCRESAHA.121.320565 35701867 PMC9308715

[B47] Choi S. H. Shin J. Park C. Lee J. U. Lee J. Ambo Y. (2024). *In vivo* magnetogenetics for cell-type-specific targeting and modulation of brain circuits. Nat. Nanotechnol. 19 (9), 1333–1343. 10.1038/s41565-024-01694-2 38956320

[B48] Chu F. Tan R. Wang X. Zhou X. Ma R. Ma X. (2023). Transcranial magneto-acoustic stimulation attenuates synaptic plasticity impairment through the activation of Piezo1 in alzheimer's disease mouse model. Res. (Wash D C) 6, 0130. 10.34133/research.0130 37223482 PMC10202414

[B49] ChuT.WangY.WangS.LiJ.LiZ.WeiZ. (2025). Kaempferol regulating macrophage foaming and atherosclerosis through Piezo1-mediated MAPK/NF-κB and Nrf2/HO-1 signaling pathway. J. Adv. Res. 75, 635–650. 10.1016/j.jare.2024.11.016 39561922

[B50] Clapham D. E. (2007). Calcium signaling. Cell 131 (6), 1047–1058. 10.1016/j.cell.2007.11.028 18083096

[B51] Contreras G. A. Rendon C. J. Shadowens A. Chirivi M. Salcedo-Tacuma D. Lauver D. A. (2025). Perivascular adipocytes' adipogenesis is defined by their anatomical location in the descending thoracic aorta. Cells 14 (8), 579. 10.3390/cells14080579 40277904 PMC12026431

[B52] Cordeil S. Jallades L. (2024). Polycythemia revealing PIEZO1 hereditary xerocytosis. Blood 144 (1), 123. 10.1182/blood.2024024199 38963666

[B53] Coste B. Mathur J. Schmidt M. Earley T. J. Ranade S. Petrus M. J. (2010). Piezo1 and Piezo2 are essential components of distinct mechanically activated cation channels. Science 330 (6000), 55–60. 10.1126/science.1193270 20813920 PMC3062430

[B54] Cox C. D. Gottlieb P. A. (2019). Amphipathic molecules modulate PIEZO1 activity. Biochem. Soc. Trans. 47 (6), 1833–1842. 10.1042/BST20190372 31754715

[B55] Cudmore R. H. Santana L. F. (2022). Piezo1 tunes blood flow in the central nervous system. Circ. Res. 130 (10), 1547–1549. 10.1161/CIRCRESAHA.122.321144 35549371 PMC9180419

[B56] Cui C. Xu Y. Xiong X. Aryal U. K. Chen A. Chien S. (2025). Electrical stimulation generates induced tumor-suppressing cells, offering a potential option for combatting breast cancer and bone metastasis. Int. J. Mol. Sci. 26 (3), 1030. 10.3390/ijms26031030 39940798 PMC11817334

[B57] Dai Y. Xie Q. Zhang Y. Sun Y. Zhu S. Wang C. (2024). Neoteric semiembedded β-Tricalcium phosphate promotes osteogenic differentiation of mesenchymal stem cells under cyclic Stretch. ACS Appl. Mater Interfaces 16 (7), 8289–8300. 10.1021/acsami.3c15090 38329794

[B58] Dalghi M. G. Clayton D. R. Ruiz W. G. Al-Bataineh M. M. Satlin L. M. Kleyman T. R. (2019). Expression and distribution of PIEZO1 in the mouse urinary tract. Am. J. Physiol. Ren. Physiol. 317 (2), F303–F321. 10.1152/ajprenal.00214.2019 31166705 PMC6732449

[B59] Della Pietra A. Mikhailov N. Giniatullin R. (2023). FM1-43 dye memorizes Piezo1 activation in the trigeminal nociceptive system implicated in migraine pain. Int. J. Mol. Sci. 24 (2), 1688. 10.3390/ijms24021688 36675204 PMC9861983

[B60] Deng J. Xie Y. Shen J. Gao Q. He J. Ma H. (2022). Photocurable hydrogel substrate-better potential substitute on bone-marrow-derived dendritic cells culturing. Mater. (Basel) 15 (9), 3322. 10.3390/ma15093322 35591655 PMC9104740

[B61] Dombroski J. A. Hope J. M. Sarna N. S. King M. R. (2021). Channeling the force: piezo1 mechanotransduction in cancer metastasis. Cells 10 (11), 2815. 10.3390/cells10112815 34831037 PMC8616475

[B62] Douguet D. Honoré E. (2019). Mammalian mechanoelectrical transduction: structure and function of force-gated ion channels. Cell 179 (2), 340–354. 10.1016/j.cell.2019.08.049 31585078

[B63] Douguet D. Patel A. Xu A. Vanhoutte P. M. Honoré E. (2019). Piezo ion channels in cardiovascular mechanobiology. Trends Pharmacol. Sci. 40 (12), 956–970. 10.1016/j.tips.2019.10.002 31704174

[B64] Driskill J. H. Pan D. (2023). Control of stem cell renewal and fate by YAP and TAZ. Nat. Rev. Mol. Cell Biol. 24 (12), 895–911. 10.1038/s41580-023-00644-5 37626124

[B65] Duan X. Liu R. Xi Y. Tian Z. (2025). The mechanisms of exercise improving cardiovascular function by stimulating Piezo1 and TRP ion channels: a systemic review. Mol. Cell Biochem. 480 (1), 119–137. 10.1007/s11010-024-05000-5 38625513

[B66] Dubin A. E. Murthy S. Lewis A. H. Brosse L. Cahalan S. M. Grandl J. (2017). Endogenous Piezo1 can confound mechanically activated channel identification and characterization. Neuron 94 (2), 266–270.e3. 10.1016/j.neuron.2017.03.039 28426961 PMC5448662

[B67] Eisenhoffer G. T. Loftus P. D. Yoshigi M. Otsuna H. Chien C. B. Morcos P. A. (2012). Crowding induces live cell extrusion to maintain homeostatic cell numbers in epithelia. Nature 484 (7395), 546–549. 10.1038/nature10999 22504183 PMC4593481

[B68] Eisner D. A. Caldwell J. L. Kistamás K. Trafford A. W. (2017). Calcium and excitation-contraction coupling in the heart. Circ. Res. 121 (2), 181–195. 10.1161/CIRCRESAHA.117.310230 28684623 PMC5497788

[B69] Endo M. (2009). Calcium-induced calcium release in skeletal muscle. Physiol. Rev. 89 (4), 1153–1176. 10.1152/physrev.00040.2008 19789379

[B70] Evans E. L. Cuthbertson K. Endesh N. Rode B. Blythe N. M. Hyman A. J. (2018). Yoda1 analogue (Dooku1) which antagonizes Yoda1-evoked activation of Piezo1 and aortic relaxation. Br. J. Pharmacol. 175 (10), 1744–1759. 10.1111/bph.14188 29498036 PMC5913400

[B71] Evans E. L. Povstyan O. V. De Vecchis D. Macrae F. Lichtenstein L. Futers T. S. (2020). RBCs prevent rapid PIEZO1 inactivation and expose slow deactivation as a mechanism of dehydrated hereditary stomatocytosis. Blood 136 (1), 140–144. 10.1182/blood.2019004174 32305040 PMC7381761

[B72] Ezzo M. Spindler K. Wang J. B. Lee D. Pecoraro G. Cowen J. (2024). Acute contact with profibrotic macrophages mechanically activates fibroblasts via αvβ3 integrin-mediated engagement of Piezo1. Sci. Adv. 10 (43), eadp4726. 10.1126/sciadv.adp4726 39441936 PMC11498225

[B73] Fang X. Z. Li M. Wang Y. X. Zhang P. Sun M. M. Xu J. X. (2023). Mechanosensitive ion channel Piezo1 mediates mechanical ventilation-exacerbated ARDS-associated pulmonary fibrosis. J. Adv. Res. 53, 175–186. 10.1016/j.jare.2022.12.006 36526145 PMC10658225

[B74] Fei L. Xu M. Wang H. Zhong C. Jiang S. Lichtenberger F. B. (2023). Piezo1 mediates vasodilation induced by acute hyperglycemia in mouse renal arteries and microvessels. Hypertension 80 (8), 1598–1610. 10.1161/HYPERTENSIONAHA.122.20767 37259842

[B75] Feng X. Li S. Wang S. Meng Y. Zheng S. Liu C. (2024). Piezo1 mediates the degradation of cartilage extracellular matrix in malocclusion-induced TMJOA. Oral Dis. 30 (4), 2425–2438. 10.1111/odi.14615 37184045

[B76] Fish A. Kulkarni A. (2024). Flow-induced shear stress primes NLRP3 inflammasome activation in macrophages via Piezo1. ACS Appl. Mater Interfaces 16 (4), 4505–4518. 10.1021/acsami.3c18645 38240257 PMC12965337

[B77] Fotiou E. Martin-Almedina S. Simpson M. A. Lin S. Gordon K. Brice G. (2015). Novel mutations in PIEZO1 cause an autosomal recessive generalized lymphatic dysplasia with non-immune hydrops fetalis. Nat. Commun. 6, 8085. 10.1038/ncomms9085 26333996 PMC4568316

[B78] Friedrich E. E. Hong Z. Xiong S. Zhong M. Rehman J. (2019). Endothelial cell Piezo1 mediates pressure-induced lung vascular hyperpermeability via disruption of adherens junctions. Proc. Natl. Acad. Sci. U. S. A. 116 (26), 12980–12985. 10.1073/pnas.1902165116 31186359 PMC6600969

[B79] Fu H. Yu Y. Wang S. Xu P. Sun Y. Li J. (2025). Piezo1 disrupts blood-brain barrier via CaMKII/Nrf2 in ischemic stroke. Cell Mol. Life Sci. 82 (1), 259. 10.1007/s00018-025-05804-8 40579608 PMC12204977

[B80] Gan D. Tao C. Jin X. Wu X. Yan Q. Zhong Y. (2024). Piezo1 activation accelerates osteoarthritis progression and the targeted therapy effect of artemisinin. J. Adv. Res. 62, 105–117. 10.1016/j.jare.2023.09.040 37758057 PMC11331168

[B81] Ganugula R. Arora M. Dwivedi S. Chandrashekar D. S. Varambally S. Scott E. M. (2023). Systemic anti-inflammatory therapy aided by curcumin-laden double-headed nanoparticles combined with injectable long-acting insulin in a rodent model of diabetes eye disease. ACS Nano 17 (7), 6857–6874. 10.1021/acsnano.3c00535 36951721

[B82] Gao L. Ardiel E. Nurrish S. Kaplan J. M. (2024). Voltage-induced calcium release in *Caenorhabditis elegans* body muscles. Proc. Natl. Acad. Sci. U. S. A. 121 (19), e2317753121. 10.1073/pnas.2317753121 38687794 PMC11087772

[B83] Gao L. Yang K. Zhao Y. Zhang J. Jiang S. Zhang R. (2025). Intestinal L-cell mechanoreception regulates hepatic lipid metabolism through GLP-1. Sci. Adv. 11 (22), eadv3201. 10.1126/sciadv.adv3201 40446026 PMC12124353

[B84] Garcia V. Blaquiere M. Janvier A. Cresto N. Lana C. Genin A. (2023). PIEZO1 expression at the glio-vascular unit adjusts to neuroinflammation in seizure conditions. Neurobiol. Dis. 187, 106297. 10.1016/j.nbd.2023.106297 37717661

[B85] Ge J. Li W. Zhao Q. Chen M. Zhi P. (2015). Architecture of the mammalian mechanosensitive Piezo1 channel. Nature 527 (7576), 64–69. 10.1038/nature15247 26390154

[B86] Geng J. Shi Y. Zhang J. Yang B. Wang P. Yuan W. (2021). TLR4 signalling via Piezo1 engages and enhances the macrophage mediated host response during bacterial infection. Nat. Commun. 12 (1), 3519. 10.1038/s41467-021-23683-y 34112781 PMC8192512

[B87] Glogowska E. Lezon-Geyda K. Maksimova Y. Schulz V. P. Gallagher P. G. (2015). Mutations in the gardos channel (KCNN4) are associated with hereditary xerocytosis. Blood 126 (11), 1281–1284. 10.1182/blood-2015-07-657957 26198474 PMC4566808

[B88] Glogowska E. Arhatte M. Chatelain F. C. Lesage F. Xu A. Grashoff C. (2021). Piezo1 and Piezo2 foster mechanical gating of K(2P) channels. Cell Rep. 37 (9), 110070. 10.1016/j.celrep.2021.110070 34852225

[B89] Gonçalves A. N. Moura R. S. Correia-Pinto J. Nogueira-Silva C. (2023). Intraluminal chloride regulates lung branching morphogenesis: involvement of PIEZO1/PIEZO2. Respir. Res. 24 (1), 42. 10.1186/s12931-023-02328-2 36740669 PMC9901166

[B90] Gong A. Dai J. Zhao Y. Hu H. Guan C. Yu H. (2025). Piezo1 activation protects against sepsis-induced myocardial dysfunction in a pilot study. Sci. Rep. 15 (1), 15975. 10.1038/s41598-025-00829-2 40341084 PMC12062470

[B91] Grannemann C. Pabst A. Honert A. Schieren J. Martin C. Hank S. (2023). Mechanical activation of lung epithelial cells through the ion channel Piezo1 activates the metalloproteinases ADAM10 and ADAM17 and promotes growth factor and adhesion molecule release. Biomater. Adv. 152, 213516. 10.1016/j.bioadv.2023.213516 37348330

[B92] Greenlee J. D. Liu K. Lopez-Cavestany M. King M. R. (2022). Piezo1 mechano-activation is augmented by resveratrol and differs between colorectal cancer cells of primary and metastatic origin. Molecules 27 (17), 5430. 10.3390/molecules27175430 36080197 PMC9458129

[B93] Gu Z. Li L. Xu P. Li C. Liu B. Zhu P. (2025). Multimodal regulation of dendritic cells via mineralized vaccines for postsurgical tumor relapse prevention. ACS Nano 19 (21), 19901–19917. 10.1021/acsnano.5c02846 40401399

[B94] Guan H. Wang W. Jiang Z. Zhang B. Ye Z. Zheng J. (2024). Magnetic aggregation-induced bone-targeting nanocarrier with effects of Piezo1 activation and osteogenic-angiogenic coupling for osteoporotic bone repair. Adv. Mater 36 (13), e2312081. 10.1002/adma.202312081 38102981

[B95] Gudipaty S. A. Lindblom J. Loftus P. D. Redd M. J. Edes K. Davey C. F. (2017). Mechanical stretch triggers rapid epithelial cell division through Piezo1. Nature 543 (7643), 118–121. 10.1038/nature21407 28199303 PMC5334365

[B96] Guo T. Chen G. Yang L. Deng J. Pan Y. (2024). Piezo1 inhibitor isoquercitrin rescues neural impairment mediated by NLRP3 after intracerebral hemorrhage. Exp. Neurol. 379, 114852. 10.1016/j.expneurol.2024.114852 38857751

[B97] Hao R. Tang H. Ding C. Rajbanshi B. Liu Y. Ma D. (2024). A novel Piezo1 agonist promoting mesenchymal stem cell proliferation and osteogenesis to attenuate disuse osteoporosis. Small Sci. 4 (9), 2400061. 10.1002/smsc.202400061 40212079 PMC11935128

[B98] Harraz O. F. Hashad A. M. (2025). Brain capillary ion channels: physiology and channelopathies. Physiol. (Bethesda). 10.1152/physiol.00015.2025 40748720 PMC12378794

[B99] Harraz O. F. Klug N. R. Senatore A. J. Hill-Eubanks D. C. Nelson M. T. (2022). Piezo1 is a mechanosensor channel in central nervous system capillaries. Circ. Res. 130 (10), 1531–1546. 10.1161/CIRCRESAHA.122.320827 35382561 PMC9106929

[B100] He J. Fang B. Shan S. Xie Y. Wang C. Zhang Y. (2021). Mechanical stretch promotes hypertrophic scar formation through mechanically activated cation channel Piezo1. Cell Death Dis. 12 (3), 226. 10.1038/s41419-021-03481-6 33649312 PMC7921104

[B101] He J. Xiong X. Yang H. Li D. Liu X. Li S. (2022). Defined tumor antigen-specific T cells potentiate personalized TCR-T cell therapy and prediction of immunotherapy response. Cell Res. 32 (6), 530–542. 10.1038/s41422-022-00627-9 35165422 PMC9160085

[B102] He D. Liu X. Yang W. Guan T. Wang G. (2024). The role of mechanosensitive ion channel Piezo1 in knee osteoarthritis inflammation. Channels (Austin) 18 (1), 2393088. 10.1080/19336950.2024.2393088 39169878 PMC11346567

[B103] Henkel C. Styrkársdóttir U. Thorleifsson G. Stefánsdóttir L. Björnsdóttir G. Banasik K. (2023). Genome-wide association meta-analysis of knee and hip osteoarthritis uncovers genetic differences between patients treated with joint replacement and patients without joint replacement. Ann. Rheum. Dis. 82 (3), 384–392. 10.1136/ard-2022-223199 36376028

[B104] Hill R. Z. Loud M. C. Dubin A. E. Peet B. Patapoutian A. (2022). PIEZO1 transduces mechanical itch in mice. Nature 607 (7917), 104–110. 10.1038/s41586-022-04860-5 35732741 PMC9259491

[B105] Hirabayashi Y. Kwon S. K. Paek H. Pernice W. M. Paul M. A. Lee J. (2017). ER-mitochondria tethering by PDZD8 regulates Ca(2+) dynamics in mammalian neurons. Science 358 (6363), 623–630. 10.1126/science.aan6009 29097544 PMC5818999

[B106] Hirata Y. Cai R. Volchuk A. Steinberg B. E. Saito Y. Matsuzawa A. (2023). Lipid peroxidation increases membrane tension, Piezo1 gating, and cation permeability to execute ferroptosis. Curr. Biol. 33 (7), 1282–1294.e5. 10.1016/j.cub.2023.02.060 36898371

[B107] Hong G. S. Lee B. Oh U. (2017). Evidence for mechanosensitive channel activity of tentonin 3/TMEM150C. Neuron 94 (2), 271–273.e2. 10.1016/j.neuron.2017.03.038 28426962

[B108] Hope J. M. Dombroski J. A. Pereles R. S. Lopez-Cavestany M. Greenlee J. D. Schwager S. C. (2022). Fluid shear stress enhances T cell activation through Piezo1. BMC Biol. 20 (1), 61. 10.1186/s12915-022-01266-7 35260156 PMC8904069

[B109] Hu J. Chen Q. Zhu H. Hou L. Liu W. Yang Q. (2023). Microglial Piezo1 senses Aβ fibril stiffness to restrict alzheimer's disease. Neuron 111 (1), 15–29.e8. 10.1016/j.neuron.2022.10.021 36368316

[B110] Huang J. Zhang K. Du R. Liu W. Zhang H. Tian T. (2023). The Janus-faced role of Piezo1 in cardiovascular health under mechanical stimulation. Genes Dis. 10 (5), 1956–1968. 10.1016/j.gendis.2022.08.015 37492728 PMC10363580

[B111] Huang L. Jiao Y. Xia H. Li H. Yu J. Que Y. (2024). Strontium zinc silicate simultaneously alleviates osteoporosis and sarcopenia in tail-suspended rats via Piezo1-mediated Ca(2+) signaling. J. Orthop. Transl. 48, 146–155. 10.1016/j.jot.2024.07.014 39229332 PMC11369381

[B112] Iring A. Jin Y. J. Albarrán-Juárez J. Siragusa M. Wang S. Dancs P. T. (2019). Shear stress-induced endothelial adrenomedullin signaling regulates vascular tone and blood pressure. J. Clin. Invest 129 (7), 2775–2791. 10.1172/JCI123825 31205027 PMC6597232

[B113] Ivkovic S. Major T. Mitic M. Loncarevic-Vasiljkovic N. Jovic M. Adzic M. (2022). Fatty acids as biomodulators of Piezo1 mediated glial mechanosensitivity in alzheimer's disease. Life Sci. 297, 120470. 10.1016/j.lfs.2022.120470 35283177

[B114] Jacobs B. M. Stow D. Hodgson S. Zöllner J. Samuel M. Kanoni S. (2024). Genetic architecture of routinely acquired blood tests in a British South Asian cohort. Nat. Commun. 15 (1), 8929. 10.1038/s41467-024-53091-x 39414775 PMC11484750

[B115] Jakob D. Klesen A. Allegrini B. Darkow E. Aria D. Emig R. (2021). Piezo1 and BK(Ca) channels in human atrial fibroblasts: interplay and remodelling in atrial fibrillation. J. Mol. Cell Cardiol. 158, 49–62. 10.1016/j.yjmcc.2021.05.002 33974928

[B116] Jäntti H. Sitnikova V. Ishchenko Y. Shakirzyanova A. Giudice L. Ugidos I. F. (2022). Microglial amyloid beta clearance is driven by PIEZO1 channels. J. Neuroinflammation 19 (1), 147. 10.1186/s12974-022-02486-y 35706029 PMC9199162

[B117] Jia S. Liu W. Zhang M. Wang L. Ren C. Feng C. (2024a). Insufficient mechanical loading downregulates Piezo1 in chondrocytes and impairs fracture healing through ApoE-Induced senescence. Adv. Sci. (Weinh) 11 (46), e2400502. 10.1002/advs.202400502 39418070 PMC11633519

[B118] Jia Z. Wang J. Li X. Yang Q. Han J. (2024b). Repair effect of siRNA double silencing of the novel mechanically sensitive ion channels Piezo1 and TRPV4 on an osteoarthritis rat model. Curr. Mol. Pharmacol. 17, e18761429317745. 10.2174/0118761429317745241017114020 39660528

[B119] Jiang F. Yin K. Wu K. Zhang M. Wang S. Cheng H. (2021a). The mechanosensitive Piezo1 channel mediates heart mechano-chemo transduction. Nat. Commun. 12 (1), 869. 10.1038/s41467-021-21178-4 33558521 PMC7870949

[B120] Jiang Y. Guan Y. Lan Y. Chen S. Zou S. (2021b). Mechanosensitive Piezo1 in periodontal ligament cells promotes alveolar bone remodeling during orthodontic tooth movement. Front. Physiol. 12, 767136. 10.3389/fphys.2021.767136 34880779 PMC8645976

[B121] Jiang Y. Song J. Xu Y. Liu C. Qian W. Bai T. (2021c). Piezo1 regulates intestinal epithelial function by affecting the tight junction protein claudin-1 via the ROCK pathway. Life Sci. 275, 119254. 10.1016/j.lfs.2021.119254 33636174

[B122] Jiang Y. Zhang H. Wang J. Liu Y. Luo T. Hua H. (2022). Targeting extracellular matrix stiffness and mechanotransducers to improve cancer therapy. J. Hematol. Oncol. 15 (1), 34. 10.1186/s13045-022-01252-0 35331296 PMC8943941

[B123] Jiang J. Wang F. Huang W. Sun J. Ye Y. Ou J. (2023a). Mobile mechanical signal generator for macrophage polarization. Explor. (Beijing) 3 (2), 20220147. 10.1002/EXP.20220147 37324036 PMC10190931

[B124] Jiang M. Zhang Y. X. Bu W. J. Li P. Chen J. H. Cao M. (2023b). Piezo1 channel activation stimulates ATP production through enhancing mitochondrial respiration and glycolysis in vascular endothelial cells. Br. J. Pharmacol. 180 (14), 1862–1877. 10.1111/bph.16050 36740831

[B125] Jiang Z. Chen Z. Xu Y. Li H. Li Y. Peng L. (2024a). Low-frequency ultrasound sensitive Piezo1 channels regulate keloid-related characteristics of fibroblasts. Adv. Sci. (Weinh) 11 (14), e2305489. 10.1002/advs.202305489 38311578 PMC11005750

[B126] Jiang T. Yu F. Zhou Y. Li R. Zheng M. Jiang Y. (2024b). Synergistic effect of ultrasound and reinforced electrical environment by bioinspired periosteum for enhanced osteogenesis via immunomodulation of macrophage polarization through Piezo1. Mater Today Bio 27, 101147. 10.1016/j.mtbio.2024.101147 39045313 PMC11263955

[B127] Jiang D. Zhao J. Zheng J. Zhao Y. Le M. Qin D. (2024c). LOX-mediated ECM mechanical stress induces Piezo1 activation in hypoxic-ischemic brain damage and identification of novel inhibitor of LOX. Redox Biol. 76, 103346. 10.1016/j.redox.2024.103346 39260063 PMC11414707

[B128] Johnson R. T. Solanki R. Wostear F. Ahmed S. Taylor J. C. K. Rees J. (2024). Piezo1-mediated regulation of smooth muscle cell volume in response to enhanced extracellular matrix rigidity. Br. J. Pharmacol. 181 (11), 1576–1595. 10.1111/bph.16294 38044463

[B129] Karamatic Crew V. Tilley L. A. Satchwell T. J. AlSubhi S. A. Jones B. Spring F. A. (2023). Missense mutations in PIEZO1, which encodes the Piezo1 mechanosensor protein, define Er red blood cell antigens. Blood 141 (2), 135–146. 10.1182/blood.2022016504 36122374 PMC10644042

[B130] Karkempetzaki A. I. Ravid K. (2024). Piezo1 and its function in different blood cell lineages. Cells 13 (6), 482. 10.3390/cells13060482 38534326 PMC10969519

[B131] Kiang K. M. Ahad L. Zhong X. Lu Q. R. (2024). Biomolecular condensates: hubs of Hippo-YAP/TAZ signaling in cancer. Trends Cell Biol. 34 (7), 566–577. 10.1016/j.tcb.2024.04.009 38806345

[B132] Kim S. E. Coste B. Chadha A. Cook B. Patapoutian A. (2012). The role of drosophila piezo in mechanical nociception. Nature 483 (7388), 209–212. 10.1038/nature10801 22343891 PMC3297676

[B133] Kim O. H. Choi Y. W. Park J. H. Hong S. A. Hong M. Chang I. H. (2022). Fluid shear stress facilitates prostate cancer metastasis through Piezo1-Src-YAP axis. Life Sci. 308, 120936. 10.1016/j.lfs.2022.120936 36084759

[B134] Knoepp F. Abid S. Houssaini A. Lipskaia L. Gökyildirim M. Y. Born E. (2025). Piezo1 in PASMCs: critical for hypoxia-induced pulmonary hypertension development. Circ. Res. 136 (9), 1031–1048. 10.1161/CIRCRESAHA.124.325475 40181773 PMC12036789

[B135] Knutson K. R. Whiteman S. T. Alcaino C. Mercado-Perez A. Finholm I. Serlin H. K. (2023). Intestinal enteroendocrine cells rely on ryanodine and IP(3) calcium store receptors for mechanotransduction. J. Physiol. 601 (2), 287–305. 10.1113/JP283383 36428286 PMC9840706

[B136] Konishi T. Kamiyama K. Osato T. Yoshimoto T. Aoki T. Anzai T. (2024). Increased Piezo1 expression in myofibroblasts in patients with symptomatic carotid atherosclerotic plaques undergoing carotid endarterectomy: a pilot study. Vascular 32 (5), 1063–1069. 10.1177/17085381231192380 37499697

[B137] Koo J. H. Guan K. L. (2018). Interplay between YAP/TAZ and metabolism. Cell Metab. 28 (2), 196–206. 10.1016/j.cmet.2018.07.010 30089241

[B138] Korobkin J. Balabin F. A. Yakovenko S. A. Simonenko E. Y. Sveshnikova A. N. (2021). Occurrence of calcium oscillations in human spermatozoa is based on spatial signaling enzymes distribution. Int. J. Mol. Sci. 22 (15), 8018. 10.3390/ijms22158018 34360784 PMC8347727

[B139] Krivoshein G. Tolner E. A. Maagdenberg A. Giniatullin R. A. (2022). Migraine-relevant sex-dependent activation of mouse meningeal afferents by TRPM3 agonists. J. Headache Pain 23 (1), 4. 10.1186/s10194-021-01383-8 35012445 PMC8903645

[B140] Lai A. Cox C. D. Chandra S. N. Thurgood P. Jaworowski A. Peter K. (2022). Mechanosensing by Piezo1 and its implications for physiology and various pathologies. Biol. Rev. Camb Philos. Soc. 97 (2), 604–614. 10.1111/brv.12814 34781417

[B141] Lan Y. Lu J. Zhang S. Jie C. Chen C. Xiao C. (2024). Piezo1-Mediated mechanotransduction contributes to disturbed flow-induced atherosclerotic endothelial inflammation. J. Am. Heart Assoc. 13 (21), e035558. 10.1161/JAHA.123.035558 39450718 PMC11935715

[B142] Larriva-Sahd J. Martínez-Cabrera G. Lozano-Flores C. Concha L. Varela-Echavarría A. (2023). The neurovascular unit of capillary blood vessels in the rat nervous system. A rapid-Golgi electron microscopy study. J. Comp. Neurol. 532 (2), e25559. 10.1002/cne.25559 38009706

[B143] Lee W. Leddy H. A. Chen Y. Lee S. H. Zelenski N. A. McNulty A. L. (2014). Synergy between Piezo1 and Piezo2 channels confers high-strain mechanosensitivity to articular cartilage. Proc. Natl. Acad. Sci. U. S. A. 111 (47), E5114–E5122. 10.1073/pnas.1414298111 25385580 PMC4250098

[B144] Lee W. Nims R. J. Savadipour A. Zhang Q. Leddy H. A. Liu F. (2021a). Inflammatory signaling sensitizes Piezo1 mechanotransduction in articular chondrocytes as a pathogenic feed-forward mechanism in osteoarthritis. Proc. Natl. Acad. Sci. U. S. A. 118 (13), e2001611118. 10.1073/pnas.2001611118 33758095 PMC8020656

[B145] Lee J. U. Shin W. Lim Y. Kim J. Kim W. R. Kim H. (2021b). Non-contact long-range magnetic stimulation of mechanosensitive ion channels in freely moving animals. Nat. Mater 20 (7), 1029–1036. 10.1038/s41563-020-00896-y 33510447

[B146] Lee C. S. Zhai Y. Shang R. Wong T. Mattison A. J. Cen H. H. (2022). Flow-induced secretion of endothelial heparanase regulates cardiac lipoprotein lipase and changes following diabetes. J. Am. Heart Assoc. 11 (23), e027958. 10.1161/JAHA.122.027958 36416172 PMC9851453

[B147] Lee Y. Seo S. H. Kim J. Lee J. Y. Lee J. O. (2024). Diagnostic approaches to investigate JAK2-Unmutated erythrocytosis based on a single tertiary center experience. Mol. Diagn Ther. 28 (3), 311–318. 10.1007/s40291-024-00703-3 38568469 PMC11068693

[B148] Lei M. Wang W. Zhang H. Gong J. Cai H. Wang Z. (2024a). Piezo1 regulates stiffness-dependent DRG axon regeneration via modifying cytoskeletal dynamics. Adv. Sci. (Weinh) 11 (47), e2405705. 10.1002/advs.202405705 39514408 PMC11653623

[B149] Lei L. Wen Z. Cao M. Zhang H. Ling S. K. K. Fu B. S. C. (2024b). The emerging role of Piezo1 in the musculoskeletal system and disease. Theranostics 14 (10), 3963–3983. 10.7150/thno.96959 38994033 PMC11234281

[B150] Leng S. Zhang X. Wang S. Qin J. Liu Q. Liu A. (2022). Ion channel Piezo1 activation promotes aerobic glycolysis in macrophages. Front. Immunol. 13, 976482. 10.3389/fimmu.2022.976482 36119083 PMC9479104

[B151] Leng S. Zhang X. Zhao R. Jiang N. Liu X. Li X. (2025). Mechanical activation of adipose tissue macrophages mediated by Piezo1 protects against diet-induced obesity by regulating sympathetic activity. Metabolism 168, 156262. 10.1016/j.metabol.2025.156262 40204210

[B152] Lew V. L. (2025). The calcium homeostasis of human red blood cells in health and disease: interactions of PIEZO1, the plasma membrane calcium pump, and gardos channels. Annu. Rev. Physiol. 87 (1), 257–277. 10.1146/annurev-physiol-022724-105119 39476416

[B153] Li J. Hou B. Tumova S. Muraki K. Bruns A. Ludlow M. J. (2014). Piezo1 integration of vascular architecture with physiological force. Nature 515 (7526), 279–282. 10.1038/nature13701 25119035 PMC4230887

[B154] Li F. Lo T. Y. Miles L. Wang Q. Noristani H. N. Li D. (2021). The Atr-Chek1 pathway inhibits axon regeneration in response to Piezo-dependent mechanosensation. Nat. Commun. 12 (1), 3845. 10.1038/s41467-021-24131-7 34158506 PMC8219705

[B155] Li M. Zhang X. Wang M. Wang Y. Qian J. Xing X. (2022a). Activation of Piezo1 contributes to matrix stiffness-induced angiogenesis in hepatocellular carcinoma. Cancer Commun. (Lond) 42 (11), 1162–1184. 10.1002/cac2.12364 36181398 PMC9648387

[B156] Li H. Wu B. K. Kanchwala M. Cai J. Wang L. Xing C. (2022b). YAP/TAZ drives cell proliferation and tumour growth via a polyamine-eIF5A hypusination-LSD1 axis. Nat. Cell Biol. 24 (3), 373–383. 10.1038/s41556-022-00848-5 35177822 PMC8930503

[B157] Li J. Zhang Y. Lou Z. Cui L. Yang Z. (2022c). Magnetic nanobubble mechanical stress induces the Piezo1-Ca(2+) -BMP2/Smad pathway to modulate neural stem cell fate and MRI/ultrasound dual imaging surveillance for ischemic stroke. Small 18 (23), e2201123. 10.1002/smll.202201123 35555970

[B158] Li Q. Y. Duan Y. W. Zhou Y. H. Chen S. X. Li Y. Y. Zang Y. (2022d). NLRP3-Mediated Piezo1 upregulation in ACC inhibitory parvalbumin-expressing interneurons is involved in pain processing after peripheral nerve injury. Int. J. Mol. Sci. 23 (21), 13035. 10.3390/ijms232113035 36361825 PMC9655876

[B159] Li X. Hu J. Zhao X. Li J. Chen Y. (2022e). Piezo channels in the urinary system. Exp. Mol. Med. 54 (6), 697–710. 10.1038/s12276-022-00777-1 35701561 PMC9256749

[B160] Li Y. M. Xu C. Sun B. Zhong F. J. Cao M. Yang L. Y. (2022f). Piezo1 promoted hepatocellular carcinoma progression and EMT through activating TGF-β signaling by recruiting Rab5c. Cancer Cell Int. 22 (1), 162. 10.1186/s12935-022-02574-2 35461277 PMC9035260

[B161] Li Y. Liu Z. Han X. Liang F. Zhang Q. Huang X. (2024a). Dynamics of endothelial cell generation and turnover in arteries during homeostasis and diseases. Circulation 149 (2), 135–154. 10.1161/CIRCULATIONAHA.123.064301 38084582

[B162] Li M. J. Li C. X. Li J. Y. Gong Z. C. Shao B. Zhou Y. C. (2024b). Biomechanism of abnormal stress on promoting osteoarthritis of temporomandibular joint through Piezo1 ion channel. J. Oral Rehabil. 51 (10), 1935–1946. 10.1111/joor.13777 38873703

[B163] Li B. Chen Z. Zhang Z. Liu H. Han D. Yang H. (2024c). Zuogui pill disrupt the malignant cycle in breast cancer bone metastasis through the Piezo1-Notch-1-GPX4 pathway and active molecules fishing. Phytomedicine 123, 155257. 10.1016/j.phymed.2023.155257 38103318

[B164] Li Q. Li C. Liu X. Guo Z. Li X. Zhang X. (2025a). The key role of Piezo1 channels in ferroptosis after spinal cord injury and the therapeutic potential of Piezo1 inhibitors. Prog. Biophys. Mol. Biol. 196, 132–140. 10.1016/j.pbiomolbio.2025.05.001 40339662

[B165] Li W. Zhang Z. Peng Z. Hu H. Cui X. Zhu Z. (2025b). PIEZO1-Mediated calcium signaling and podocyte injury in diabetic kidney disease. J. Am. Soc. Nephrol. 36 (7), 1310–1326. 10.1681/ASN.0000000634 39932793 PMC12187238

[B166] Liang P. Zhang Y. Wan Y. Ma S. Dong P. Lowry A. J. (2024). Deciphering and disrupting PIEZO1-TMEM16F interplay in hereditary xerocytosis. Blood 143 (4), 357–369. 10.1182/blood.2023021465 38033286 PMC10862370

[B167] Liao J. Lu W. Chen Y. Duan X. Zhang C. Luo X. (2021). Upregulation of Piezo1 (piezo type mechanosensitive ion channel component 1) enhances the intracellular free calcium in pulmonary arterial smooth muscle cells from idiopathic pulmonary arterial hypertension patients. Hypertension 77 (6), 1974–1989. 10.1161/HYPERTENSIONAHA.120.16629 33813851

[B168] Liao W. Li Y. Liu T. Deng J. Liang H. Shen F. (2024). The activation of Piezo1 channel promotes invasion and migration via the release of extracellular ATP in cervical cancer. Pathol. Res. Pract. 260, 155426. 10.1016/j.prp.2024.155426 38908334

[B169] Lim G. B. (2022). Piezo1 senses pressure overload and initiates cardiac hypertrophy. Nat. Rev. Cardiol. 19 (8), 503. 10.1038/s41569-022-00746-1 35768693

[B170] Lim X. R. Harraz O. F. (2024). Mechanosensing by vascular endothelium. Annu. Rev. Physiol. 86, 71–97. 10.1146/annurev-physiol-042022-030946 37863105 PMC10922104

[B171] Lim X. R. Abd-Alhaseeb M. M. Ippolito M. Koide M. Senatore A. J. Plante C. (2024). Endothelial Piezo1 channel mediates mechano-feedback control of brain blood flow. Nat. Commun. 15 (1), 8686. 10.1038/s41467-024-52969-0 39375369 PMC11458797

[B172] Lin Y. C. Guo Y. R. Miyagi A. Levring J. MacKinnon R. Scheuring S. (2019). Force-induced conformational changes in PIEZO1. Nature 573 (7773), 230–234. 10.1038/s41586-019-1499-2 31435018 PMC7258172

[B173] Lin Z. Xu G. Lu X. Wang H. Lu F. Xia X. (2025a). Piezo1 exacerbates inflammation-induced cartilaginous endplate degeneration by activating mitochondrial fission via the Ca(2+)/CaMKII/Drp1 axis. Aging Cell 24 (4), e14440. 10.1111/acel.14440 39610146 PMC11984661

[B174] Lin C. Y. Sassi A. Wu Y. Seaman K. Tang W. Song X. (2025b). Mechanotransduction pathways regulating YAP nuclear translocation under Yoda1 and vibration in osteocytes. Bone 190, 117283. 10.1016/j.bone.2024.117283 39413946

[B175] Lisek M. Tomczak J. Boczek T. Zylinska L. (2024). Calcium-associated proteins in neuroregeneration. Biomolecules 14 (2), 183. 10.3390/biom14020183 38397420 PMC10887043

[B176] Liu C. Dernburg A. F. (2024). Chemically induced proximity reveals a Piezo-dependent meiotic checkpoint at the oocyte nuclear envelope. Science 386 (6724), eadm7969. 10.1126/science.adm7969 39571011

[B177] Liu L. Yu H. Zhao H. Wu Z. Long Y. Zhang J. (2020). Matrix-transmitted paratensile signaling enables myofibroblast-fibroblast cross talk in fibrosis expansion. Proc. Natl. Acad. Sci. U. S. A. 117 (20), 10832–10838. 10.1073/pnas.1910650117 32358190 PMC7245086

[B178] Liu Z. Tang Y. He L. Geng B. He J. (2022a). Piezo1-mediated fluid shear stress promotes OPG and inhibits RANKL via NOTCH3 in MLO-Y4 osteocytes. Channels (Austin) 16 (1), 127–136. 10.1080/19336950.2022.2085379 35754337 PMC9721416

[B179] Liu Y. Tian H. Hu Y. Cao Y. Song H. Lan S. (2022b). Mechanosensitive Piezo1 is crucial for periosteal stem cell-mediated fracture healing. Int. J. Biol. Sci. 18 (10), 3961–3980. 10.7150/ijbs.71390 35844802 PMC9274506

[B180] Liu H. Hu J. Zheng Q. Feng X. Zhan F. Wang X. (2022c). Piezo1 channels as force sensors in mechanical force-related chronic inflammation. Front. Immunol. 13, 816149. 10.3389/fimmu.2022.816149 35154133 PMC8826255

[B181] Liu Z. Tong T. Sun J. Wu W. Zhang J. Cui Z. (2023a). Piezo1 in endothelial cells is involved in vitamin D-induced vascular calcification. Biochem. Biophys. Res. Commun. 638, 140–146. 10.1016/j.bbrc.2022.11.060 36455360

[B182] Liu Y. Zhang Z. Li J. Chang B. Lin Q. Wang F. (2023b). Piezo1 transforms mechanical stress into pro senescence signals and promotes osteoarthritis severity. Mech. Ageing Dev. 216, 111880. 10.1016/j.mad.2023.111880 37839614

[B183] Liu C. Xia Y. Fu S. Meng F. Feng B. Xu L. (2023c). Inhibition of Piezo1 ameliorates intestinal inflammation and limits the activation of group 3 innate lymphoid cells in experimental colitis. J. Innate Immun. 15 (1), 709–723. 10.1159/000533525 37725937 PMC10601687

[B184] Liu H. Zhou L. Wang X. Lin Y. Yi P. Xiong Y. (2024a). PIEZO1 as a new target for hyperglycemic stress-induced neuropathic injury: the potential therapeutic role of bezafibrate. Biomed. Pharmacother. 176, 116837. 10.1016/j.biopha.2024.116837 38815290

[B185] Liu Y. Zhang M. Wang C. Chen H. Su D. Yang C. (2024b). Human umbilical cord mesenchymal stromal cell-derived extracellular vesicles induce fetal wound healing features revealed by single-cell RNA sequencing. ACS Nano 18 (21), 13696–13713. 10.1021/acsnano.4c01401 38751164

[B186] Liu H. Li P. Zhao M. Ma T. Lv G. Liu L. (2024c). Activation of Piezo1 channels enhances spontaneous contractions of isolated human bladder strips via acetylcholine release from the mucosa. Eur. J. Pharmacol. 983, 176954. 10.1016/j.ejphar.2024.176954 39237075

[B187] Liu S. Yang X. Chen X. Zhang X. Jiang J. Yuan J. (2025a). An intermediate open structure reveals the gating transition of the mechanically activated PIEZO1 channel. Neuron 113 (4), 590–604.e6. 10.1016/j.neuron.2024.11.020 39719701

[B188] Liu Y. Yang Z. Na J. Chen X. Wang Z. Zheng L. (2025b). *In vitro* stretch modulates mitochondrial dynamics and energy metabolism to induce smooth muscle differentiation in mesenchymal stem cells. FASEB J. 39 (2), e70354. 10.1096/fj.202402944R 39840656

[B189] Liu S. Wu J. Meng L. Liu Y. Yu J. Yue J. (2025c). Piezo1-Induced nasal epithelial barrier dysfunction in allergic rhinitis. Inflammation 48 (4), 2824–2836. 10.1007/s10753-024-02234-9 39798033 PMC12336081

[B190] Lohberger B. Kaltenegger H. Weigl L. Mann A. Kullich W. Stuendl N. (2019). Mechanical exposure and diacerein treatment modulates integrin-FAK-MAPKs mechanotransduction in human osteoarthritis chondrocytes. Cell Signal 56, 23–30. 10.1016/j.cellsig.2018.12.010 30583016

[B191] Lopez-Cavestany M. Hahn S. B. Hope J. M. Reckhorn N. T. Greenlee J. D. Schwager S. C. (2023). Matrix stiffness induces epithelial-to-mesenchymal transition via Piezo1-regulated calcium flux in prostate cancer cells. iScience 26 (4), 106275. 10.1016/j.isci.2023.106275 36950111 PMC10025097

[B192] Lüchtefeld I. Pivkin I. V. Gardini L. Zare-Eelanjegh E. Gäbelein C. Ihle S. J. (2024). Dissecting cell membrane tension dynamics and its effect on Piezo1-mediated cellular mechanosensitivity using force-controlled nanopipettes. Nat. Methods 21 (6), 1063–1073. 10.1038/s41592-024-02277-8 38802520 PMC11166569

[B193] Luo S. Zhao X. Jiang J. Deng B. Liu S. Xu H. (2023a). Piezo1 specific deletion in macrophage protects the progression of liver fibrosis in mice. Theranostics 13 (15), 5418–5434. 10.7150/thno.86103 37908726 PMC10614683

[B194] Luo M. Ni K. Gu R. Qin Y. Guo J. Che B. (2023b). Chemical activation of Piezo1 alters biomechanical behaviors toward relaxation of cultured airway smooth muscle cells. Biol. Pharm. Bull. 46 (1), 1–11. 10.1248/bpb.b22-00209 36596517

[B195] Luo M. Gu R. Wang C. Guo J. Zhang X. Ni K. (2024a). High stretch associated with mechanical ventilation promotes Piezo1-Mediated migration of airway smooth muscle cells. Int. J. Mol. Sci. 25 (3), 1748. 10.3390/ijms25031748 38339025 PMC10855813

[B196] Luo S. Yang B. Xu H. Pan X. Chen X. Jue X. (2024b). Lithospermic acid improves liver fibrosis through Piezo1-mediated oxidative stress and inflammation. Phytomedicine 134, 155974. 10.1016/j.phymed.2024.155974 39217657

[B197] Luo M. Zhang X. Guo J. Gu R. Qin Y. Ni K. (2025). Piezo1 agonist Yoda1 induces rapid relaxation in cultured airway smooth muscle cells and bronchodilation in mouse models. Am. J. Respir. Cell Mol. Biol., rcmb.2024-0536OC. 10.1165/rcmb.2024-0536OC 40512988

[B198] Luu N. Bajpai A. Li R. Park S. Noor M. Ma X. (2024). Aging-associated decline in vascular smooth muscle cell mechanosensation is mediated by Piezo1 channel. Aging Cell 23 (2), e14036. 10.1111/acel.14036 37941511 PMC10861209

[B199] Ma S. Dubin A. E. Zhang Y. Mousavi S. A. R. Wang Y. Coombs A. M. (2021). A role of PIEZO1 in iron metabolism in mice and humans. Cell 184 (4), 969–982.e13. 10.1016/j.cell.2021.01.024 33571427 PMC7927959

[B200] Ma M. Li J. Li X. Jing M. Wang L. Jiang Y. (2025). Piezo1/ITGB1 synergizes with Ca(2+)/YAP signaling to propel bladder carcinoma progression via a stiffness-dependent positive feedback loop. Cancer Med. 14 (14), e71059. 10.1002/cam4.71059 40667648 PMC12264578

[B201] Malko P. Jia X. Wood I. Jiang L. H. (2023). Piezo1 channel-mediated Ca(2+) signaling inhibits lipopolysaccharide-induced activation of the NF-κB inflammatory signaling pathway and generation of TNF-α and IL-6 in microglial cells. Glia 71 (4), 848–865. 10.1002/glia.24311 36447422

[B202] Matute J. D. Duan J. Blumberg R. S. (2020). Microbial RNAs pressure Piezo1 to respond. Cell 182 (3), 542–544. 10.1016/j.cell.2020.07.015 32763186 PMC7880614

[B203] Mazal H. Wieser F. F. Bollschweiler D. Schambony A. Sandoghdar V. (2025). Cryo-light microscopy with angstrom precision deciphers structural conformations of PIEZO1 in its native state. Sci. Adv. 11 (34), eadw4402. 10.1126/sciadv.adw4402 40834076 PMC12366687

[B204] McHugh B. J. Buttery R. Lad Y. Banks S. Haslett C. Sethi T. (2010). Integrin activation by Fam38A uses a novel mechanism of R-Ras targeting to the endoplasmic reticulum. J. Cell Sci. 123 (Pt 1), 51–61. 10.1242/jcs.056424 20016066 PMC2794710

[B205] Merten A. L. Schöler U. Guo Y. Linsenmeier F. Martinac B. Friedrich O. (2024). High-content method for mechanosignaling studies using IsoStretcher technology and quantitative Ca(2+) imaging applied to Piezo1 in cardiac HL-1 cells. Cell Mol. Life Sci. 81 (1), 140. 10.1007/s00018-024-05159-6 38485771 PMC10940437

[B206] Michishita M. Yano K. Tomita K. I. Matsuzaki O. Kasahara K. I. (2016). Piezo1 expression increases in rat bladder after partial bladder outlet obstruction. Life Sci. 166, 1–7. 10.1016/j.lfs.2016.10.017 27756599

[B207] Mikhailov N. Plotnikova L. Singh P. Giniatullin R. Hämäläinen R. H. (2022). Functional characterization of mechanosensitive Piezo1 channels in trigeminal and somatic nerves in a neuron-on-chip model. Int. J. Mol. Sci. 23 (3), 1370. 10.3390/ijms23031370 35163293 PMC8835985

[B208] Miron T. R. Flood E. D. Tykocki N. R. Thompson J. M. Watts S. W. (2022). Identification of Piezo1 channels in perivascular adipose tissue (PVAT) and their potential role in vascular function. Pharmacol. Res. 175, 105995. 10.1016/j.phrs.2021.105995 34818570 PMC9301055

[B209] Mukhopadhyay A. Tsukasaki Y. Chan W. C. Le J. P. Kwok M. L. Zhou J. (2024). trans-Endothelial neutrophil migration activates bactericidal function via Piezo1 mechanosensing. Immunity 57 (1), 52–67.e10. 10.1016/j.immuni.2023.11.007 38091995 PMC10872880

[B210] Mylvaganam S. Plumb J. Yusuf B. Li R. Lu C. Y. Robinson L. A. (2022). The spectrin cytoskeleton integrates endothelial mechanoresponses. Nat. Cell Biol. 24 (8), 1226–1238. 10.1038/s41556-022-00953-5 35817960

[B211] Ni K. Che B. Gu R. Wang C. Pan Y. Li J. (2024). Single-cell hypertrophy promotes contractile function of cultured human airway smooth muscle cells via Piezo1 and YAP auto-regulation. Cells 13 (20), 1697. 10.3390/cells13201697 39451215 PMC11505810

[B212] Nieuwstraten J. Riester R. Hofmann U. K. Guilak F. Danalache M. (2025). Matrix metalloproteinases accelerate pericellular matrix breakdown and disrupt mechanotransduction in osteoarthritis. Acta Biomater. 195, 73–82. 10.1016/j.actbio.2025.02.034 39956307

[B213] O'Callaghan P. Engberg A. Eriksson O. Fatsis-Kavalopoulos N. Stelzl C. Sanchez G. (2022). Piezo1 activation attenuates thrombin-induced blebbing in breast cancer cells. J. Cell Sci. 135 (7), jcs258809. 10.1242/jcs.258809 35274124 PMC9016622

[B214] Obeidat A. M. Wood M. J. Adamczyk N. S. Ishihara S. Li J. Wang L. (2023). Piezo2 expressing nociceptors mediate mechanical sensitization in experimental osteoarthritis. Nat. Commun. 14 (1), 2479. 10.1038/s41467-023-38241-x 37120427 PMC10148822

[B215] Ochiai N. Etani Y. Noguchi T. Miura T. Kurihara T. Fukuda Y. (2024). The pivotal role of the Hes1/Piezo1 pathway in the pathophysiology of glucocorticoid-induced osteoporosis. JCI Insight 9 (23), e179963. 10.1172/jci.insight.179963 39641269 PMC11623955

[B216] Orsini E. M. Perelas A. Southern B. D. Grove L. M. Olman M. A. Scheraga R. G. (2021). Stretching the function of innate immune cells. Front. Immunol. 12, 767319. 10.3389/fimmu.2021.767319 34795674 PMC8593101

[B217] Page G. P. Kanias T. Guo Y. J. Lanteri M. C. Zhang X. Mast A. E. (2021). Multiple-ancestry genome-wide association study identifies 27 loci associated with measures of hemolysis following blood storage. J. Clin. Invest 131 (13), e146077. 10.1172/JCI146077 34014839 PMC8245173

[B218] Pan Y. Shi L. Z. Yoon C. W. Preece D. Gomez-Godinez V. Lu S. (2022). Mechanosensor Piezo1 mediates bimodal patterns of intracellular calcium and FAK signaling. EMBO J. 41 (17), e111799. 10.15252/embj.2022111799 35844093 PMC9433934

[B219] Pan X. Xu H. Ding Z. Luo S. Li Z. Wan R. (2024). Guizhitongluo tablet inhibits atherosclerosis and foam cell formation through regulating Piezo1/NLRP3 mediated macrophage pyroptosis. Phytomedicine 132, 155827. 10.1016/j.phymed.2024.155827 38955059

[B220] Pang R. Sun W. Yang Y. Wen D. Lin F. Wang D. (2024). PIEZO1 mechanically regulates the antitumour cytotoxicity of T lymphocytes. Nat. Biomed. Eng. 8 (9), 1162–1176. 10.1038/s41551-024-01188-5 38514773

[B221] Park H. Lee S. Furtado J. Robinson M. Antaya R. J. Oh S. P. (2025). PIEZO1 overexpression in hereditary hemorrhagic telangiectasia arteriovenous malformations. Circulation 152, 599–615. 10.1161/CIRCULATIONAHA.124.073630 40665909 PMC12270330

[B222] Pathak M. M. Nourse J. L. Tran T. Hwe J. Arulmoli J. Le D. T. T. (2014). Stretch-activated ion channel Piezo1 directs lineage choice in human neural stem cells. Proc. Natl. Acad. Sci. U. S. A. 111 (45), 16148–16153. 10.1073/pnas.1409802111 25349416 PMC4234578

[B223] Peng F. Liao M. Jin W. Liu W. Li Z. Fan Z. (2024). 2-APQC, a small-molecule activator of Sirtuin-3 (SIRT3), alleviates myocardial hypertrophy and fibrosis by regulating mitochondrial homeostasis. Signal Transduct. Target Ther. 9 (1), 133. 10.1038/s41392-024-01816-1 38744811 PMC11094072

[B224] Peng F. Sun M. Jing X. Chen F. Cao T. Li Z. (2025). Piezo1 promotes intervertebral disc degeneration through the Ca(2+)/F-actin/Yap signaling axis. Mol. Med. 31 (1), 90. 10.1186/s10020-025-01147-z 40057686 PMC11889814

[B225] Peussa H. Fedele C. Tran H. Marttinen M. Fadjukov J. Mäntylä E. (2023). Light-induced nanoscale deformation in azobenzene thin film triggers rapid intracellular Ca(2+) increase via mechanosensitive cation channels. Adv. Sci. (Weinh) 10 (35), e2206190. 10.1002/advs.202206190 37946608 PMC10724422

[B226] Poole R. A. Wang Q. Ray A. Takabe K. Opyrchal M. Katsuta E. (2024). Increased PIEZO1 expression is associated with worse clinical outcomes in hormone-receptor-negative breast cancer patients. Cancers (Basel) 16 (4), 683. 10.3390/cancers16040683 38398074 PMC10887014

[B227] Porto Ribeiro T. Barbeau S. Baudrimont I. Vacher P. Freund-Michel V. Cardouat G. (2022). Piezo1 channel activation reverses pulmonary artery vasoconstriction in an early rat model of pulmonary hypertension: the role of Ca(2+) influx and Akt-eNOS pathway. Cells 11 (15), 2349. 10.3390/cells11152349 35954193 PMC9367624

[B228] Qi Y. Andolfi L. Frattini F. Mayer F. Lazzarino M. Hu J. (2015). Membrane stiffening by STOML3 facilitates mechanosensation in sensory neurons. Nat. Commun. 6, 8512. 10.1038/ncomms9512 26443885 PMC4633829

[B229] Qi M. Liu R. Zhang F. Yao Z. Zhou M. L. Jiang X. (2024). Roles of mechanosensitive ion channel PIEZO1 in the pathogenesis of brain injury after experimental intracerebral hemorrhage. Neuropharmacology 251, 109896. 10.1016/j.neuropharm.2024.109896 38490299

[B230] Qian W. Hadi T. Silvestro M. Ma X. Rivera C. F. Bajpai A. (2022). Microskeletal stiffness promotes aortic aneurysm by sustaining pathological vascular smooth muscle cell mechanosensation via Piezo1. Nat. Commun. 13 (1), 512. 10.1038/s41467-021-27874-5 35082286 PMC8791986

[B231] Qin L. He T. Chen S. Yang D. Yi W. Cao H. (2021). Roles of mechanosensitive channel Piezo1/2 proteins in skeleton and other tissues. Bone Res. 9 (1), 44. 10.1038/s41413-021-00168-8 34667178 PMC8526690

[B232] Qin C. Feng Y. Yin Z. Wang C. Yin R. Li Y. (2024). The PIEZO1/miR-155-5p/GDF6/SMAD2/3 signaling axis is involved in inducing the occurrence and progression of osteoarthritis under excessive mechanical stress. Cell Signal 118, 111142. 10.1016/j.cellsig.2024.111142 38508350

[B233] Qu P. Zhang H. (2025). The dual role of Piezo1 in tumor cells and immune cells: a new target for cancer therapy. Front. Immunol. 16, 1635388. 10.3389/fimmu.2025.1635388 40821847 PMC12350401

[B234] Qu J. Zong H. F. Shan Y. Zhang S. C. Guan W. P. Yang Y. (2023). Piezo1 suppression reduces demyelination after intracerebral hemorrhage. Neural Regen. Res. 18 (8), 1750–1756. 10.4103/1673-5374.361531 36751801 PMC10154511

[B235] Querio G. Geddo F. Antoniotti S. Femminò S. Gallo M. P. Penna C. (2025). Stay connected: the myoendothelial junction proteins in vascular function and dysfunction. Vasc. Pharmacol. 158, 107463. 10.1016/j.vph.2025.107463 39814089

[B236] Ran L. Ye T. Erbs E. Ehl S. Spassky N. Sumara I. (2023). KCNN4 links PIEZO-dependent mechanotransduction to NLRP3 inflammasome activation. Sci. Immunol. 8 (90), eadf4699. 10.1126/sciimmunol.adf4699 38134241

[B237] Rashidi N. Harasymowicz N. S. Savadipour A. Steward N. Tang R. Oswald S. (2025). PIEZO1-mediated mechanotransduction regulates collagen synthesis on nanostructured 2D and 3D models of fibrosis. Acta Biomater. 193, 242–254. 10.1016/j.actbio.2024.12.034 39675497 PMC13276715

[B238] Ren X. Zhuang H. Li B. Jiang F. Zhang Y. Zhou P. (2023). Gsmtx4 alleviated osteoarthritis through Piezo1/Calcineurin/NFAT1 signaling axis under excessive mechanical strain. Int. J. Mol. Sci. 24 (4), 4022. 10.3390/ijms24044022 36835440 PMC9961447

[B239] Rendon C. J. Flood E. Thompson J. M. Chirivi M. Watts S. W. Contreras G. A. (2022). PIEZO1 mechanoreceptor activation reduces adipogenesis in perivascular adipose tissue preadipocytes. Front. Endocrinol. (Lausanne) 13, 995499. 10.3389/fendo.2022.995499 36120469 PMC9471253

[B240] Retailleau K. Duprat F. Arhatte M. Ranade S. S. Peyronnet R. Martins J. R. (2015). Piezo1 in smooth muscle cells is involved in hypertension-dependent arterial remodeling. Cell Rep. 13 (6), 1161–1171. 10.1016/j.celrep.2015.09.072 26526998

[B241] Risinger M. Kalfa T. A. (2020). Red cell membrane disorders: structure meets function. Blood 136 (11), 1250–1261. 10.1182/blood.2019000946 32702754 PMC7483429

[B242] Rode B. Shi J. Endesh N. Drinkhill M. J. Webster P. J. Lotteau S. J. (2017). Piezo1 channels sense whole body physical activity to reset cardiovascular homeostasis and enhance performance. Nat. Commun. 8 (1), 350. 10.1038/s41467-017-00429-3 28839146 PMC5571199

[B243] Rong S. Zhang L. Wang J. Dong H. (2024). Regulatory role of Piezo1 channel in endothelium-dependent hyperpolarization-mediated vasorelaxation of small resistance vessels and its anti-inflammatory action. Life Sci. 336, 122326. 10.1016/j.lfs.2023.122326 38056769

[B244] Rosato B. E. D'Onofrio V. Marra R. Nostroso A. Esposito F. M. Iscaro A. (2025). RAS signaling pathway is essential in regulating PIEZO1-mediated hepatic iron overload in dehydrated hereditary stomatocytosis. Am. J. Hematol. 100 (1), 52–65. 10.1002/ajh.27523 39558179 PMC11625994

[B245] Saotome K. Murthy S. E. Kefauver J. M. Whitwam T. Patapoutian A. Ward A. B. (2018). Structure of the mechanically activated ion channel Piezo1. Nature 554 (7693), 481–486. 10.1038/nature25453 29261642 PMC6010196

[B246] Savadipour A. Nims R. J. Rashidi N. Garcia-Castorena J. M. Tang R. Marushack G. K. (2023). Membrane stretch as the mechanism of activation of PIEZO1 ion channels in chondrocytes. Proc. Natl. Acad. Sci. U. S. A. 120 (30), e2221958120. 10.1073/pnas.2221958120 37459546 PMC10372640

[B247] Scapin G. Cillis J. L. Goulard M. C. Patch T. C. Gomez Limia C. E. Ding Y. (2025). PIEZO1 activation-mediated generation of transgene-free long-term hematopoietic stem cells. Am. J. Hematol. 100 (6), 963–979. 10.1002/ajh.27689 40320799

[B248] Scorza S. Brunetti V. Scarpellino G. Certini M. Gerbino A. Moccia F. (2025). Targeting the Ca(2+) signaling toolkit as an alternative strategy to mitigate SARS-CoV-2-induced cardiovascular adverse events. Vasc. Pharmacol. 158, 107458. 10.1016/j.vph.2024.107458 39701403

[B249] Segel M. Neumann B. Hill M. Weber I. P. Viscomi C. Zhao C. (2019). Niche stiffness underlies the ageing of central nervous system progenitor cells. Nature 573 (7772), 130–134. 10.1038/s41586-019-1484-9 31413369 PMC7025879

[B250] Shah V. Patel S. Shah J. (2022). Emerging role of piezo ion channels in cardiovascular development. Dev. Dyn. 251 (2), 276–286. 10.1002/dvdy.401 34255896

[B251] Shahidullah M. Rosales J. L. Delamere N. (2022). Activation of Piezo1 increases Na,K-ATPase-Mediated ion transport in mouse lens. Int. J. Mol. Sci. 23 (21), 12870. 10.3390/ijms232112870 36361659 PMC9656371

[B252] Shan J. Xie W. Betzenhauser M. Reiken S. Chen B. X. Wronska A. (2012). Calcium leak through ryanodine receptors leads to atrial fibrillation in 3 mouse models of catecholaminergic polymorphic ventricular tachycardia. Circ. Res. 111 (6), 708–717. 10.1161/CIRCRESAHA.112.273342 22828895 PMC3734386

[B253] Shen B. Tasdogan A. Ubellacker J. M. Zhang J. Nosyreva E. D. Du L. (2021). A mechanosensitive peri-arteriolar niche for osteogenesis and lymphopoiesis. Nature 591 (7850), 438–444. 10.1038/s41586-021-03298-5 33627868 PMC7979521

[B254] Shi Y. Wang H. Liu Y. Long M. Ding N. Mi L. (2023). Genetic abnormalities assist in pathological diagnosis and EBV-positive cell density impact survival in Chinese angioimmunoblastic T-cell lymphoma patients. Chin. J. Cancer Res. 35 (5), 536–549. 10.21147/j.issn.1000-9604.2023.05.10 37969960 PMC10643336

[B255] Shin W. Jeong S. Lee J. U. Shin J. Kim H. H. (2022). Magnetogenetics with Piezo1 mechanosensitive ion channel for CRISPR gene editing. Nano Lett. 22 (18), 7415–7422. 10.1021/acs.nanolett.2c02314 36069378

[B256] Shinge S. Zhang D. Din A. U. Yu F. Nie Y. (2022). Emerging Piezo1 signaling in inflammation and atherosclerosis; a potential therapeutic target. Int. J. Biol. Sci. 18 (3), 923–941. 10.7150/ijbs.63819 35173527 PMC8771847

[B257] Sitnikova V. Nurkhametova D. Braidotti N. Ciubotaru C. D. Giudice L. Impola U. (2025). Increased activity of Piezo1 channel in red blood cells is associated with Alzheimer's disease-related dementia. Alzheimers Dement. 21 (6), e70368. 10.1002/alz.70368 40534259 PMC12177196

[B258] Smith K. A. Chuntharpursat-Bon E. Povstyan O. V. Debant M. Kinsella J. A. Revill C. (2025). Regulation of PIEZO1 channel force sensitivity by interblade handshaking. Sci. Adv. 11 (24), eadt7046. 10.1126/sciadv.adt7046 40512861 PMC12164982

[B259] So C. L. Robitaille M. Sadras F. McCullough M. H. Milevskiy M. J. G. Goodhill G. J. (2024). Cellular geometry and epithelial-mesenchymal plasticity intersect with PIEZO1 in breast cancer cells. Commun. Biol. 7 (1), 467. 10.1038/s42003-024-06163-z 38632473 PMC11024093

[B260] Solis A. G. Bielecki P. Steach H. R. Sharma L. Harman C. C. D. Yun S. (2019). Mechanosensation of cyclical force by PIEZO1 is essential for innate immunity. Nature 573 (7772), 69–74. 10.1038/s41586-019-1485-8 31435009 PMC6939392

[B261] Song Y. Li D. Farrelly O. Miles L. Li F. Kim S. E. (2019). The mechanosensitive ion channel piezo inhibits axon regeneration. Neuron 102 (2), 373–389.e6. 10.1016/j.neuron.2019.01.050 30819546 PMC6487666

[B262] Strittmatter T. Argast P. Buchman P. Krawczyk K. Fussenegger M. (2021). Control of gene expression in engineered mammalian cells with a programmable shear-stress inducer. Biotechnol. Bioeng. 118 (12), 4751–4759. 10.1002/bit.27939 34506645 PMC9292429

[B263] Su S. A. Zhang Y. Li W. Xi Y. Lu Y. Shen J. (2023). Cardiac Piezo1 exacerbates lethal ventricular arrhythmogenesis by linking mechanical stress with Ca(2+) handling after myocardial infarction. Res. (Wash D C) 6, 0165. 10.34133/research.0165 37303604 PMC10255393

[B264] Sugisawa E. Takayama Y. Takemura N. Kondo T. Hatakeyama S. Kumagai Y. (2020). RNA sensing by gut Piezo1 is essential for systemic serotonin synthesis. Cell 182 (3), 609–624.e21. 10.1016/j.cell.2020.06.022 32640190

[B265] Sun X. F. Qiao W. W. Meng L. Y. Bian Z. (2022). PIEZO1 ion channels mediate mechanotransduction in odontoblasts. J. Endod. 48 (6), 749–758. 10.1016/j.joen.2022.02.005 35219748

[B266] Sun Y. Fang Y. Li X. Li J. Liu D. Wei M. (2023). A static magnetic field enhances the repair of osteoarthritic cartilage by promoting the migration of stem cells and chondrogenesis. J. Orthop. Transl. 39, 43–54. 10.1016/j.jot.2022.11.007 36721767 PMC9849874

[B267] Sun M. Mao S. Wu C. Zhao X. Guo C. Hu J. (2024). Piezo1-Mediated neurogenic inflammatory Cascade exacerbates ventricular remodeling after myocardial infarction. Circulation 149 (19), 1516–1533. 10.1161/CIRCULATIONAHA.123.065390 38235590

[B268] Sun L. Wang Y. Kan T. Wang H. Cui J. (2025a). Elevated expression of Piezo1 activates the cGAS-STING pathway in chondrocytes by releasing mitochondrial DNA. Osteoarthr. Cartil. 33 (5), 601–615. 10.1016/j.joca.2025.02.778 39978573

[B269] Sun Y. Y. Zhang X. C. Jiang Y. Y. Guan X. M. Li Z. R. Chu X. M. (2025b). The role of Piezo1 in cardiovascular diseases: from molecular mechanisms to targeted therapeutic potential. Int. J. Biol. Macromol. 318 (Pt 2), 144843. 10.1016/j.ijbiomac.2025.144843 40456339

[B270] Swain S. M. Liddle R. A. (2023). Mechanosensing piezo channels in gastrointestinal disorders. J. Clin. Invest 133 (19), e171955. 10.1172/JCI171955 37781915 PMC10541197

[B271] Swain S. M. Romac J. M. Shahid R. A. Pandol S. J. Liedtke W. Vigna S. R. (2020). TRPV4 channel opening mediates pressure-induced pancreatitis initiated by Piezo1 activation. J. Clin. Invest 130 (5), 2527–2541. 10.1172/JCI134111 31999644 PMC7190979

[B272] Swain S. M. Romac J. M. Vigna S. R. Liddle R. A. (2022). Piezo1-mediated stellate cell activation causes pressure-induced pancreatic fibrosis in mice. JCI Insight 7 (8), e158288. 10.1172/jci.insight.158288 35451372 PMC9089793

[B273] Swiatlowska P. Tipping W. Marhuenda E. Severi P. Fomin V. Yang Z. (2024). Hypertensive pressure mechanosensing alone triggers lipid droplet accumulation and transdifferentiation of vascular smooth muscle cells to foam cells. Adv. Sci. (Weinh) 11 (9), e2308686. 10.1002/advs.202308686 38145971 PMC10916670

[B274] Szabó L. Balogh N. Tóth A. Angyal Á. Gönczi M. Csiki D. M. (2022). The mechanosensitive Piezo1 channels contribute to the arterial medial calcification. Front. Physiol. 13, 1037230. 10.3389/fphys.2022.1037230 36439266 PMC9685409

[B275] Tang H. Zeng R. He E. Zhang I. Ding C. Zhang A. (2022). Piezo-type mechanosensitive ion channel component 1 (Piezo1): a promising therapeutic target and its modulators. J. Med. Chem. 65 (9), 6441–6453. 10.1021/acs.jmedchem.2c00085 35466678

[B276] Tang Y. Zhao C. Zhuang Y. Zhong A. Wang M. Zhang W. (2023). Mechanosensitive Piezo1 protein as a novel regulator in macrophages and macrophage-mediated inflammatory diseases. Front. Immunol. 14, 1149336. 10.3389/fimmu.2023.1149336 37334369 PMC10275567

[B277] Tang L. Xie D. Wang S. Gao C. Pan S. (2024a). Piezo1 knockout improves post-stroke cognitive dysfunction by inhibiting the Interleukin-6 (IL-6)/Glutathione peroxidase 4 (GPX4) pathway. J. Inflamm. Res. 17, 2257–2270. 10.2147/JIR.S448903 38633449 PMC11022880

[B278] Tang H. Hao R. Ma D. Yao Y. Ding C. Zhang X. (2024b). Structural modification and pharmacological evaluation of (Thiadiazol-2-yl)pyrazines as novel Piezo1 agonists for the intervention of disuse osteoporosis. J. Med. Chem. 67 (21), 19837–19851. 10.1021/acs.jmedchem.4c02224 39462841

[B279] Tao H. Zhu M. Lau K. Whitley O. K. W. Samani M. Xiao X. (2019). Oscillatory cortical forces promote three dimensional cell intercalations that shape the murine mandibular arch. Nat. Commun. 10 (1), 1703. 10.1038/s41467-019-09540-z 30979871 PMC6461694

[B280] Thoudam T. Chanda D. Lee J. Y. Jung M. K. Sinam I. S. Kim B. G. (2023). Enhanced Ca(2+)-channeling complex formation at the ER-mitochondria interface underlies the pathogenesis of alcohol-associated liver disease. Nat. Commun. 14 (1), 1703. 10.1038/s41467-023-37214-4 36973273 PMC10042999

[B281] Uchinuma M. Taketani Y. Kanaya R. Yamane Y. Shiota K. Suzuki R. (2024). Role of Piezo1 in modulating the RANKL/OPG ratio in mouse osteoblast cells exposed to Porphyromonas gingivalis lipopolysaccharide and mechanical stress. J. Periodontal Res. 59 (4), 749–757. 10.1111/jre.13265 38623787

[B282] Vanderroost J. Parpaite T. Avalosse N. Henriet P. Pierreux C. E. Lorent J. H. (2023). Piezo1 is required for myoblast migration and involves polarized clustering in association with cholesterol and GM1 ganglioside. Cells 12 (24), 2784. 10.3390/cells12242784 38132106 PMC10741634

[B283] Vasileva V. Y. Sudarikova A. V. Chubinskiy-Nadezhdin V. I. (2025). Functional coupling of Piezo1 channels and Ca(2+)-activated ion channels in the plasma membrane: fine-tunable interplay with wide-range signaling effects. Am. J. Physiol. Cell Physiol. 328 (4), C1338–C1345. 10.1152/ajpcell.00094.2025 40099870

[B284] Velasco-Estevez M. Rolle S. O. Mampay M. Dev K. K. Sheridan G. K. (2020). Piezo1 regulates calcium oscillations and cytokine release from astrocytes. Glia 68 (1), 145–160. 10.1002/glia.23709 31433095

[B285] Velasco-Estevez M. Koch N. Klejbor I. Caratis F. Rutkowska A. (2022). Mechanoreceptor Piezo1 is downregulated in multiple sclerosis brain and is involved in the maturation and migration of oligodendrocytes *in vitro* . Front. Cell Neurosci. 16, 914985. 10.3389/fncel.2022.914985 35722613 PMC9204635

[B286] Vervliet T. Parys J. B. Bultynck G. (2015). Bcl-2 and FKBP12 bind to IP3 and ryanodine receptors at overlapping sites: the complexity of protein-protein interactions for channel regulation. Biochem. Soc. Trans. 43 (3), 396–404. 10.1042/BST20140298 26009182

[B287] Wang S. Chennupati R. Kaur H. Iring A. Wettschureck N. Offermanns S. (2016). Endothelial cation channel PIEZO1 controls blood pressure by mediating flow-induced ATP release. J. Clin. Invest 126 (12), 4527–4536. 10.1172/JCI87343 27797339 PMC5127677

[B288] Wang L. Zhou H. Zhang M. Liu W. Deng T. Zhao Q. (2019). Structure and mechanogating of the Mammalian tactile channel PIEZO2. Nature 573 (7773), 225–229. 10.1038/s41586-019-1505-8 31435011

[B289] Wang L. You X. Lotinun S. Zhang L. Wu N. Zou W. (2020). Mechanical sensing protein PIEZO1 regulates bone homeostasis via osteoblast-osteoclast crosstalk. Nat. Commun. 11 (1), 282. 10.1038/s41467-019-14146-6 31941964 PMC6962448

[B290] Wang X. Cheng G. Miao Y. Qiu F. Bai L. Gao Z. (2021a). Piezo type mechanosensitive ion channel component 1 facilitates gastric cancer omentum metastasis. J. Cell Mol. Med. 25 (4), 2238–2253. 10.1111/jcmm.16217 33439514 PMC7882944

[B291] Wang Z. Chen J. Babicheva A. Jain P. P. Rodriguez M. Ayon R. J. (2021b). Endothelial upregulation of mechanosensitive channel Piezo1 in pulmonary hypertension. Am. J. Physiol. Cell Physiol. 321 (6), C1010–C1027. 10.1152/ajpcell.00147.2021 34669509 PMC8714987

[B292] Wang M. Herbst R. S. Boshoff C. (2021c). Toward personalized treatment approaches for non-small-cell lung cancer. Nat. Med. 27 (8), 1345–1356. 10.1038/s41591-021-01450-2 34385702

[B293] Wang S. Li W. Zhang P. Wang Z. Ma X. Liu C. (2022a). Mechanical overloading induces GPX4-regulated chondrocyte ferroptosis in osteoarthritis via Piezo1 channel facilitated calcium influx. J. Adv. Res. 41, 63–75. 10.1016/j.jare.2022.01.004 36328754 PMC9637484

[B294] Wang S. Wang B. Shi Y. Möller T. Stegmeyer R. I. Strilic B. (2022b). Mechanosensation by endothelial PIEZO1 is required for leukocyte diapedesis. Blood 140 (3), 171–183. 10.1182/blood.2021014614 35443048 PMC9305087

[B295] Wang M. Zhou X. Zhou S. Jiang J. Wu W. (2023). Mechanical force drives the initial mesenchymal-epithelial interaction during skin organoid development. Theranostics 13 (9), 2930–2945. 10.7150/thno.83217 37284452 PMC10240816

[B296] Wang B. Shao W. Zhao Y. Li Z. Wang P. Lv X. (2024a). Radial extracorporeal shockwave promotes osteogenesis-angiogenesis coupling of bone marrow stromal cells from senile osteoporosis via activating the Piezo1/CaMKII/CREB axis. Bone 187, 117196. 10.1016/j.bone.2024.117196 39004161

[B297] Wang Y. M. Chu T. J. Wan R. T. Niu W. P. Bian Y. F. Li J. (2024b). Quercetin ameliorates atherosclerosis by inhibiting inflammation of vascular endothelial cells via Piezo1 channels. Phytomedicine 132, 155865. 10.1016/j.phymed.2024.155865 39004029

[B298] Wang X. Tao J. Zhou J. Shu Y. Xu J. (2024c). Excessive load promotes temporomandibular joint chondrocyte apoptosis via Piezo1/endoplasmic reticulum stress pathway. J. Cell Mol. Med. 28 (11), e18472. 10.1111/jcmm.18472 38842129 PMC11154833

[B299] Wang J. Zhuang H. Li C. Cai R. Shi H. Pang B. (2025). Ligustrazine nano-drug delivery system ameliorates doxorubicin-mediated myocardial injury via piezo-type mechanosensitive ion channel component 1-prohibitin 2-mediated mitochondrial quality surveillance. J. Nanobiotechnology 23 (1), 383. 10.1186/s12951-025-03420-z 40426179 PMC12117932

[B300] Wang J. Fu C. Chang S. Stephens C. Li H. Wang D. (2025a). PIEZO1-mediated calcium signaling reinforces mechanical properties of hair follicle stem cells to promote quiescence. Sci. Adv. 11 (22), eadt2771. 10.1126/sciadv.adt2771 40435254 PMC12118625

[B301] Wang J. Zhao W. Bai W. Dong D. Wang H. Qi X. (2025b). PIEZO1 mediates mechanical reprogramming of neutrophils for proangiogenic specialization in the lung. J. Clin. Invest 135 (11), e183796. 10.1172/JCI183796 40454475 PMC12126238

[B302] Wang J. Jing F. Zhao Y. You Z. Zhang A. Qin S. (2025c). Piezo1: structural pharmacology and mechanotransduction mechanisms. Trends Pharmacol. Sci. 46 (8), 752–770. 10.1016/j.tips.2025.06.009 40750459

[B303] Wang H. Gou Z. Chen S. Lu L. (2025d). Piezo1 is a pathogenic gene and therapeutic target for neurological diseases. Int. J. Neurosci., 1–16. 10.1080/00207454.2025.2496819 40276938

[B304] Wang X. Wang J. Zhang Y. He Y. Chen S. (2025e). Piezo1 regulates fibrocartilage stem cell in cartilage growth and osteoarthritis. Osteoarthr. Cartil. 33 (8), 980–991. 10.1016/j.joca.2025.04.013 40345612

[B305] Wang Y. Yang Q. Dong Y. Wang L. Zhang Z. Niu R. (2025f). Piezo1-directed neutrophil extracellular traps regulate macrophage differentiation during influenza virus infection. Cell Death Dis. 16 (1), 60. 10.1038/s41419-025-07395-5 39890818 PMC11785962

[B306] Wang C. Luo S. Yan Y. Li J. Niu W. Hong T. (2025g). Endothelial Piezo1 stimulates angiogenesis to offer protection against intestinal ischemia-reperfusion injury in mice. Mol. Med. 31 (1), 147. 10.1186/s10020-025-01197-3 40263994 PMC12016420

[B307] Wang W. Huang M. Huang X. Ma K. Luo M. Yang N. (2025h). GsMTx4-blocked PIEZO1 channel promotes myogenic differentiation and alleviates myofiber damage in Duchenne muscular dystrophy. Skelet. Muscle 15 (1), 13. 10.1186/s13395-025-00383-5 40361216 PMC12076844

[B308] Webster H. (2025). Piezo1 as a therapeutic target for glucocorticoid-induced osteoporosis. Nat. Rev. Rheumatol. 21 (3), 127. 10.1038/s41584-024-01215-4 39794513

[B309] Wenqiang D. Novin A. Liu Y. Afzal J. Suhail Y. Liu S. (2024). Scar matrix drives Piezo1 mediated stromal inflammation leading to placenta accreta spectrum. Nat. Commun. 15 (1), 8379. 10.1038/s41467-024-52351-0 39333481 PMC11436960

[B310] Woll K. A. Van Petegem F. (2022). Calcium-release channels: structure and function of IP(3) receptors and ryanodine receptors. Physiol. Rev. 102 (1), 209–268. 10.1152/physrev.00033.2020 34280054

[B311] Wu P. Nielsen T. E. Clausen M. H. (2015). FDA-approved small-molecule kinase inhibitors. Trends Pharmacol. Sci. 36 (7), 422–439. 10.1016/j.tips.2015.04.005 25975227

[B312] Wu R. W. Lian W. S. Chen Y. S. Ko J. Y. Wang S. Y. Jahr H. (2021). Piezoelectric microvibration mitigates estrogen loss-induced osteoporosis and promotes Piezo1, MicroRNA-29a, and Wnt3a signaling in osteoblasts. Int. J. Mol. Sci. 22 (17), 9476. 10.3390/ijms22179476 34502380 PMC8431199

[B313] Wu J. Chen Y. Liao Z. Liu H. Zhang S. Zhong D. (2022). Self-amplifying loop of NF-κB and periostin initiated by PIEZO1 accelerates mechano-induced senescence of nucleus pulposus cells and intervertebral disc degeneration. Mol. Ther. 30 (10), 3241–3256. 10.1016/j.ymthe.2022.05.021 35619555 PMC9552911

[B314] Xiao B. (2024). Mechanisms of mechanotransduction and physiological roles of PIEZO channels. Nat. Rev. Mol. Cell Biol. 25 (11), 886–903. 10.1038/s41580-024-00773-5 39251883

[B315] Xie Z. Y. Dong W. Zhang L. Wang M. J. Xiao Z. M. Zhang Y. H. (2022). NFAT inhibitor 11R-VIVIT ameliorates mouse renal fibrosis after ischemia-reperfusion-induced acute kidney injury. Acta Pharmacol. Sin. 43 (8), 2081–2093. 10.1038/s41401-021-00833-y 34937917 PMC9343462

[B316] Xie L. Wang X. Ma Y. Ma H. Shen J. Chen J. (2023). Piezo1 (Piezo-Type mechanosensitive ion channel component 1)-Mediated mechanosensation in macrophages impairs perfusion recovery after hindlimb ischemia in mice. Arterioscler. Thromb. Vasc. Biol. 43 (4), 504–518. 10.1161/ATVBAHA.122.318625 36756881

[B317] Xie Z. Rose L. Feng J. Zhao Y. Lu Y. Kane H. (2025a). Enteric neuronal Piezo1 maintains mechanical and immunological homeostasis by sensing force. Cell 188 (9), 2417–2432.e19. 10.1016/j.cell.2025.02.031 40132579 PMC12048284

[B318] Xie W. Lückemeyer D. D. Qualls K. A. Prudente A. S. Berta T. Gu M. (2025b). Vascular motion in the dorsal root ganglion sensed by Piezo2 in sensory neurons triggers episodic pain. Neuron 113 (11), 1774–1788.e5. 10.1016/j.neuron.2025.03.006 40154477 PMC12140901

[B319] Xie W. Yu X. Yang Q. Ke N. Wang P. Kong H. (2025c). The immunomechanical checkpoint PYK2 governs monocyte-to-macrophage differentiation in pancreatic cancer. Cancer Discov. 15 (8), 1740–1765. 10.1158/2159-8290.CD-24-1712 40338055

[B320] Xiong Y. Dong L. Bai Y. Tang H. Li S. Luo D. (2022). Piezo1 activation facilitates ovarian cancer metastasis via Hippo/YAP signaling axis. Channels (Austin) 16 (1), 159–166. 10.1080/19336950.2022.2099381 35942515 PMC9367648

[B321] Xu X. Z. (2016). Demystifying mechanosensitive piezo ion channels. Neurosci. Bull. 32 (3), 307–309. 10.1007/s12264-016-0033-x 27164907 PMC5563776

[B322] Xu X. Liu S. Liu H. Ru K. Jia Y. Wu Z. (2021). Piezo channels: Awesome mechanosensitive structures in cellular mechanotransduction and their role in bone. Int. J. Mol. Sci. 22 (12), 6429. 10.3390/ijms22126429 34208464 PMC8234635

[B323] Xu Y. Xiong Y. Liu Y. Bai T. Zheng G. (2023). Activation of goblet cell Piezo1 alleviates mucus barrier damage in mice exposed to WAS by inhibiting H3K9me3 modification. Cell Biosci. 13 (1), 7. 10.1186/s13578-023-00952-5 36631841 PMC9835388

[B324] Xu F. Xin Q. Ren M. Shi P. Wang B. (2024). Inhibition of piezo1 prevents chronic cerebral hypoperfusion-induced cognitive impairment and blood brain barrier disruption. Neurochem. Int. 175, 105702. 10.1016/j.neuint.2024.105702 38401846

[B325] Xu H. Chen X. Luo S. Jiang J. Pan X. He Y. (2025a). Cardiomyocyte-specific Piezo1 deficiency mitigates ischemia-reperfusion injury by preserving mitochondrial homeostasis. Redox Biol. 79, 103471. 10.1016/j.redox.2024.103471 39740362 PMC11750285

[B326] Xu L. Li T. Cao Y. He Y. Shao Z. Liu S. (2025b). PIEZO1 mediates periostin+ myofibroblast activation and pulmonary fibrosis in mice. J. Clin. Invest 135 (11), e184158. 10.1172/JCI184158 40454481 PMC12126248

[B327] Xu S. Gu L. Bao B. Liu Q. Jin Q. Ma Y. (2025c). Mechanistic insights into the neuroprotective effects of low-intensity transcranial ultrasound stimulation in post-cardiac arrest brain injury: modulation of the Piezo1-Dkk3/PI3K-Akt pathway. Brain Behav. Immun. 127, 341–357. 10.1016/j.bbi.2025.03.027 40118226

[B328] Xu X. Zhang Q. Lv Z. Cheng C. Zha J. Shu H. (2025d). Unraveling the deadly dance: endothelial cells and neutrophils in sepsis-induced acute lung injury/acute respiratory distress syndrome. Front. Cell Dev. Biol. 13, 1551138. 10.3389/fcell.2025.1551138 40476000 PMC12137324

[B329] Xu T. Zhang L. Lu X. Ji W. Chen K. (2025e). Piezo1 mediates ultrasound-stimulated dopaminergic neuron protection via synaptic vesicle recycling and ferroptosis inhibition. Neurosci. Bull. 10.1007/s12264-025-01420-5 40439849 PMC12569254

[B330] Xue Z. Jiang Y. Meng B. Lu L. Hao M. Zhang Y. (2024). Apoptotic vesicle-mediated senolytics requires mechanical loading. Theranostics 14 (12), 4730–4746. 10.7150/thno.98763 39239523 PMC11373628

[B331] Yan Z. Niu L. Wang S. Gao C. Pan S. (2024a). Intestinal Piezo1 aggravates intestinal barrier dysfunction during sepsis by mediating Ca(2+) influx. J. Transl. Med. 22 (1), 332. 10.1186/s12967-024-05076-z 38575957 PMC10996241

[B332] Yan W. Cheng J. Wu H. Gao Z. Li Z. Cao C. (2024b). Vascular smooth muscle cells transdifferentiate into chondrocyte-like cells and facilitate meniscal fibrocartilage regeneration. Res. (Wash D C) 7, 0555. 10.34133/research.0555 39717465 PMC11665451

[B333] Yang X. Lin C. Chen X. Li S. Xiao B. (2022a). Structure deformation and curvature sensing of PIEZO1 in lipid membranes. Nature 604 (7905), 377–383. 10.1038/s41586-022-04574-8 35388220

[B334] Yang S. Miao X. Arnold S. Li B. Ly A. T. Wang H. (2022b). Membrane curvature governs the distribution of Piezo1 in live cells. Nat. Commun. 13 (1), 7467. 10.1038/s41467-022-35034-6 36463216 PMC9719557

[B335] Yang X. Zeng H. Wang L. Luo S. Zhou Y. (2022c). Activation of Piezo1 downregulates renin in juxtaglomerular cells and contributes to blood pressure homeostasis. Cell Biosci. 12 (1), 197. 10.1186/s13578-022-00931-2 36471394 PMC9720979

[B336] Yang K. He X. Wu Z. Yin Y. Pan H. Zhao X. (2022d). The emerging roles of piezo1 channels in animal models of multiple sclerosis. Front. Immunol. 13, 976522. 10.3389/fimmu.2022.976522 36177027 PMC9513475

[B337] Yang Y. Wang D. Zhang C. Yang W. Li C. Gao Z. (2022e). Piezo1 mediates endothelial atherogenic inflammatory responses via regulation of YAP/TAZ activation. Hum. Cell 35 (1), 51–62. 10.1007/s13577-021-00600-5 34606042

[B338] Yang Y. L. Zhou C. Chen Q. Shen S. Z. Li J. D. Wang X. L. (2023). YAP1/Piezo1 involve in the dynamic changes of lymphatic vessels in UVR-induced photoaging progress to squamous cell carcinoma. J. Transl. Med. 21 (1), 820. 10.1186/s12967-023-04458-z 37974224 PMC10655279

[B339] Yang Z. Zhao Y. Zhang X. Huang L. Wang K. Sun J. (2024). Nano-mechanical immunoengineering: nanoparticle elasticity reprograms tumor-associated macrophages via Piezo1. ACS Nano 18 (32), 21221–21235. 10.1021/acsnano.4c04614 39079080

[B340] Yang Q. Cao Y. Wang L. Dong Y. Zhao L. Geng Z. (2025a). Mechanical force receptor Piezo1 regulates T(H)9 cell differentiation. Cell Rep. 44 (1), 115136. 10.1016/j.celrep.2024.115136 39932192

[B341] Yang J. Zhong J. Fu Z. He D. Zhang J. Yuan J. (2025b). Piezo1 enhances macrophage phagocytosis and pyrin activation to ameliorate fungal keratitis. Invest Ophthalmol. Vis. Sci. 66 (1), 33. 10.1167/iovs.66.1.33 39808118 PMC11737460

[B342] Yassouf M. Y. Zhang X. Huang Z. Zhai D. Sekiya R. Kawabata T. (2022). Biphasic effect of mechanical stress on lymphocyte activation. J. Cell Physiol. 237 (2), 1521–1531. 10.1002/jcp.30623 34724217

[B343] Ye Y. Barghouth M. Dou H. Luan C. Wang Y. Karagiannopoulos A. (2022a). A critical role of the mechanosensor PIEZO1 in glucose-induced insulin secretion in pancreatic β-cells. Nat. Commun. 13 (1), 4237. 10.1038/s41467-022-31103-y 35869052 PMC9307633

[B344] Ye X. Xia Y. Zheng Y. Chen W. Chen Z. Cheng Z. (2022b). The function of Piezo1 in hepatoblastoma metastasis and its potential transduction mechanism. Heliyon 8 (9), e10301. 10.1016/j.heliyon.2022.e10301 36097495 PMC9463386

[B345] Young K. M. Reinhart-King C. A. (2023). Cellular mechanosignaling for sensing and transducing matrix rigidity. Curr. Opin. Cell Biol. 83, 102208. 10.1016/j.ceb.2023.102208 37473514 PMC10527818

[B346] Young M. N. Sindoni M. J. Lewis A. H. Zauscher S. Grandl J. (2023). The energetics of rapid cellular mechanotransduction. Proc. Natl. Acad. Sci. U. S. A. 120 (8), e2215747120. 10.1073/pnas.2215747120 36795747 PMC9974467

[B347] Yu Z. Y. Gong H. Kesteven S. Guo Y. Wu J. Li J. V. (2022). Piezo1 is the cardiac mechanosensor that initiates the cardiomyocyte hypertrophic response to pressure overload in adult mice. Nat. Cardiovasc Res. 1 (6), 577–591. 10.1038/s44161-022-00082-0 39195867 PMC11358016

[B348] Yu H. Liu Z. Guo H. Hu X. Wang Y. Cheng X. (2024a). Mechanoimmune-driven backpack sustains dendritic cell maturation for synergistic tumor radiotherapy. ACS Nano 18 (34), 23741–23756. 10.1021/acsnano.4c08701 39158207

[B349] Yu H. Zhang Y. Shuai L. Peng C. Zhao C. Jiang Y. (2024b). Low hepatic artery blood flow mediates NET extravasation through the regulation of PIEZO1/SRC signaling to induce biliary complications after liver transplantation. Theranostics 14 (17), 6783–6797. 10.7150/thno.99514 39479458 PMC11519797

[B350] Yu L. Su Z. Tian D. Liu S. Zhang L. Wang Z. (2025). Piezo1 induces mitochondrial autophagy dysfunction leading to cartilage injury in knee osteoarthritis. Mol. Med. 31 (1), 272. 10.1186/s10020-025-01335-x 40753437 PMC12317449

[B351] Yuan W. Zhang X. Fan X. (2023). The role of the Piezo1 mechanosensitive channel in heart failure. Curr. Issues Mol. Biol. 45 (7), 5830–5848. 10.3390/cimb45070369 37504285 PMC10378680

[B352] Yuan G. Xiong Z. Ke X. Wang G. Liu X. Li Z. (2025). Exploring the multifactorial regulation of PIEZO1 in chondrocytes: mechanisms and implications. Int. J. Med. Sci. 22 (13), 3393–3411. 10.7150/ijms.111082 40765556 PMC12320791

[B353] Yue Y. Chen P. Ren C. (2024). Piezo1 modulates neuronal autophagy and apoptosis in cerebral ischemia-reperfusion injury through the AMPK-mTOR signaling pathway. Neurochem. Res. 50 (1), 32. 10.1007/s11064-024-04291-w 39585469

[B354] Zanconato F. Forcato M. Battilana G. Azzolin L. Quaranta E. Bodega B. (2015). Genome-wide association between YAP/TAZ/TEAD and AP-1 at enhancers drives oncogenic growth. Nat. Cell Biol. 17 (9), 1218–1227. 10.1038/ncb3216 26258633 PMC6186417

[B355] Zanconato F. Cordenonsi M. Piccolo S. (2016). YAP/TAZ at the roots of cancer. Cancer Cell 29 (6), 783–803. 10.1016/j.ccell.2016.05.005 27300434 PMC6186419

[B356] Zarychanski R. Schulz V. P. Houston B. L. Maksimova Y. Houston D. S. Smith B. (2012). Mutations in the mechanotransduction protein PIEZO1 are associated with hereditary xerocytosis. Blood 120 (9), 1908–1915. 10.1182/blood-2012-04-422253 22529292 PMC3448561

[B357] Zeng W. Z. Marshall K. L. Min S. Daou I. Chapleau M. W. Abboud F. M. (2018). PIEZOs mediate neuronal sensing of blood pressure and the baroreceptor reflex. Science 362 (6413), 464–467. 10.1126/science.aau6324 30361375 PMC6563913

[B358] Zeng Y. Riquelme M. A. Hua R. Zhang J. Acosta F. M. Gu S. (2022). Mechanosensitive piezo1 calcium channel activates connexin 43 hemichannels through PI3K signaling pathway in bone. Cell Biosci. 12 (1), 191. 10.1186/s13578-022-00929-w 36457052 PMC9716748

[B359] Zeng Z. Chen E. Xue J. (2025). Emerging roles of mechanically activated ion channels in autoimmune disease. Autoimmun. Rev. 24 (7), 103813. 10.1016/j.autrev.2025.103813 40194731

[B360] Zhan H. Xie D. Yan Z. Yi Z. Xiang D. Niu Y. (2024). Fluid shear stress-mediated Piezo1 alleviates osteocyte apoptosis by activating the PI3K/Akt pathway. Biochem. Biophys. Res. Commun. 730, 150391. 10.1016/j.bbrc.2024.150391 39002199

[B361] Zhang T. Chi S. Jiang F. Zhao Q. Xiao B. (2017). A protein interaction mechanism for suppressing the mechanosensitive piezo channels. Nat. Commun. 8 (1), 1797. 10.1038/s41467-017-01712-z 29176668 PMC5702604

[B362] Zhang C. Zhou T. Chen Z. Yan M. Li B. Lv H. (2020a). Coupling of integrin α5 to annexin A2 by flow drives endothelial activation. Circ. Res. 127 (8), 1074–1090. 10.1161/CIRCRESAHA.120.316857 32673515

[B363] Zhang L. Li Y. Ma X. Liu J. Wang X. (2020b). Ginsenoside Rg1-Notoginsenoside R1-Protocatechuic aldehyde reduces atherosclerosis and attenuates low-shear stress-induced vascular endothelial cell dysfunction. Front. Pharmacol. 11, 588259. 10.3389/fphar.2020.588259 33568993 PMC7868340

[B364] Zhang G. Li X. Wu L. Qin Y. X. (2021a). Piezo1 channel activation in response to mechanobiological acoustic radiation force in osteoblastic cells. Bone Res. 9 (1), 16. 10.1038/s41413-020-00124-y 33692342 PMC7946898

[B365] Zhang Y. Su S. A. Li W. Ma Y. Shen J. Wang Y. (2021b). Piezo1-Mediated mechanotransduction promotes cardiac hypertrophy by impairing calcium homeostasis to activate calpain/calcineurin signaling. Hypertension 78 (3), 647–660. 10.1161/HYPERTENSIONAHA.121.17177 34333987

[B366] Zhang M. Yan S. Xu X. Yu T. Guo Z. Ma M. (2021c). Three-dimensional cell-culture platform based on hydrogel with tunable microenvironmental properties to improve insulin-secreting function of MIN6 cells. Biomaterials 270, 120687. 10.1016/j.biomaterials.2021.120687 33540170

[B367] Zhang X. Hou L. Li F. Zhang W. Wu C. Xiang L. (2022). Piezo1-mediated mechanosensation in bone marrow macrophages promotes vascular niche regeneration after irradiation injury. Theranostics 12 (4), 1621–1638. 10.7150/thno.64963 35198061 PMC8825582

[B368] Zhang Y. Rózsa M. Liang Y. Bushey D. Wei Z. Zheng J. (2023a). Fast and sensitive GCaMP calcium indicators for imaging neural populations. Nature 615 (7954), 884–891. 10.1038/s41586-023-05828-9 36922596 PMC10060165

[B369] Zhang F. He X. Dong K. Yang L. Ma B. Liu Y. (2023b). Combination therapy with ultrasound and 2D nanomaterials promotes recovery after spinal cord injury via Piezo1 downregulation. J. Nanobiotechnology 21 (1), 91. 10.1186/s12951-023-01853-y 36922816 PMC10018903

[B370] Zhang Z. M. Yu P. Zhou K. Yu F. Y. Bao R. Y. Yang M. B. (2023c). Hierarchically porous implants orchestrating a physiological viscoelastic and piezoelectric microenvironment for bone regeneration. Adv. Healthc. Mater 12 (27), e2300713. 10.1002/adhm.202300713 37498795

[B371] Zhang T. Bi C. Li Y. Zhao L. Cui Y. Ouyang K. (2024a). Phosphorylation of Piezo1 at a single residue, serine-1612, regulates its mechanosensitivity and *in vivo* mechanotransduction function. Neuron 112 (21), 3618–3633.e6. 10.1016/j.neuron.2024.08.009 39270653

[B372] Zhang Q. Pan R. L. Wang H. Wang J. J. Lu S. H. Zhang M. (2024b). Nanoporous titanium implant surface accelerates osteogenesis via the Piezo1/Acetyl-CoA/β-Catenin pathway. Nano Lett. 24 (27), 8257–8267. 10.1021/acs.nanolett.4c01101 38920296 PMC11247543

[B373] Zhang J. Li J. Hou Y. Lin Y. Zhao H. Shi Y. (2024c). Osr2 functions as a biomechanical checkpoint to aggravate CD8(+) T cell exhaustion in tumor. Cell 187 (13), 3409–3426.e24. 10.1016/j.cell.2024.04.023 38744281

[B374] Zhang X. Leng S. Liu X. Hu X. Liu Y. Li X. (2024d). Ion channel Piezo1 activation aggravates the endothelial dysfunction under a high glucose environment. Cardiovasc Diabetol. 23 (1), 150. 10.1186/s12933-024-02238-7 38702777 PMC11067304

[B375] Zhang J. Zhao Y. Wu S. Gao L. Yang K. (2024e). Mechanosensing by Piezo1 in gastric ghrelin cells contributes to hepatic lipid homeostasis in mice. Sci. Signal 17 (859), eadq9463. 10.1126/scisignal.adq9463 39436995

[B376] Zhang X. Zhao Y. Li M. Wang M. Qian J. Wang Z. (2024f). A synergistic regulation works in matrix stiffness-driven invadopodia formation in HCC. Cancer Lett. 582, 216597. 10.1016/j.canlet.2023.216597 38145655

[B377] Zhang M. Wang Q. R. Hou X. Yang X. Zhou T. (2024g). Blockage of mechanosensitive Piezo1 channel alleviates the severity of experimental malaria-associated acute lung injury. Parasit. Vectors 17 (1), 46. 10.1186/s13071-024-06144-5 38303078 PMC10832208

[B378] Zhang D. D. He X. Y. Yang L. Wu B. S. Fu Y. Liu W. S. (2024h). Exome sequencing identifies novel genetic variants associated with varicose veins. PLoS Genet. 20 (7), e1011339. 10.1371/journal.pgen.1011339 38980841 PMC11233024

[B379] Zhang T. Li Y. Cheng B. Xu Z. Liu M. Feng J. (2025a). Piezo1 activation improves NSCLC liver metastasis immunotherapy by overriding matrix stiffness-mediated bimodal PD-L1/CXCL10 regulation. Adv. Sci. (Weinh) 12, e01335. 10.1002/advs.202501335 40583158 PMC12407327

[B380] Zhang X. Jiang E. Fu W. Wang Y. Wang Y. Fang Z. (2025b). Engineered endoplasmic reticulum-targeting nanodrugs with Piezo1 inhibition and promotion of cell uptake for subarachnoid hemorrhage inflammation repair. J. Nanobiotechnology 23 (1), 274. 10.1186/s12951-025-03305-1 40186204 PMC11971780

[B381] Zhang C. Zhang L. Chen L. Zheng Y. Zhang S. Guo S. (2025c). Mitigating effects of hydroxysafflor yellow a on atherosclerotic inflammatory responses based on flavonoid macromolecule compound: inhibition of Piezo1-YAP/JNK protein pathway. Int. J. Biol. Macromol. 309 (Pt 4), 142961. 10.1016/j.ijbiomac.2025.142961 40220830

[B382] Zhang F. R. Tang J. Lai Y. Lin Z. M. Lei Q. Q. (2025d). Smooth muscle cell Piezo1 is essential for phenotypic switch and neointimal hyperplasia. Br. J. Pharmacol. 182 (9), 2031–2048. 10.1111/bph.17436 39900041

[B383] Zhang Z. H. Zhu R. Liu Y. Wang F. F. Jiang A. Y. Dan R. C. (2025e). IL6-Dependent PIEZO1 activation promotes M1-Mediated orthodontic root resorption via CXCL12/CXCR4. J. Dent. Res. 104 (7), 763–773. 10.1177/00220345251316472 40077814

[B384] Zhao Q. Wu K. Chi S. Geng J. Xiao B. (2017). Heterologous expression of the Piezo1-ASIC1 chimera induces mechanosensitive currents with properties distinct from Piezo1. Neuron 94 (2), 274–277. 10.1016/j.neuron.2017.03.040 28426963

[B385] Zhao Q. Zhou H. Chi S. Wang Y. Wang J. Geng J. (2018). Structure and mechanogating mechanism of the Piezo1 channel. Nature 554 (7693), 487–492. 10.1038/nature25743 29469092

[B386] Zhao T. Huang Y. Zhu J. Qin Y. Wu H. Yu J. (2020)2025). Extracellular matrix signaling cues: biological functions, diseases, and therapeutic targets. MedComm 6 (8), e70281. 10.1002/mco2.70281 40686923 PMC12271642

[B387] Zhao W. Wei Z. Xin G. Li Y. Yuan J. Ming Y. (2021). Piezo1 initiates platelet hyperreactivity and accelerates thrombosis in hypertension. J. Thromb. Haemost. 19 (12), 3113–3125. 10.1111/jth.15504 34411418

[B388] Zhao X. Kong Y. Liang B. Xu J. Lin Y. Zhou N. (2022). Mechanosensitive Piezo1 channels mediate renal fibrosis. JCI Insight 7 (7), e152330. 10.1172/jci.insight.152330 35230979 PMC9057604

[B389] Zhao Y. Liu Y. Tao T. Zhang J. Guo W. Deng H. (2024). Gastric mechanosensitive channel Piezo1 regulates ghrelin production and food intake. Nat. Metab. 6 (3), 458–472. 10.1038/s42255-024-00995-z 38467889

[B390] Zhao J. X. Xu Y. Z. Fu H. X. Li J. Q. Yue M. Xu Z. Y. (2025a). SERCA2 regulates Piezo1 channel activation and contributes to the cardiac function and baroreflex in mice. Acta Pharmacol. Sin. 10.1038/s41401-025-01610-x 40629025 PMC12644713

[B391] Zhao Y. An Y. Wu F. Liu L. Tay F. R. Jiao Y. (2025b). Regulation of immune microenvironments by polyetheretherketone surface topography for improving osseointegration. J. Nanobiotechnology 23 (1), 199. 10.1186/s12951-025-03272-7 40069791 PMC11895393

[B392] Zheng M. Yao Y. Borkar N. A. Thompson M. A. Zhang E. Drake L. Y. (2024). Piezo channels modulate human lung fibroblast function. Am. J. Physiol. Lung Cell Mol. Physiol. 327 (4), L547–L556. 10.1152/ajplung.00356.2023 39189800 PMC11905809

[B393] Zhong G. Su S. Li J. Zhao H. Hu D. Chen J. (2023). Activation of Piezo1 promotes osteogenic differentiation of aortic valve interstitial cell through YAP-dependent glutaminolysis. Sci. Adv. 9 (22), eadg0478. 10.1126/sciadv.adg0478 37267365 PMC10413650

[B394] Zhong H. Zhou M. Guo J. Chen D. Xing C. Liu S. (2025). Ultrasound-driven wireless piezoelectric hydrogel synergizes with cotransplantation of NSCs-hUCMSCs for structural and functional recovery in spinal cord injury. Mater Today Bio 32, 101805. 10.1016/j.mtbio.2025.101805 40391024 PMC12088769

[B395] Zhou J. Zhou X. D. Xu R. Du X. Z. Li Q. Li B. (2021). The degradation of airway epithelial tight junctions in asthma under high airway pressure is probably mediated by Piezo-1. Front. Physiol. 12, 637790. 10.3389/fphys.2021.637790 33868003 PMC8047413

[B396] Zhou Z. Ma X. Lin Y. Cheng D. Bavi N. Secker G. A. (2023). MyoD-family inhibitor proteins act as auxiliary subunits of piezo channels. Science 381 (6659), 799–804. 10.1126/science.adh8190 37590348

[B397] Zhou Y. Li M. Lin S. Zhu Z. Zhuang Z. Cui S. (2025a). Mechanical sensing protein PIEZO1 controls osteoarthritis via glycolysis mediated mesenchymal stem cells-Th17 cells crosstalk. Cell Death Dis. 16 (1), 231. 10.1038/s41419-025-07577-1 40169556 PMC11961634

[B398] Zhou Y. Zhang W. Lin J. Zeng Y. Li Z. Wang P. (2025b). Mechanical stretch promotes the neutrophil recruitment potential of fibroblasts through the Piezo/NFAT1/LIF axis. Cell Signal 131, 111718. 10.1016/j.cellsig.2025.111718 40086612

[B399] Zhou S. L. Zhong L. L. Wu Y. L. Ji S. W. Li Y. Niu N. (2025c). The role of ion channels in the regulation of dendritic cell function. Cell Calcium 128, 103031. 10.1016/j.ceca.2025.103031 40253771

[B400] Zhu W. Guo S. Homilius M. Nsubuga C. Wright S. H. Quan D. (2022a). PIEZO1 mediates a mechanothrombotic pathway in diabetes. Sci. Transl. Med. 14 (626), eabk1707. 10.1126/scitranslmed.abk1707 34985971

[B401] Zhu Z. Li W. Gong M. Wang L. Yue Y. Qian W. (2022b). Piezo1 act as a potential oncogene in pancreatic cancer progression. Life Sci. 310, 121035. 10.1016/j.lfs.2022.121035 36208662

[B402] Zhu S. Chen W. Masson A. Li Y. P. (2024). Cell signaling and transcriptional regulation of osteoblast lineage commitment, differentiation, bone formation, and homeostasis. Cell Discov. 10 (1), 71. 10.1038/s41421-024-00689-6 38956429 PMC11219878

[B403] Zhu Y. Meng X. Zhai Q. Xin L. Tan H. He X. (2025a). Heavy mechanical force decelerates orthodontic tooth movement via Piezo1-induced mitochondrial calcium down-regulation. Genes Dis. 12 (2), 101434. 10.1016/j.gendis.2024.101434 39759122 PMC11697055

[B404] Zhu B. Li F. Yu J. Liang Z. Ke X. Wang Y. (2025b). PIEZO1 mediates matrix stiffness-induced tumor progression in kidney renal clear cell carcinoma by activating the Ca(2+)/Calpain/YAP pathway. Biochim. Biophys. Acta Mol. Cell Res. 1872 (1), 119871. 10.1016/j.bbamcr.2024.119871 39490703

[B405] Zhuang C. Gould J. E. Enninful A. Shao S. Mak M. (2023). Biophysical and mechanobiological considerations for T-cell-based immunotherapy. Trends Pharmacol. Sci. 44 (6), 366–378. 10.1016/j.tips.2023.03.007 37172572 PMC10188210

